# Cellular Snowballing: Cell Adhesion and Migration Drive the Self‐Assembly of Cell‐Microgel Biohybrid Spheroids

**DOI:** 10.1002/advs.202511302

**Published:** 2026-03-17

**Authors:** Zaman Ataie, Sina Kheirabadi, Changhao Li, Aneesh Risbud, Aswathy Sebastian, Istvan Albert, Sulin Zhang, Amir Sheikhi

**Affiliations:** ^1^ Department of Chemical Engineering The Pennsylvania State University University Park Pennsylvania USA; ^2^ Department of Engineering Science and Mechanics The Pennsylvania State University University Park Pennsylvania USA; ^3^ Department of Biomedical Engineering The Pennsylvania State University University Park Pennsylvania USA; ^4^ Huck Institutes of the Life Sciences The Pennsylvania State University University Park Pennsylvania USA; ^5^ Department of Biochemistry and Molecular Biology The Pennsylvania State University, University Park Pennsylvania USA; ^6^ Department of Materials Science and Engineering The Pennsylvania State University University Park Pennsylvania USA; ^7^ Department of Chemistry The Pennsylvania State University University Park Pennsylvania USA; ^8^ Department of Neurosurgery College of Medicine The Pennsylvania State University Hershey Pennsylvania USA

**Keywords:** granular hydrogel, living material, microgel, new approach methodologies, spheroid, tissue engineering

## Abstract

Creating three‐dimensional (3D) tissue models using cell spheroids that recapitulate the complicated structures and functions of human tissues is essential for advancing new approach methodologies used in drug testing/screening, disease modeling, and regenerative medicine. However, cell spheroids often have dense cellular structures and subsequently poor cell survival, primarily due to impaired oxygen and metabolite transport. To overcome these limitations, we develop biohybrid spheroids (BHS), self‐assembled living‐synthetic hybrid aggregates, using adherent cells as assembly engines and hydrogel microparticles (microgels) as extracellular matrix‐mimetic substrates. We show the revolving assembly of 3D BHS, driven by progressive cell migration and adhesion via culturing adherent mammalian cells and gelatin methacryloyl microgels, reminiscing a snowballing effect. The aggregation kinetics and terminal size of BHS are tailored by adjusting microgel size and cell‐to‐microgel ratio. Notably, microgels significantly larger than the cells yield porous, millimeter‐sized BHS, facilitating molecular diffusion and improving cell viability. Furthermore, transcriptional analyses show shifts in adhesion, angiogenesis, hypoxia, and proliferation programs in BHS compared with cell spheroids. An agent‐based model is developed to recapitulate the snowballing assembly in a geometrically unconstrained environment, providing fundamental insights into the assembly kinetics and the ultimate BHS size and pore features. BHS may open new opportunities for developing predictive and scalable technologies to self‐assemble large‐scale physiologically relevant tissue models in vitro, potentially transforming the biofabrication of microphysiological systems.

## Graphical abstract

A new class of biohybrid spheroids is engineered through the self‐assembly of adherent cells and extracellular matrix‐mimetic hydrogel microparticles (microgels). By mimicking a snowballing effect, this approach enables scalable formation of porous, millimeter‐scale spheroids with enhanced cell viability and molecular diffusion. An agent‐based model elucidates the assembly kinetics, offering a blueprint for bottom‐up construction of physiologically relevant 3D tissue models.

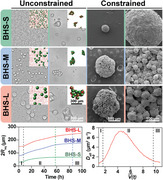

## 1 Introduction

The ability to fabricate functional tissues in vitro stands as a cornerstone in new approach methodologies (NAMs), which may be used in drug testing/screening, tissue/disease modeling, and regenerative medicine [1, 2, 3]. Traditional two‐dimensional (2D) cell culture models are inherently limited by their lack of structural complexity and spatial organization, which are defining features of native tissues composed of multiple cell types interacting with a dynamic extracellular matrix (ECM) [4]. Three‐dimensional (3D) spheroids have emerged as advanced analogs of in vivo microenvironments [5, 6] that enhance cell‐cell interactions and adhesion [7, 8, 9], thereby offering more physiologically relevant tissue architecture, function, and interactions with surroundings [10, 11]. Despite the potential of these aggregates as building blocks to assemble complex tissues [12, 13], spheroids fabricated by conventional techniques have limitations. The initial stages of cell spheroid formation may lack ECM, challenging the mimicry of native tissue microenvironments [14, 15]. Large spheroids ranging from a few hundred microns to a few millimeters suffer from limited oxygen and nutrient supply, leading to hypoxia in central regions that adversely affects cell survival and function [16]. These bottlenecks have impeded the scalable fabrication of functional cell spheroids [17, 18].

Microscale substrates, such as hydrogel microparticles (microgels), have been engineered to enhance cell‐cell and cell‐ECM interactions. Moreover, they have improved oxygen and nutrient transport to cells, thus enhancing viability [19]. These substrates have been used in granular hydrogel scaffolds [20], as carriers for cells or spheroids [21], and as oxygenating particles that further support cell survival [22]. The formation of cell‐microgel aggregates depends on multiple factors, including microgel physical properties (e.g., size [23, 24], curvature [25], and stiffness [26]) and biochemical characteristics (e.g., integrin‐binding ligands) [23, 27, 28], as well as the cell type. Cells with higher affinity to microgels than each other yield biohybrid cell‐microgel structures rather than cell‐only spheroids. As an example, Chinese hamster ovary cells expressing E‐cadherin form cell spheroids in the void spaces among jammed microgels because of strong cell‐cell adhesion [29]. Microgels with cell‐adhesive surface moieties, such as arginylglycylaspartic acid (RGD) peptide motifs [30, 31], promote cell adhesion through the integrin‐FAK‐ROCK‐myosin signaling pathway [23, 27]. Blocking this pathway impairs cell‐microgel assembly [23, 27]. In contrast, materials such as poly(lactic‐*co*‐glycolic acid) (PLGA) or agarose which lack RGD peptide motifs do not support the formation of cell‐microgel aggregates [30].

Microgel size and curvature are other key factors in regulating cell‐microgel assembly. Sub‐micron particles may be internalized by cells, but microgels larger than individual cells may aggregate into assemblies [24]. It has also been reported that myoblast cells are localized at microgel regions with higher curvature [25], and assemblies with interconnected pores support oxygen and nutrient diffusion [32], leading to improved metabolic activity and cell viability [24, 31, 33, 34, 35]. Cell‐microparticle aggregates have been used in diverse biomedical applications, as presented in Table S1, including in vitro stem cell differentiation [23] and treating critical limb ischemia in vivo [27]. Despite these advances, little is known about the effects of microgel size, cell adhesion, and cell migration on assembly kinetics and the final architecture of large‐scale cell‐microgel aggregates. In particular, the rate and mechanism of aggregation, as well as final size, geometry, metabolic profile, long‐term viability, and biological behavior of such living biohybrid constructs are yet to be understood.

In this work, we aim to investigate the formation mechanisms of biohybrid spheroids (BHS), comprising cells and microgels, in geometrically constrained and unconstrained environments, and how they may be engineered to address the limitations of conventional spheroids, such as scalability, cell viability, tissue heterogeneity, and insufficient initial cell‐matrix interactions. We use RNA sequencing (RNA‐seq) to profile gene expression in the BHS formed using varying microgel sizes relative to microgel‐free cell spheroids. Furthermore, we develop an agent‐based model (ABM) to afford a mechanistic understanding of BHS assembly kinetics and properties. This work may enable the development of large‐scale, viable, and metabolically active tissue models, potentially obviating the initial need for complex microvasculature.

## 2 Results and Discussion

### 2.1 Cell Adhesion and Migration Drive the Snowballing‐Like Assembly of BHS

Cells are provided with microscale ECM‐mimetic substrates, gelatin methacryloyl (GelMA), a protein‐based biopolymer, which is synthesized via reacting gelatin and methacrylic anhydride (MAA) [36], as shown in Figure S1. GelMA is a photocrosslinkable, biodegradable biopolymer with tunable physicochemical properties [37, 38]. Upon light exposure, a photoinitiator‐containing GelMA biopolymer solution forms a chemically crosslinked hydrogel, which is stable at the physiological condition (e.g., 37°C). As shown in Figure S2, GelMA contains amino acid sequences, such as RGD peptides, that promote cell adhesion by interacting with integrins on the cell surface [39, 40].

**FIGURE 1:**
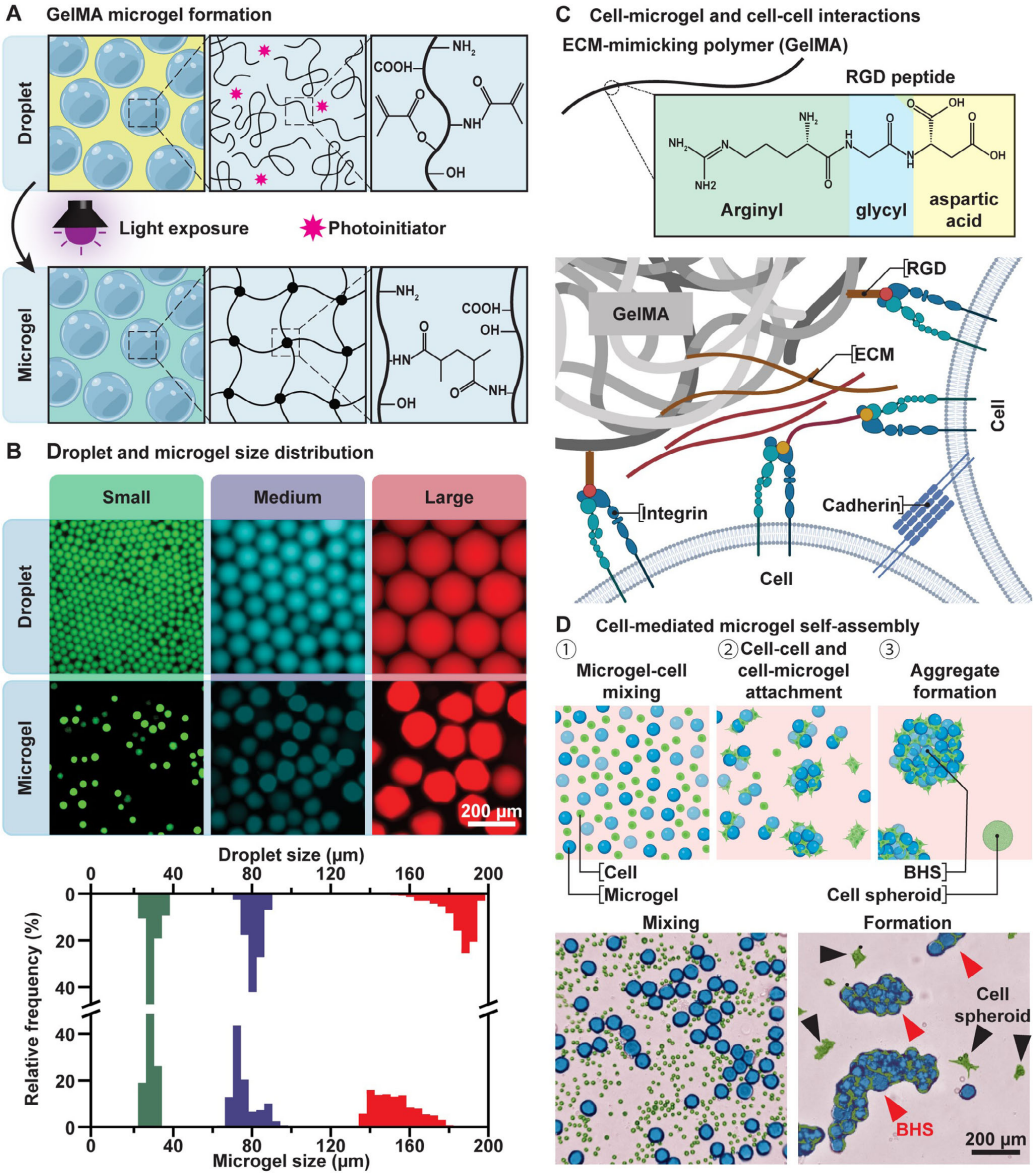
Formation of cell‐microgel assemblies. (A) GelMA droplets containing 5% w/v GelMA biopolymer are photochemically crosslinked to form stable microgels. (B) GelMA droplets and their corresponding microgels formed via photocrosslinking have three distinct sizes: small (green), medium (blue), and large (red). Fluorescent images show the size distribution of droplets and their corresponding photocrosslinked microgels. Histograms presenting the relative frequency (%) of each droplet and microgel size (number of analyzed droplets or microgels *n* > 1500). (C) Interactions between cells and ECM or GelMA influence the formation of cell aggregates, including cell spheroids and BHS. The adhesion is regulated by homophilic binding between cadherin moieties, and integrin‐mediated cell‐biomaterial/ECM interactions. (D) NIH/3T3 murine fibroblast cells and GelMA microgels are mixed to initiate cell‐mediated microgel assembly (step 1). Initially, cells adhere to microgels because of the adhesive moieties on GelMA (step 2). Cell spheroids are formed by cell‐cell aggregation, and BHS are yielded by cell‐microgel assembly (step 3). Pseudo‐colored optical microscopy images of cells and GelMA microgels, showing the formation of two types of aggregates.

GelMA microgels with a controlled size are fabricated using step emulsification microfluidic devices (Figure S3), which provide ECM‐mimetic substrates for cell adhesion. By adjusting the step sizes of microfluidic devices, droplets of varying diameters are produced (Figure S4) according to an established method [41, 42]. These droplets are then converted to stable microgels through the free‐radical photopolymerization of vinyl groups (Figure 1A). Figure 1B, shows the GelMA droplets, corresponding crosslinked microgels, and their size distribution. The average droplet diameter is 29 ± 3 (small), 81 ± 4 (medium), or 183 ± 11 µm (large). Microgels undergo size reduction during photocrosslinking to 28 ± 2, 76 ± 6, or 152 ± 10 µm for small, medium, or large droplets, respectively. The microgel size reduction post‐photocrosslinking originates from GelMA network densification, which leads to water release. The microgel size is selected to approximate or exceed the size of NIH/3T3 murine fibroblast cells (diameter ∼15 ± 2 µm in a cell suspension), as presented in Figure S5.

Mixing the microgels with the NIH/3T3 murine fibroblast cells in a geometrically unconstrained environment (planar, non‐adhesive substrate) prompts cell‐cell and cell‐microgel interactions. As schematically shown in Figure 1C, cell‐cell interactions are facilitated by homophilic cadherin‐cadherin binding [43] and integrin‐mediated binding to ECM components, such as fibronectin recycled by the cells [44]. Integrin‐mediated binding between cells and GelMA microgels, specifically RGD peptides and fibronectin, drives cell‐microgel interactions. Figure 1D shows the co‐culture of microgels and fibroblast cells. The top row schematically represents the three‐step assembly process: (i) initial mixing of cells and microgels, (ii) early‐stage attachment of cells to microgels because of the adhesive moieties on GelMA, and (iii) the formation of two distinct aggregate types. The bottom row shows the pseudo‐colored microscopy images of actual microgel‐free cell spheroids, arising from cell‐cell interactions, and BHS, which are formed through cell‐microgel assembly.

### 2.2 Microgel Size Regulates the Snowballing Kinetics and Terminal BHS Size

**FIGURE 2:**
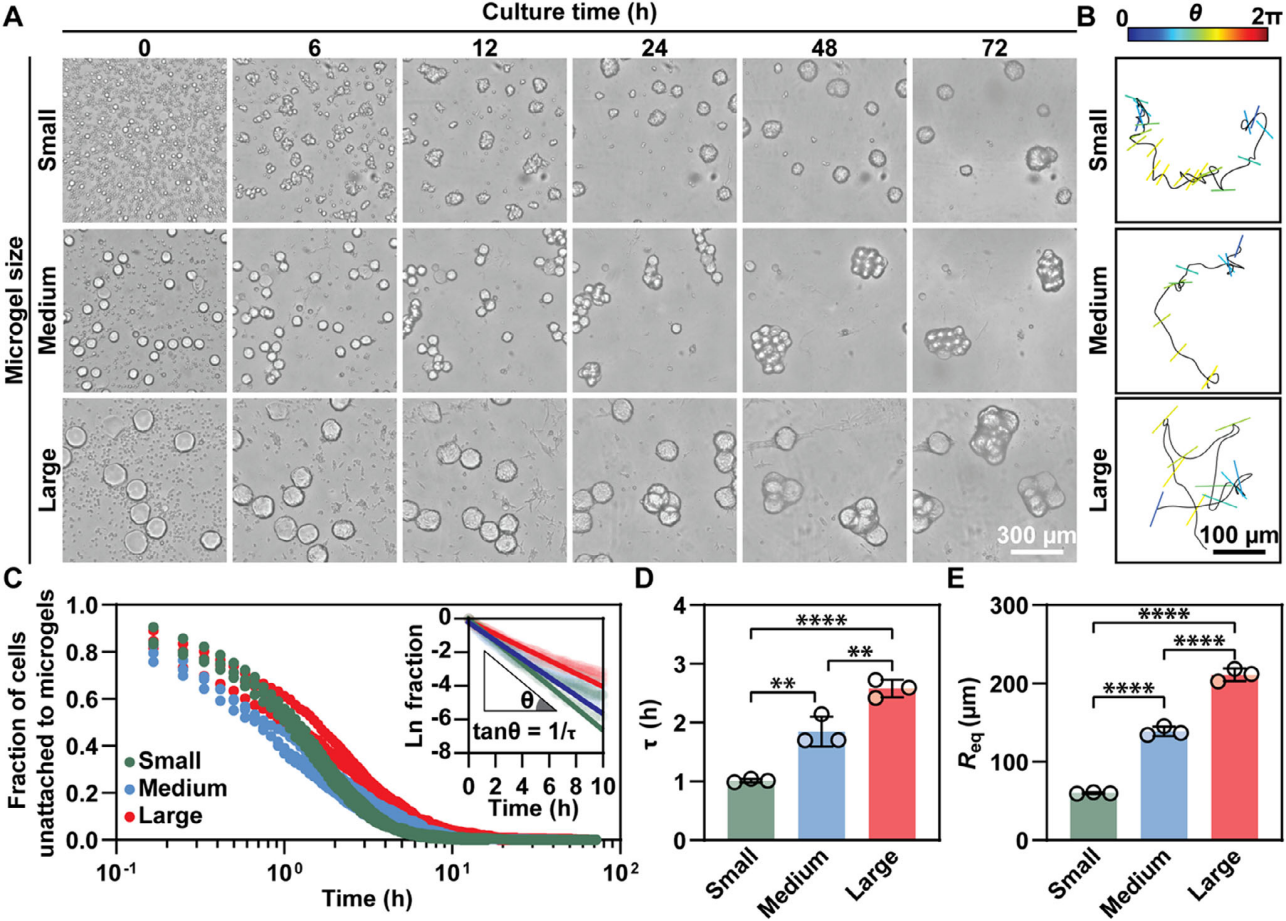
Kinetics of BHS formation. (A) Optical microscopy images of NIH/3T3 murine fibroblast cells, cultured with microgels of varying sizes in a geometrically unconstrained environment for 72 h. Scale bar: 300 µm. (B) Representative *x‐* *y* trajectories and relative angles for the selected microgel pairs. The black solid lines denote the trajectories of the center of mass, and the color‐coded line segments denote the relative angle θ between two microgels. (C) The fraction of cells unattached to microgels versus the time that cells are cultured with small, medium, or large microgels. Inset: the same data plotted in the logarithmic scale. The exponential fits are shown by the solid lines. (D) Characteristic decay time (τ) for the formation of BHS at varying microgel sizes. (E) The equivalent radius (*R*_eq_) of BHS, formed using the small, medium, or large microgels after ∼72 h of culture (*n* = 3). One‐way analysis of variance (ANOVA) is performed, followed by Tukey's post‐hoc multiple comparison test; **p < 0.01 and ****p < 0.0001.

Figure 2A shows the culture of fibroblast cells with microgels of three different sizes in a geometrically unconstrained environment for 72 h, resulting in BHS formation. As cell‐microgel interactions drive the aggregation and BHS formation, which depend on the accessible microgel surfaces, the total microgel surface area is maintained nearly constant in all the experiments. As shown in Figure S6, the total projected area for small, medium, and large microgels is ∼14 ± 1%, 15 ± 3%, and 14 ± 5%, respectively, showing no statistically significant differences. Real‐time videos (Videos S1–S3) of cell‐microgel assemblies show that BHS formation occurs primarily within ∼24 h, but the aggregates continue to move, connect, and merge upon contact at much slower rates. Interestingly, cell spheroids are simultaneously formed in microgel‐free regions, which may subsequently adhere to BHS upon contact. Control groups consisting of only microgels or cells are also monitored over time. Cell‐free microgels do not undergo any significant movement or aggregate formation (Video S4), showing cell mobility as an exclusive driving force for the aggregation. In contrast, the control sample comprising only cells undergoes cell spheroid formation over time (Video S5).

Our experimental observations indicate that the differential affinities between cells, microgels, and substrate are the drivers of BHS formation. The cells exhibit a higher integrin‐mediated affinity for microgel surfaces than for the untreated substrate. Since microgels are not self‐adherent, the cells act as essential binding agents in the cell‐microgel aggregation process (bio‐glue). As a cell migrates from the substrate to microgels, its adhesion and contractility drive both translational and rotational motions of the growing aggregate, resembling a snowballing process, facilitating the 2D‐to‐3D transition of cell‐microgel assemblies. Such rotational movement is evidenced by the dynamically changing angle of the line vector connecting two constituent microgels within the same BHS, relative to the fixed coordinates of the culture plane, as shown in Figure 2B and Videos S1–S3.

To register the kinetics of BHS formation, the fraction of cells unattached to microgels is quantified and shown in Figure 2C. A rapid exponential decline in the number of cells unattached to microgels is observed, which is fitted using an exponential decay model, $N\left(t\right)=N_{0}\exp\left(-t/\tau \right)$, where *N*(*t*) is the number of cells unattached to microgels at time *t*, *N*_0_ is the initial number of cells, and τ is the characteristic decay time. In all cases, *N*(*t*) drops to ≲4% of its initial value within ∼10 h, indicating a nearly complete cell adhesion to microgels. Under the same total surface area of microgels, *τ* increases by increasing the microgel size (Figure 2D), where *τ* is the time needed to reduce the number of cells unattached to microgels to 1/e of its initial value. The terminal size of BHS (after ∼72 h) is measured based on the equivalent radius $R_{\text{eq}}=\sqrt{A/\pi }$, where *A* denotes the projected area of an aggregate, and presented in Figure 2E. *R*_eq_ increases with microgel size, and the number density of stable BHS is higher for smaller microgels (Figure S7). Overall, the microgel size regulates the final aggregate size, which is kinetically arrested at long culture periods.

### 2.3 Agent‐Based Model (ABM) Reveals BHS Triphasic Formation Kinetics

**FIGURE 3:**
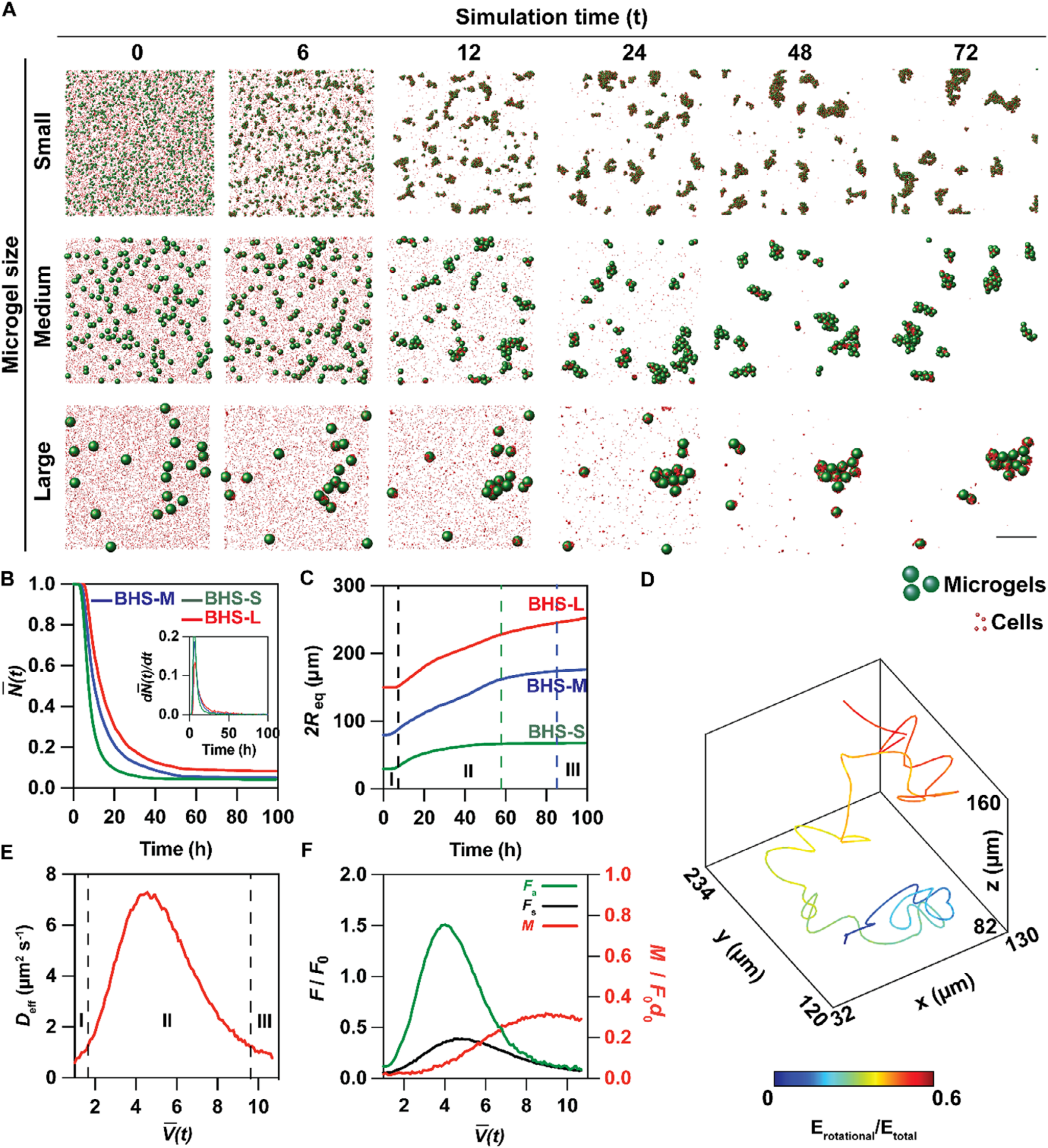
ABM of BHS formation. (A) Representative ABM results for varying sizes of microgels. Scale bar is 300 µm. (B) Time evolution of the normalized aggregate number $\bar{N}\left(t\right)=N\left(t\right)/N_{0}$ for small, medium, and large microgels, where *N*_0_ is the initial seeding number of microgels per unit area (∼1600, 230, and 50 mm^−2^ for small, medium, and large microgels, respectively). Inset: $d\bar{N}\left(t\right)/dt$ for each microgel size. (C) Time evolution of BHS equivalent diameter 2*R*_eq_ for varying microgel sizes, showing triphasic kinetics in the aggregation process. Stage I is the initial stage in which aggregation activity is low while cells adhere to individual microgels and move them around. In this stage, the rate of increase in *R*_eq_ is lower than 10% of the maximum rate for each microgel size. In Stage II, the aggregation rate sharply increases, peaks, and decreases, ending with a size‐arrested phase (Stage III, the absolute rate of decrease in *R*_eq_ is lower than 10% of the maximum rate for each microgel size). Stages I and II boundary is at *t* ∼5‐7 h (black dashed line), and the boundary between Stages II and III depends on the microgel size (green and blue dashed lines for BHS‐S and BHS‐M, respectively). BHS‐L does not reach Stage III within ∼100 h. (D) A representative trajectory of BHS‐M during the aggregation process. The color bar denotes the ratio between rotational kinetic energy about the center of mass and the total kinetic energy of an aggregate. (E) The equivalent diffusion coefficient for varying aggregate sizes, quantified by the normalized aggregate volume $\bar{V}\left(t\right)=V\left(t\right)/V_{0}$, where *V*(*t*) is the average volume of aggregates, and *V*_0_ is the volume of a single microgel. Here, medium microgels are used. (F) Normalized driving forces (*F*_0_ = 3  ×  10^−11^ N and *d*_0_ = 80 µm) and resistance force components for varying sizes of aggregates (quantified by the normalized aggregate volume $\bar{V}\left(t\right)$). Green curve (*F*_a_) is the magnitude of the net external force acting on the center of mass of an aggregate. Black curve (*F*_s_) shows the magnitude of the net substrate frictional force. Red curve (*M*) is the magnitude of net moment from all force components about the center of mass. The magnitude of driving forces, resistance force, and normalized aggregate volume are sampled over 3 simulations from 0‐72 h.

To elucidate the biophysical mechanisms governing the assembly kinetics and resulting structures of BHS, we developed an ABM to simulate the BHS formation process. In the ABM, both microgels and cells are treated as elastic spherical particles of varying radii *R_i_*. The shape evolution of cells during migration is neglected for simplicity. The substrate is simplified as a rigid surface with a lower affinity than cells to microgels, mimicking the differential cell adhesion observed in vitro. Pair‐wise inter‐particle interactions are described by the Johnson‐Kendall‐Roberts (JKR) contact mechanics [45]. The viscosity of culture medium and the substate‐particle friction endow a low Reynolds number environment in which particle dynamics are considered overdamped, and the inertial effect is negligible. The overdamped dynamics of each particle is characterized by its position vector $r_{i}$ and polarization vector ${\hat{n}}_{i}$:

(1) $$\eta \;\frac{dr_{i}}{dt}=F_{i,a}+\sum_{i\neq j}\left(F_{\mathrm{ij}}+F_{i,s}\right)+f_{r,i}\left(t\right)$$

(2) $$\eta R_{i}{\hat{n}}_{i}\;\times \;R_{i}\;\frac{d{\hat{n}}_{i}}{dt}=M_{i}+f_{\theta ,i}\left(t\right)$$

where η is the friction coefficient in the culture medium, $F_{i,a}=F_{i,a}{\hat{n}}_{i}$ is the self‐propulsion force of cells that originates from active cell contractility, $F_{\mathrm{ij}}$ and $F_{i,s}$ are the inter‐particle and particle‐substrate interaction forces, respectively, including elastic repulsion forces, adhesion forces perpendicular to contact surfaces, and parallel friction forces if contact surfaces have relative motions. $M_{i}$ is the net moment on the center of mass from all external forces. $f_{r,i}\left(t\right)$ and $f_{\theta ,i}\left(t\right)$ are Gaussian noise terms [46], which account for the stochastic fluctuations of translational and rotational cell movement, respectively. We calibrated and optimized the ABM parameters based on the cell migration experimental data as well as other reported measurements [47, 48, 49, 50, 51, 52, 53, 54]; see methods for detailed discussions. The experimentally benchmarked ABM quantitatively reproduces the key features of cell‐microgel aggregation, where individual cells rapidly attach to neighboring microgels/aggregates (Figure S8), and the BHS keep growing until stable (Figure 3A; Figures S9 and S10).

Using the ABM, we recapitulate the progression of BHS formation, characterized by the time‐varying number of aggregates (*N*(*t*)) normalized by the initial number of microgels (*N*_0_) for varying microgel sizes, as shown in Figure 3B. We observe that the aggregation kinetics of BHS distinctly vary with time and microgel size. Specifically, the decreasing rate of *N*(*t*)/*N*_0_ (Figure 3B, inset) slows down, while the stable equivalent diameter of aggregates (2*R*_eq_) (Figure 3C) increases over time when larger microgels are used. From the time‐course data of 2*R*_eq_, we discern triphasic kinetics in the aggregation process, comprising an initial period (Stage I) lasting ∼5‐7 h (∼5.1, 5.6, and 6.6 h for the small, medium, and large microgels, respectively) with low aggregation (BHS formation) activity while cells adhere to individual microgels and move them around, followed by a period (Stage II) where the aggregation rate sharply increases, peaks, and decreases, ending with a size‐arrested phase (Stage III), where the aggregation rate almost plateaus, and the aggregates reach a pseudo‐stable size at which most of them locally oscillate in isolation. The boundary between Stages I and II shows the time at which the rate of increase in *R*_eq_ is ∼10% of the maximum rate, and the boundary between Stages II and III shows the time at which the absolute rate of *R*_eq_ decrease is ∼10% of the maximum rate. Note that BHS‐L does not reach Stage III within the investigated period (∼100 h).

We rationalize the triphasic formation kinetics as follows. In Stage I, cells start exploring the environment and adhering to microgels, but no large aggregates are formed as the cells attach to one or only a few microgels while moving around on the 2D substrate. Thus, the aggregation rate is relatively low. During Stage II, the attached cells act as the active drivers of aggregation process. Cells attach to surfaces through focal adhesions, whereby any cellular active force is counterbalanced by the attached surface. For aggregation to proceed, attached cells must be in contact with the substrate to propel the movement of aggregate mass center. The attached cells that are not in contact with the substrate, thus being suspended on the aggregate, can only reorganize the internal structure of aggregates or induce localized oscillatory rotation by crawling on the microgel surfaces. As shown in Figure 2A–D, the rapid attachment of cells to multiple microgels corresponds to a sharp increase in aggregation rate and aggregate size, followed by a decrease in aggregation rate due to the depletion of cells at the microgel‐substrate interface, observed in Stage II. Stage III unfolds when the supply of unattached cells to microgels significantly diminishes, as cells exhibit a greater affinity to rich RGD‐containing microgels than the untreated substrate. The reduction in substrate‐attached cells leads to a progressive decline in the active propelling force emanating from the substrate. Consequently, the aggregation kinetics slow down markedly, eventually arrested at the final aggregate size.

To corroborate the above arguments, in Figure 3D and Figure S11, we first tracked the migration trajectories of medium microgels in ABM and experiments, which show that the motion of aggregates is a combination of translational (driven by substrate‐attached cells) and rotational (driven by cells only attached to microgels) motions as their sizes grow, akin to a snowballing process. The equivalent diffusion coefficient (*D*_eff_) of aggregates, shown in Figure 3E, supports the distinct triphasic kinetics and indicates the time evolution of aggregation driving forces. Aggregate‐level forces in the BHS are then quantified and shown in Figure 3F. The average net forces acting on the center of mass ($F_{a}=\frac{1}{N(t)}\sum_{i}\left|F_{a,i}+\sum_{j\neq i}F_{\mathrm{ij}}\right|$) and the net moment ($M=\frac{1}{N(t)}\sum_{i}\left|\begin{array}{c} M_{i} \end{array}\right|$), as well as the net substrate friction ($F_{s}=\frac{1}{N(t)}\sum_{i}\left|\begin{array}{c} F_{i,s} \end{array}\right|$) are separately measured. *F*_a_, depending on the number of aggregated cells attached to the substrate, significantly influences the aggregation rate. Conversely, *M* drives the rotational motion, and *F_s_* counteracts the motion of aggregates. As expected, *F_a_* shows an initial increase, correlated with the relative aggregation size (*R*_eq_), but finally vanishes when *R*_eq_ plateaus. Interestingly, despite the cells and microgels initially residing on a 2D substrate, this snowballing‐like aggregation process generates 3D BHS, driven by the combined effects of cell‐microgel adhesion and active cell locomotion. Indeed, as shown in Figure S11, the trajectories of microgels are first dominated by relatively straight motions, followed by gradually converting to spiral motion, indicating the rotation of aggregates.

### 2.4 BHS Architecture is Tailored by Geometric Constraints

**FIGURE 4:**
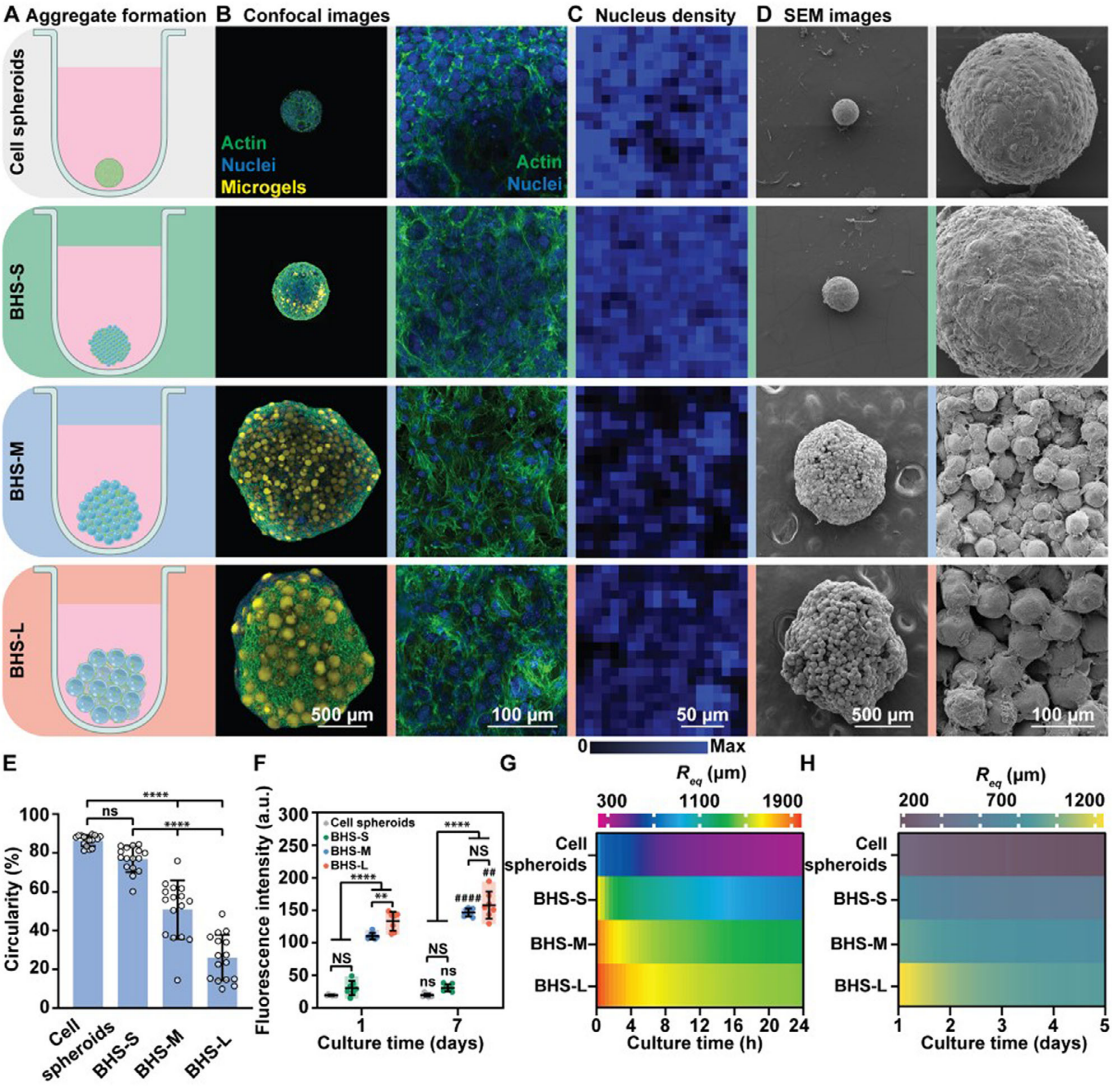
BHS and cell spheroid formation using NIH/3T3 murine fibroblast cells under geometric constraints. (A) BHS are formed by mixing cells with microgels of varying sizes in a geometrically constrained environment. Cell spheroids are formed similarly but without any microgels. (B) Confocal microscopy images of BHS or cell spheroids after 5 days, with actin filaments in green, nuclei in blue, and microgels in yellow. Colors are arbitrarily selected for clarity. (C) Nucleus density heatmap correlated with the confocal microscopy images. (D) SEM images of aggregates, showing that cell spheroids and BHS‐S have compact spherical shapes, while BHS‐M and BHS‐L are macroporous because of the larger building blocks. (E) The circularity of varying aggregates, showing that larger microgels decrease the aggregate circularity (*n* = 16). Ordinary one‐way ANOVA, followed by Tukey's post‐hoc multiple comparison test are performed (ns = not significant with *p ≥* 0.05 and ****p < 0.0001). (F) Metabolic activity of cells in aggregates, showing significantly higher values in BHS‐M and BHS‐L compared with cell spheroids and BHS‐S (*n* = 7). Two‐way ANOVA is performed, followed by Tukey's post‐hoc multiple comparison test. NS = not significant with *p ≥* 0.05, **p < 0.01, and ****p < 0.0001. For comparing days 7 with 1 of each sample, ns = not significant with *p ≥* 0.05, ##*p *< 0.01, and ####*p *< 0.0001. (G) *R*_eq_ heatmap of different aggregates during the first 24 h of culture. (H) *R*_eq_ heatmap of aggregates for days 1 to 5.

Cell‐microgel culture in a geometrically unconstrained environment results in irregularly shaped BHS and cell spheroids with a diverse size distribution, as shown in Figure S12A. Thus, an experimental system to enable large single BHS formation with pre‐determined cell and microgel number density is necessary to further assess the impact of microgels on cell behavior and aggregate properties, such as morphology and pore features. In contrast to unconstrained, freely formed cell‐microgel assemblies in a geometrically unconstrained environment, BHS with a more controlled and regular shape are formed by mixing the cells and microgels in a geometrically constrained environment (low‐attachment U‐bottom 96‐well plate) (Figure S12B). BHS‐S, BHS‐M, and BHS‐L, or cell spheroids are formed using small, medium, and large microgels, or microgel‐free cells, respectively, as schematically shown in Figure 4A. Confocal images of BHS and cell spheroids show that the BHS‐M and BHS‐L result in lower nuclear density and compactness compared with BHS‐S and cell spheroids (Figure 4B). Moreover, Figure 4C and Figure S13 show that the distance among cell nuclei is smaller in the cell spheroids and BHS‐S compared with the BHS‐M and BHS‐L, highlighting the increased cell compactness of the former aggregates.

Scanning electron microscopy (SEM) images of aggregates in Figure 4D show that cell spheroids and BHS‐S are smaller and more spherical in shape compared with the BHS‐M and BHS‐L. The circularity of BHS and cell spheroids is quantified in Figure 4E, showing that larger microgels yield assemblies with a lower circularity. Compactness in cell spheroids correlates with sphericity [55], as the formation of cell spheroids depends on collective forces exerted on cells [56]. In Figure 4F, we measure the metabolic activity of cells within aggregates on days 1 and 7, maintaining a constant initial cell density. Detailed statistical analyses corresponding to Figure 4F are provided in Figure S14, showing that BHS‐M and BHS‐L have a significantly higher metabolic activity compared with the cell spheroids and BHS‐S. Additionally, the metabolic activity and viability of cells within cell spheroids and BHS‐M are measured over an extended culture period of 14 days. Figure S15 presents cell viability and metabolic activity assessments over the extended culture period. As shown in Figure S15A,B, cell viability in BHS‐M (>99%) is significantly higher than that in cell spheroids (∼93%). Furthermore, while the metabolic activity of cell spheroids significantly decreases over time, the BHS‐M undergoes a significant increase in metabolic activity compared with day 1, indicating higher cell proliferation within the microgel‐containing aggregates (Figure S15C). The increased metabolic activity in these assemblies may also be correlated with their porous structure, as shown in Figure S16 and confirmed via modeling, which may facilitate oxygen and nutrient transfer. The porosity is presented in Figure S17, showing that BHS‐M and BHS‐L have approximately threefold and fivefold higher void fraction compared with BHS‐S, respectively. Furthermore, the cells within these aggregates appear more spread out (Figure 4C; Figure S13) compared with the compactness observed in BHS‐S and cell spheroids, potentially resulting in a higher metabolic activity.

To analyze the dynamic formation of geometrically constrained cell spheroids and BHS over time, the microscopy images of BHS and cell spheroids are acquired throughout a 5‐day period (Videos S6–S9). The control experiment with cell‐free medium microgels did not result in any aggregate formation (Video S10). Analyzing aggregate sizes over time highlights a trend: larger microgels require more time to reach an aggregate size plateau, because at a similar cell attachment rate (Figure 2B–D), larger microgels experience higher static friction and viscous forces due to an increased size and surface area. The most substantial BHS size change occurs within the initial 24 h (Figure 4G). The *R*_eq_ heatmap in Figure 4H presents long‐term size changes over five days of cell‐microgel culture, showing that the change in aggregate size is microgel size dependent, and the aggregate size eventually becomes kinetically arrested. BHS‐S reaches a stable size within ∼2 days (Figure S18A), BHS‐M requires ∼3 days (Figure S18B), and BHS‐L takes ∼4 days to reach a stable size (Figure S18C). Additionally, cell spheroid size is stabilized after ∼4 days (Figure S18D), and the control group containing solely microgels undergoes no size change within 5 days (Figure S18E).

Cells serve as both motors and adhesives agents in the BHS formation process; thus, we investigate their effects on BHS formation by manipulating the initial cell seeding density and monitoring BHS‐M formation. Evaluations through both brightfield (Figure S19) and fluorescence (Figure S20) imaging show a notable deceleration in BHS formation when the cell density decreases. As the cell count decreases, there is a shift toward an extreme case, resembling cell‐free microgels. In these cases, the necessary driving force for assembly, namely cells at microgel‐substrate interfaces, may be insufficient. Assemblies formed with fewer than 30000 cells after a 5‐day period lack sufficient cohesion, resulting in disintegration during handling, e.g., pipetting.

The effects of varying factors on BHS formation are investigated using the Box‐Behnken design of experiments, as shown in Figure S21. An experimental layout is shown in Figure S21A; 15 groups are defined by three continuous variables: cell density, microgel amount, and culture duration. The BHS size and the statistical significances are presented in Figure S21B,C, respectively. The response surface regression analysis yields an adjusted *R* ^2^ of ∼89%, indicating the degree of fit between the model and the observed data. The Pareto chart of standardized effects in Figure S21D shows that microgel concentration (variable B), cell concentration (variable A), and culture time (variable C) significantly affect BHS size, with notable interactions between cell concentration and culture time (variable AC). The squared terms for microgel concentration (variable BB), cell concentration (variable AA), and culture time (variable CC) indicate non‐linear (quadratic) relationships, showing that changes in these variables affect BHS size differently at varying levels. In Figure S21E, contour plots provide a visual representation of how these variables influence *R*_eq_. An increase in cell number and prolonged culture time reduce *R*_eq_, while more microgels correlate with an increase in BHS size.

### 2.5 Transcriptional Shifts of Adhesion, Angiogenesis, Hypoxia, and Proliferation Programs in BHS

**FIGURE 5:**
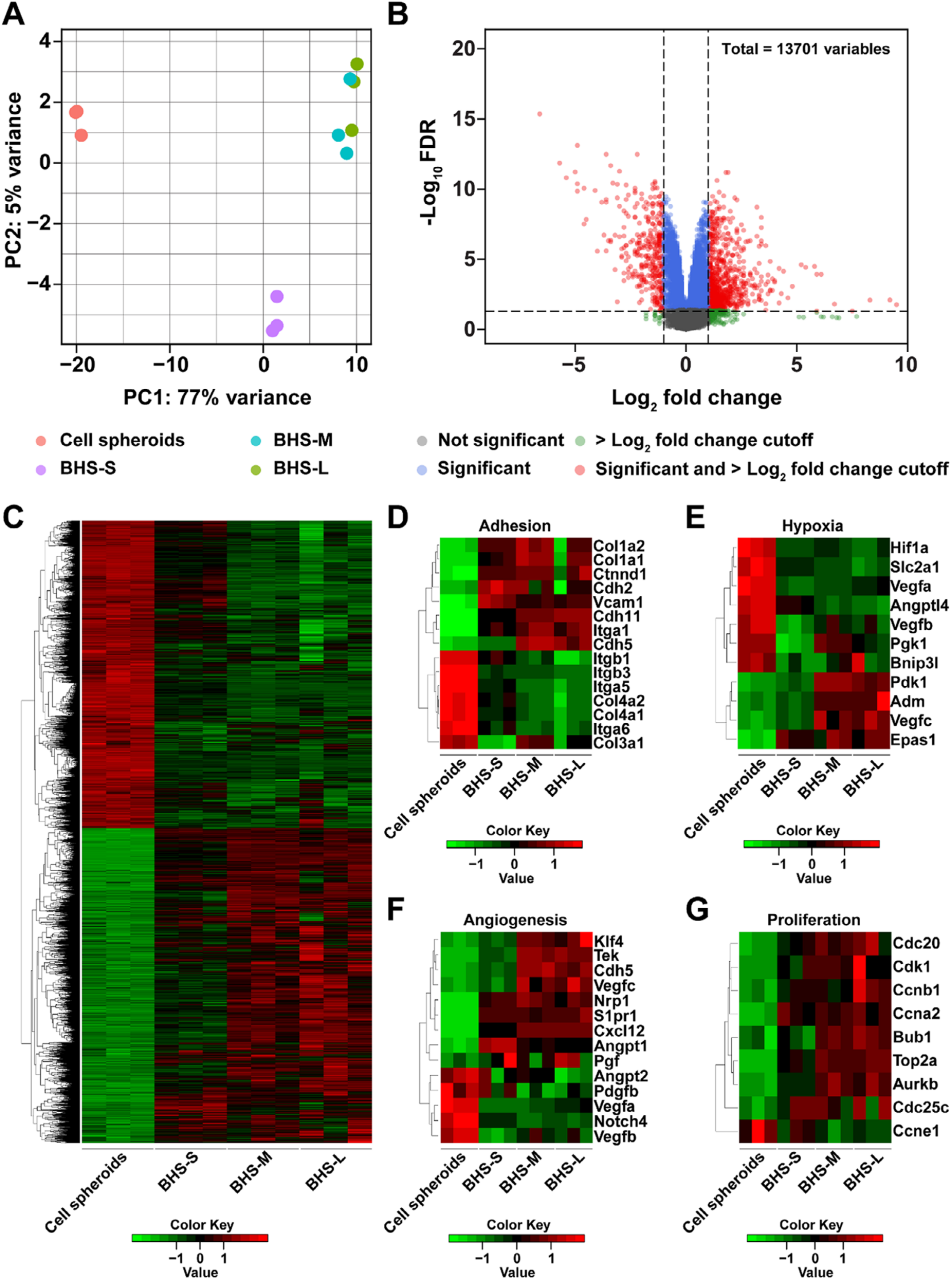
RNA‐seq analysis of NIH/3T3 murine fibroblast cell spheroids and BHS formed in a geometrically constrained environment and cultured for 5 days. (A) PCA of transcriptomic profiles across cell spheroids and BHS. (B) Volcano plot, showing DE genes between cell spheroids and BHS. (C) Heatmap of significantly regulated genes in the aggregates, formed with cells alone or BHS. Heatmaps of selected genes associated with (D) cell adhesion, (E) hypoxia, (F) angiogenesis, and (G) proliferation.

To evaluate gene expression profiles in cell spheroids and the BHS formed using varying microgel sizes and cultured in a geometrically constrained environment, RNA‐seq is performed. As presented in Figure 5A, principal component analysis (PCA) shows distinct transcriptomic differences between cells cultured as spheroids and those in the BHS. Replicates within each condition cluster closely, while cell spheroids are clearly separated from all BHS groups. Among BHS conditions, BHS‐M and BHS‐L cluster closer to each other, whereas BHS‐S shows greater separation from both BHS‐M and BHS‐L. Pairwise differential expression (DE) analysis identifies 6698 genes with FDR < 0.05 when comparing BHS to spheroids (Figure 5B). Pairwise comparisons between spheroids and BHS‐S, BHS‐M, and BHS‐L yield 6175, 7985, and 6479 significant genes, respectively. Consistent with these results, distinct gene expression patterns among cell spheroids, BHS‐S, BHS‐M, and BHS‐L are also observed in the heatmap of DE genes (Figure 5C).

Figure 5D–G presents the heatmap of selected genes associated with adhesion, hypoxia, angiogenesis, and proliferation functions. The normalized mean fold change of upregulated and downregulated adhesion‐related genes shows a size‐dependent trend (Figure S22A). For upregulated genes, mean fold change increases with aggregate size (cell spheroid < BHS‐S < BHS‐M) and then decreases in BHS‐L, while remaining higher than in BHS‐S. Conversely, the mean fold change of downregulated genes progressively decreases as aggregate size increases from spheroids to BHS‐L. Several adhesion genes are upregulated in BHS relative to spheroids (e.g., *Itga1, Cdh11*, and *Cdh5*), while others are downregulated (*Itgb3* and *Itga5*), as presented in Figure S23A. Upregulation of collagen (*Col1a1*, *Col1a2*) and cadherins (*Cdh11* and *Cdh5*) may suggest an organized matrix‐cell interface in BHS compared with spheroids. Enrichment analysis using g:Profiler provides a statistical framework to identify pathways and processes linked to DE genes [57, 58]. RNA‐seq and g:Profiler analyses reveal significant overrepresentation of adhesion‐ and ECM‐related GO biological process categories, including “cell‐cell adhesion” and “cell‐matrix adhesion” (Figure S24A). Together, these results indicate substantial shifts in adhesion and ECM programs in BHS relative to cell spheroids, possibly as a result of ECM‐mimetic microgels.

For hypoxia‐related genes, the normalized mean fold change of upregulated genes shows a direct relationship with aggregate size (Figure S22B). In addition, compared with cell spheroids, all BHS have lower normalized mean fold change values for downregulated genes, indicating reduced hypoxia‐associated transcriptional activity. This trend likely reflects improved oxygen diffusion within BHS due to the porosity. Canonical hypoxia markers (*Vegfa, Slc2a1, Hif1a*, and *Angptl4*) [57, 59] undergo reduced expression in BHS. In contrast, genes associated with hypoxia adaptation or vascular remodeling (e.g., *Epas1*, *Pdk1*, and *Vegfc*) [57, 59] are upregulated, which may suggest a regulated hypoxia‐associated transcriptional program rather than a uniform induction of canonical hypoxia markers (Figure S23B). Overall, canonical hypoxia‐response genes show a decreasing trend in BHS compared with spheroids. GO enrichment analysis further identifies significant enrichment of hypoxia‐related categories, including “cellular response to oxygen‐containing compounds” and “response to reactive oxygen species,” as presented in Figure S24B.

Similar to adhesion‐related genes, upregulated angiogenesis‐related genes have a size‐dependent trend, with normalized mean fold change increasing with aggregate size (cell spheroid < BHS‐S < BHS‐M) and then decreasing in BHS‐L, while remaining higher than in BHS‐S (Figure S22C). For downregulated genes, mean fold change decreases as aggregate size increases from spheroids to BHS‐L, indicating a size‐dependent angiogenic transcriptional program. DE analysis, presented in Figure S23C, shows changes in several angiogenic genes, including VEGF family members (*Vegfa, Vegfb, Vegfc, *and *Pgf*), angiopoietin/TIE signaling components (*Angpt1, Angpt2*, and *Tek*), and vascular/guidance markers (e.g., *Notch4* and *Klf4*) [57, 59]. Compared with spheroids, BHS aggregates downregulate some early pro‐angiogenic genes (e.g., *Vegfa* and *Notch4*) [57, 59] while upregulating genes associated with vessel maturation and stabilization (*Angpt1*, *Tek*, *Klf4*, *Vegfc*, and *Pgf*) [57, 59].

Finally, for proliferation‐related genes, the normalized mean fold change of upregulated genes in BHS increases by increasing microgel size, while the mean fold change of downregulated genes decreases from spheroids to BHS‐M and then slightly increases in BHS‐L (Figure S22D). Several cell‐cycle markers (*Ccnb1, Ccna2, Ccne1, Cdk1, Top2a, Aurkb, Bub1, Cdc20*, and *Cdc25c*) [57, 59] were significantly differentially expressed. These genes (except for *Ccne1*) were modestly higher in BHS, indicating maintained or increased proliferative potential in the BHS compared with cell spheroids (Figure S23D). The g:Profiler enrichment analysis confirms the significant enrichment of proliferation‐related GO biological process categories, including “cell cycle,” “mitotic cell cycle,” and “cell population proliferation” (Figure S24C). These results indicate that BHS aggregates maintain and may modestly enhance proliferative potential relative to cell spheroids.

Overall, combined DE and GO enrichment analyses show that BHS undergo (i) the enrichment of ECM organization and adhesion‐related pathways, consistent with a structured and tissue‐like architecture, (ii) the preservation of proliferative capacity, and (iii) the altered expression of hypoxia‐ and angiogenesis‐related genes across the BHS comprising varying microgel sizes compared with cell spheroids. Collectively, these transcriptional signatures indicate that, beyond morphological differences, BHS assembly is associated with gene expression programs reflecting enhanced structural integrity, vascular support, and regenerative potential.

### 2.6 BHS Formation Extends to Endothelial and Mesenchymal Stem Cells and Promotes Angiogenic Sprouting

**FIGURE 6:**
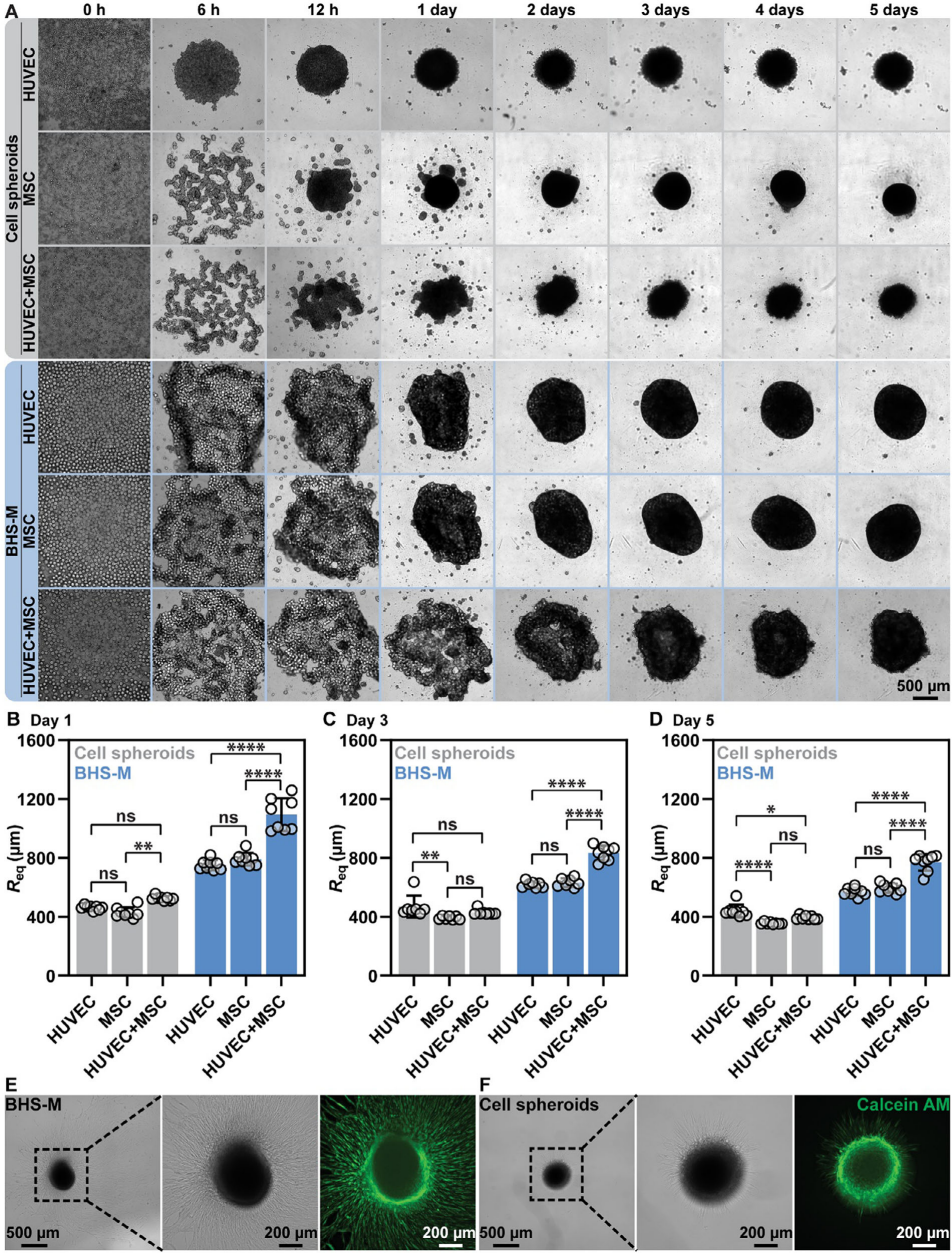
BHS formation using varying cell types. (A) Optical microscopy images of cell spheroids (top block) or BHS‐M (bottom block), each comprising HUVEC, MSC, or HUVEC+MSC in the top, middle, and bottom rows, respectively, over five days. *R*_eq_ ​measured at (B) day 1, (C) day 3, and (D) day 5, comparing cell spheroids or BHS‐M in gray or blue bars, respectively, for HUVEC, MSC, and HUVEC+MSC (*n* > 7). One‐way ANOVA is performed, followed by Tukey's post‐hoc multiple comparison test. ns = not significant with p ≥ 0.05, *p < 0.05, **p < 0.01, and ****p < 0.0001. (E) BHS‐M, composed of HUVEC and GelMA microgels, undergo markedly higher sprouting on Matrigel compared with (F) the HUVEC spheroids, formed using the same cell number. Both aggregate types are cultured for 5 days in a geometrically constrained environment, then transferred to a Matrigel‐coated substrate for an additional 3 days to assess vascular‐like outgrowth. Cells are visualized using Calcein AM (green).

To extend the BHS formation beyond contractile NIH/3T3 murine fibroblast cells, we investigate whether primary human umbilical vein endothelial cells (HUVEC), mesenchymal stem cells (MSC), and their mixtures can form similarly robust 3D assemblies with medium GelMA microgels (Figure 6A). Six groups of cells‐only or cell‐microgel assemblies are formed in a geometrically constrained environment: (i) cell spheroids of HUVEC, MSC, or HUVEC+MSC, and (ii) their corresponding BHS‐M, formed by mixing the cells with medium GelMA microgels.

Initially (0‐12 h), HUVEC‐only spheroids adopt a round shape, similar to our earlier observations with fibroblast cells, whereas MSC‐only and HUVEC+MSC spheroids show incomplete compaction. This difference arises from HUVEC homophilic cell‐cell adhesion (e.g., via VE‐cadherin), which drives early spheroid assemblies [60, 61]. In contrast, MSC relies more on de novo ECM secretion beside homophilic contacts (e.g., via N‐cadherin or Cadherin‐11), resulting in slower aggregation [62, 63, 64].

Figure 6B shows the differences in aggregate equivalent radius (*R*_eq_) on day 1. HUVEC+MSC spheroids appear as the largest among the cell‐only aggregates, and the HUVEC+MSC BHS form the largest assembly among the BHS groups. This behavior may be attributed to the heterotypic interactions between two cell types and the microgels, weaker cell‐microgel adhesion, and early tube‐like structure formation, previously reported in HUVEC‐MSC co‐culture with GelMA, that collectively drive cell spatial reorganization and may be more voluminous [65, 66]. By days 3 and 5 (Figure 6C,D), HUVEC spheroids are relatively larger than MSC or HUVEC+MSC spheroids, likely because HUVEC, especially in pro‐angiogenic media, favor hollow or less‐dense spheroid architectures and secrete less ECM than MSC and fibroblasts [67]. In contrast, MSC‐only and HUVEC+MSC spheroids progressively densify, influenced by continued ECM deposition [68]. Meanwhile, the HUVEC+MSC BHS persistently feature the largest size among the BHS conditions. Overall, these results indicate that BHS assembly extends beyond highly contractile fibroblast cells to other adherent cells, such as HUVEC and MSC, although the final size and aggregation kinetics vary.

The functional outcomes of these HUVEC‐based assemblies are further evaluated by examining angiogenic sprouting (Figure 6E,F). In both HUVEC spheroids and BHS, a decrease in core area is observed. For HUVEC spheroids seeded on Matrigel, the equivalent core diameter decreases from ∼880 ± 86 µm at seeding to ∼353 µm after 3 days of sprouting. Similarly, in HUVEC BHS, the equivalent core diameter decreases from ∼1141 ± 56 µm at seeding to ∼460 µm on day 3. This reduction in core size is likely due to spheroid sprouting. Additionally, compared with conventional HUVEC spheroids, which yield limited outgrowth (equivalent diameter of core plus sprouts ∼764 µm; number of sprouts ∼98 ± 17) because of dense cell packing and inhibited metabolite diffusion, HUVEC BHS undergo more pronounced vascular‐like branching (equivalent diameter of core plus sprouts ∼2068 µm; number of sprouts ∼263 ± 84). This likely results from the microgel‐mediated BHS porosity that supports molecular transport and cellular metabolism, along with cell migration within BHS, promoting angiogenic sprouting in vitro.

### 2.7 BHS as Versatile Building Blocks for Large Tissue‐Like Structures in vitro

Current hydrogel‐based in vitro tissue models larger than a few hundreds of microns require perfusable blood vessels or channels to overcome the diffusion limitation of oxygen/metabolites and maintain cell viability [69]. Here, we assess BHS as building blocks for developing mm‐scale tissue‐like structures without requiring blood vessels. A critical criterion in developing large tissues in vitro is cell viability throughout the construct. We have shown that BHS made up of medium microgels have significantly higher cell viability (Figure S25A,B) and metabolic activity (Figure S25C) compared with the microgel‐free cell spheroids.

The fabrication of tissue‐like structures through a bottom‐up approach requires the integration of these building blocks into a larger, unified assembly. Note that this assembly is different from the snowballing‐like aggregation which is initiated from the co‐seeding of single cells and microgels. We investigate this fusion/assembly by manually placing different types of BHS or cell spheroids in contact in a geometrically constrained environment, followed by culturing them for five days. The results are shown in Figure S26, where all types of aggregates undergo a decrease in total projected area, indicative of fusion. Figure S27 shows the quantification of such area reduction by day 5, reporting 38 ± 8, 28 ± 4, 23 ± 2, and 23 ± 1% for cell spheroids, BHS‐S, BHS‐M, and BHS‐L, respectively. The area reduction for cell spheroids and BHS‐S is statistically significant by day 2 compared with day 1, whereas for BHS‐M, a significant reduction was observed by day 3. BHS‐L, however, does not undergo any significant decrease in area over the 5‐day period. These findings suggest that the dynamics of fusion are influenced by the aggregate size, with larger aggregates requiring more time to achieve notable fusion. Finally, to assess the suitability of BHS as building blocks for constructing large tissue‐like structures over extended culture periods, BHS‐M are fused to form millimeter‐scale tissue constructs, and cell viability is assessed, showing that cells remain highly viable (>99%) after 2 weeks of culture, as shown in Figure S28.

To further test the degree of fusion and explore the level of modularity in building tissues, live murine fibroblast cells are labeled with either a green or a red fluorescent dye, followed by incubation with medium microgels in a geometrically unconstrained environment for three days. These differently colored aggregates are manually added together on a flat surface and allowed to fuse over three days, as shown schematically in Figure S29A. This approach confirms the feasibility of fusion in a geometrically unconstrained environment and shows that the aggregates of different colors merge effectively (Figure S29B). The successful integration of distinctly labeled aggregates suggests that BHS enables modular structure fabrication. This modularity may enable the incorporation of varying cell types or biomaterials, potentially broadening BHS applications in creating complex, heterogeneous tissue‐like structures.

To evaluate the structural integrity and rheological properties of fused tissue‐like constructs, BHS are manually placed in contact in a flat‐bottom cylindrical container and cultured for 3 days, resulting in the formation of large solid‐like, mechanically robust constructs (diameter = 10 mm and height = 1 mm), which are easy to handle and manipulate (Figure S30A). Oscillatory shear rheology tests are conducted to probe the viscoelastic behavior of these constructs (Figure S30B–D). Figure S30B presents a strain sweep test at a constant frequency of 1 rad s^−1^, establishing the linear viscoelastic region (LVR) and the cross‐over point at which storage and loss moduli equate. Figure S30C shows the frequency sweep results within the LVR at 0.1% strain. Figure S30D shows that the storage modulus of fused BHS is ∼3065 ± 242 Pa at 0.1% strain and 1 rad s^−1^ frequency, which is two to three orders of magnitude higher than that of the cell‐free microgel suspension control (∼27 ± 22 Pa), indicating the formation of solid‐like, cohesive, and viscoelastic constructs.

**FIGURE 7:**
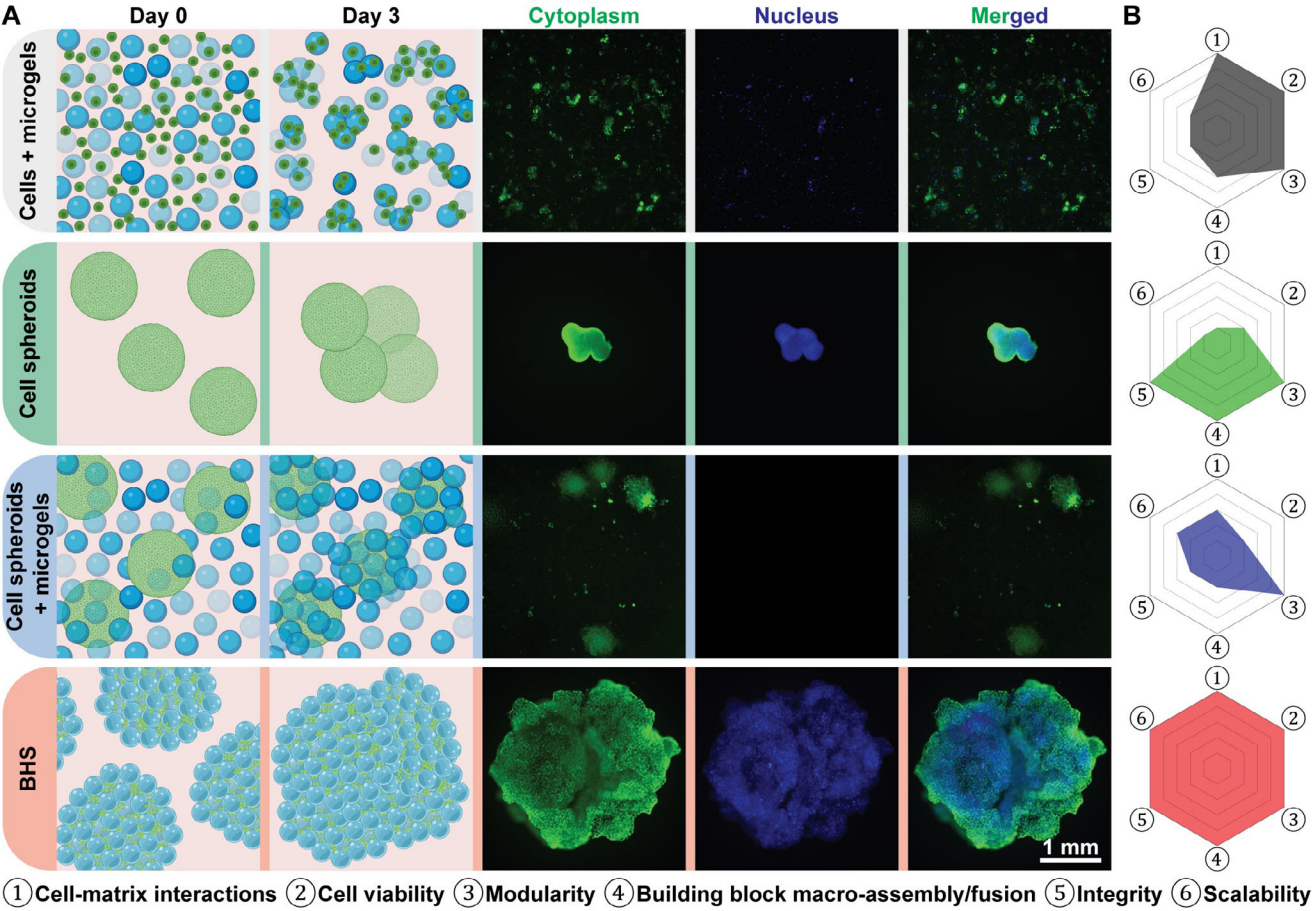
Formation of tissue‐like structures in vitro using varying building blocks in a geometrically unconstrained environment. (A) Schematics and fluorescence images of building blocks, including cells and microgels, cell spheroids, cell spheroids and microgels, or BHS, cultured for 72 h to form tissue‐like constructs. Upon transferring and gentle pipetting, only the cell spheroids and BHS maintain their structural integrity and cohesion, while the other two groups disintegrate. (B) Radar plots, comparing the advantages of BHS as building blocks with other study groups based on cell‐matrix interactions, cell viability/metabolic activity, modularity, building block macro‐assembly/fusion, structural integrity, and scalability.

Varying building blocks, including cells, microgels, cell spheroids, and BHS are used to generate tissue‐like structures within 72 h, as presented in Figure 7A. For a better comparison, four aggregates (cell spheroids/BHS‐M) or the equivalent quantities of their constituents (cells/microgels) are compared. The initial configuration entails a mixture of cells and microgels, allowed to form BHS‐M in a geometrically unconstrained environment. The design variables are cell density (480000 cells, equivalent to ∼4 cell spheroids/BHS‐M), microgel amount (108 µL, equivalent to 4 BHS‐M), and culture time (72 h) using which the aggregates of initial configuration reach *R*_eq_ of ∼139 ± 82 µm. Changing the variables may result in the formation of varying aggregate sizes, which have been reported [26, 32]. The second configuration involves fusing cell spheroids on a flat untreated surface, which yields constructs with *R*_eq_ of ∼330 ± 18 µm. While cell spheroids have been used as building blocks for tissue engineering scaffolds and biofabrication [70, 71], their inherent limitations, such as hypoxic core formation and compactness/density, render the fabrication of viable large tissue‐like structures non‐trivial, unless they are perfusable or vascularized [72].

The third configuration involves cell spheroids and microgels, which have been reported for tissue engineering applications [73]. For the formation of a tissue‐like structure, the connectivity among microgels and cell spheroids is crucial, which is regulated by the ratio of cell spheroids to microgel size/number density [73]. In our experiments, using four cell spheroids leads to the microgel accumulation on the cell spheroid surface. This microgel coating impedes cell spheroid fusion, blocking the formation of mm‐scale constructs. Finally, BHS are allowed to contact, resulting in mm‐scale (*R*_eq_ ∼1339 ± 119 µm) fused tissue‐like structures that are easily pipetted after five days (Video S11) or two weeks of culture (Video S12), indicative of the unique capability of BHS for large‐scale tissue fabrication with sizes reaching up to 4 mm in diameter in this work. Altogether, radar plots in Figure 7B compare BHS as building blocks for fabricating large tissue‐like constructs in vitro with cells + microgels, cell spheroids, and cell spheroids + microgels based on six criteria, including cell‐matrix interactions, cell viability, modularity, building block macro‐assembly/fusion, structural integrity, and scalability for tissue formation. These qualitative radar plots rank each type of aggregate on a 1‐5 ordinal scale (1 = least, 5 = most) across the six criteria. Scores are assigned from experimental observations in the manuscript and Supporting Information, including live/dead (Figures S25 and S28) and metabolic activity assays (Figure 4E; Figures S15,S25), fusion tests (Figures S26–S28), handling behavior during transfer (Videos S11,S12), and the successful formation of mm‐scale constructs (Figure 7; Figures S26–S30).

## 3 Conclusions

This study investigates the self‐assembly and properties of BHS, which address some of the limitations of conventional spheroids. Although conventional 3D spheroids are considered as a significant improvement over monolayer 2D cell cultures, they often fail to mimic the complexity of native tissues and lack scalability as a result of impeded oxygen and metabolite transfer. We show co‐culturing NIH/3T3 murine fibroblast cells with GelMA spherical microgels of varying sizes in a geometrically unconstrained environment initiate cell‐mediated microgel assembly. Cell migration and adhesion to the microgels result in a self‐driven 3‐step BHS formation, which resembles a snowballing process. Based on experimental results and ABM, the complex interfacial interactions and cellular forces that drive the BHS assembly process are identified. Additionally, BHS formation kinetics and resulting architectures in both geometrically constrained and unconstrained environments are thoroughly investigated. When cells and microgels are co‐cultured in a geometrically unconstrained environment, varying shapes and sizes of assemblies are obtained. Interestingly, geometric constraints yield a single BHS. Microgel size plays a significant role in tailoring BHS structure; Larger microgels yield more porous BHS and enhance metabolic functions and cell viability because of improved molecular diffusion, although these assemblies require longer formation times compared with those made up of smaller microgels. Gene expression patterns in BHS indicate changes in the structural integrity, vascular support, and regenerative capacity of aggregates compared with cell spheroids. Taken together, this study sheds light on the fundamental and applied aspects of fabricating tissue‐like large‐scale constructs through bottom‐up cell‐microgel assembly. The predictable kinetics, size, and porosity of BHS may provide new opportunities for large‐scale tissue biofabrication, with potential applications in NAMs for precision medicine, drug testing and screening, and tissue engineering and regeneration.

## 4 Experimental Section

### 4.1 Materials

Novec 7500 Engineered Fluid was purchased from 3M (MN, USA). HUVEC and NIH/3T3 murine fibroblast cells were purchased from ATCC (VA, USA). BRAND microplates (BRANDplates, inertGrade, low‐binding, 96 wells, 330 µL, round bottom, transparent) were purchased from BRAND GMBH + CO KG (Germany). Corning Matrigel Matrix was purchased from Corning (NY, USA). HyClone characterized fetal bovine serum (FBS) and HyClone penicillin‐streptomycin 100× solution (P/S) were purchased from Cytiva (UT, USA). Polydimethylsiloxane (PDMS, SYLGARD 184 silicone elastomer kit) was purchased from Dow Corning (MI, USA). Biopsy punches (1.5 mm with plunger system) were purchased from Integra Miltex (NY, USA). KMPR 1000 series photoresists were purchased from Kayaku Advanced Materials (MA, USA). EGM‐2 Endothelial Cell Growth Medium‐2 BulletKit was purchased from Lonza (Switzerland). Ultra‐pure water (Milli‐Q, electrical resistivity ∼18 MΩ cm at 25°C) was obtained from the Direct‐Q 5 UV remote water purification system, Millipore Corporation (MA, USA). Mesenchymal stem cell growth medium 2 was purchased from PromoCell (Germany). Disposable cell scrapers (Biologix, disposable, polyethylene, sterile, handle length: 180 mm, blade length: 18 mm), micro centrifuge tubes (Celltreat, 1.5 mL, clear, polypropylene), centrifuge tubes (Celltreat, 15 mL, sterile), and cell strainers (Celltreat, 40 µm, polypropylene, sterile) were purchased from Neta Scientific Inc. (NJ, USA). Deuterium oxide (D_2_O, deuteration degree 99.95%), gelatin (type A from porcine skin, gel strength ∼300 g Bloom), Methacrylic anhydride (MAA, contains 2000 ppm topanol A as inhibitor, 94%), lithium phenyl‐2,4,6‐trimethylbenzoylphosphite (LAP, >95%), fluorescein isothiocyanate(FITC)‐dextran (average molecular weight = 2 MDa), trichloro(1H,1H,2H,2H‐perfluorooctyl)silane (F‐silane, 97%), 4‐(4,6‐Dimethoxy‐1,3,5‐triazin‐2‐yl)‐4‐methylmorpholinium chloride (DMTMM, ≥97%), and Human MSC (Bone Marrow) were purchased from Sigma. Pico‐Surf (1% in Novec 7500) was purchased from Sphere Fluidics (UK). 1H,1H,2H,2H‐perfluoro‐1‐octanol (PFO) and a variety of cell culture and staining reagents including Gibco Dulbecco′s phosphate buffered saline (DPBS, 1x, no calcium, no magnesium), Dulbecco's modified Eagle medium (DMEM), trypsin‐EDTA (0.25%, with phenol red), Pierce 16% formaldehyde (w/v, methanol‐free), PrestoBlue cell viability reagent, LIVE/DEAD cell imaging kit (contains Calcein AM, cell permeant dye as live cell indicator, and BOBO‐3 Iodide as dead cell indicator), Alexa Fluor 647 hydrazide, Hoechst 34580, CellTracker Fluorescent Probes (Green CMFDA and Red CMTPX Dyes), and 4',6‐diamidino‐2‐phenylindole (DAPI) were purchased from Thermo Fisher Scientific (MA, USA). Dialysis membrane (12‐14 kDa molecular weight cutoff) was purchased from Spectrum Laboratories (NJ, USA). Silicon wafers (100 mm diameter, 500 µm thickness) were purchased from UniversityWafer, Inc., MA, USA. Sterile disposable filter units (filter capacity 500 mL, pore size 0.2 µm), disposable pipetting reservoirs, plain microscope slides (thickness 1 mm, length × width = 75 mm × 25 mm), Petri dishes (sterile, 60 mm × 15 mm, polystyrene), and ethanol (200 proof/100%) were purchased from VWR (PA, USA). RNeasy Plus Mini Kit and QIAshredder were purchased from Qiagen (MD, USA). Illumina Stranded mRNA library prep kit was purchased from Illumina (CA, USA). The KAPA Library Quantification kit was purchased from KAPA Biosystems (MA, USA).

### 4.2 Gelatin Methacryloyl (GelMA) Synthesis

Gelatin methacryloyl (GelMA) was synthesized according to our previously established protocol [74]. DPBS (200 mL) was heated to 50°C, and 20 g of gelatin was dissolved via stirring at 200 rpm. MAA (16 mL) was then added dropwise to the mixture, being stirred at 50°C. The reaction was stopped after 3 h by adding 400 mL of DPBS. The solution was then dialyzed against ultra‐pure water for 10 days at 40°C to remove unreacted MAA. A clear solution was obtained, sterile filtered using filtration units, and frozen at ‐80°C. Finally, the frozen GelMA was lyophilized using Labconco FreeZone 4.5L ‐84 C Benchtop Freeze Dryer (Labconco Corporation, MO, USA) at a collector temperature of ‐82.4°C and pressure ∼0.009 mbar to yield a white solid.

### 4.3 Proton Nuclear Magnetic Resonance (^1^H NMR) Spectroscopy

GelMA synthesis was confirmed by ^1^H NMR spectroscopy using a 500 MHz Bruker NEO instrument (MA, USA) at the Pennsylvania State University NMR facility. Gelatin and GelMA samples (40 mg each) were dissolved in 2 mL of D_2_O and heated at 37°C for 2 h to ensure complete dissolution. Vinyl group peaks, indicative of methacryloyl modification and absent in gelatin (Figure S1), were identified between 5.5 to 6.5 ppm using TopSpin software (version 4.0.7, Bruker, MA, USA).

### 4.4 Fluorescent Labeling of GelMA

To conjugate GelMA with a fluorophore, 1 g of GelMA biopolymer was dissolved in 50 mL of DPBS under continuous stirring at 200 rpm and 40°C until fully dissolved. Then, 22 mg of DMTMM was introduced into the mixture, followed by the addition of Alexa Fluor 647 hydrazide solution (48 µL). The conjugation reaction was allowed to proceed at 40°C for 2 h. Then, the solution was dialyzed against ultra‐pure water for 3 days at 40°C to remove unreacted components. After dialysis, the solution was frozen at ‐80°C, followed by lyophilization to obtain the fluorophore‐conjugated GelMA in a solid form. The lyophilized product was stored in the dark.

### 4.5 Microfluidic Device Fabrication

High‐throughput step emulsification microfluidic devices were fabricated at the nanofabrication facilities of the Pennsylvania State University [41, 42]. Two‐ or three‐layer master molds were fabricated on silicon wafers using the KMPR 1000 series as the negative photoresists. The first layer was spin‐coated with KMPR 1005, KMPR 1025, or KMPR 1035 for the mold fabrication of small, medium, or large droplets, respectively, according to the manufacturer guidelines. This resulted in layer heights of 8, 27, or 60 µm, respectively. Following this, subsequent layers were deposited using KMPR 1035, designed to be 2‐3 times the size of anticipated droplets, to provide ample space for droplet formation and mobility. The devices were then fabricated using the PDMS by mixing the base and crosslinker at a ratio of 10:1, vacuum degassing, pouring onto the nanofabricated molds, degassing again, and curing at 80°C for 2 h. The devices were then punched with a 1.5 mm biopsy punch for the inlets and outlets, bonded to a glass microscope slide after air plasma treatment at 400 mTorr for 45 s using Plasma Cleaner (Harrick Plasma, PDC‐001‐HP), followed by an F‐silane (2 vol% in Novec 7500 Engineered Fluid) treatment. The treated devices were rinsed with Novec 7500 Engineered Fluid and then heated in an oven at 80°C for 30 min to evaporate residual oil.

### 4.6 Microgel Fabrication

Lyophilized GelMA was dissolved in DPBS, containing a photoinitiator (LAP at a final concentration of 0.1% w/v), at 40°C to prepare a 5% w/v aqueous GelMA biopolymer solution. A mixture of Novec 7500 Engineered Fluid, containing 2% v/v Pico‐Surf surfactant, was used as the oil phase for the small and medium droplets, and a 0.5% v/v surfactant in the same oil was used for the large droplets. The droplet fabrication system was maintained at around 35°C‐40°C using a space heater. Then, droplets were maintained at 4°C overnight to form physically crosslinked microgels. As detailed in Figure S31, microgels were then photocrosslinked via light exposure (wavelength = 395‐400 nm, intensity = 15 mW cm^−2^) for 5 min, followed by adding an equal volume of PFO (20% v/v in Novec 7500 Engineered Fluid), vortexing for 5 s, and centrifuging at 300 × *g* for 15 s to remove the oil and surfactant. To ensure that no residual oil or surfactant remained, the microgel suspension was rinsed with an equal volume of DPBS, vortexed, and centrifuged at 300 × *g* for 15 s.

### 4.7 Cell Culture

NIH/3T3 murine fibroblast cells, primary HUVEC, or MSC were cultured in DMEM (supplemented with 10% v/v FBS and 1% v/v antibiotics), EGM‐2, or complete mesenchymal stem cell growth media, respectively. The medium was refreshed every other day, and the cells were passaged when they reached ∼80% confluency, typically twice a week. A standard cell culture incubator (Eppendorf, Hamburg, Germany) was used to culture cells under a 5% v/v carbon dioxide (CO_2_) atmosphere at 37°C. The cells were trypsinized (detached from the culture dish) using a 0.25% trypsin‐EDTA solution, followed by counting using an automated cytometer (Countess 2, ThermoFisher Scientific, MA, USA).

### 4.8 BHS and Cell Spheroid Formation

To form BHS without geometric constraints, crosslinked GelMA microgels were packed at 3000 × *g* and then pipetted into a Petri dish (60 mm) using a positive displacement pipette (MICROMAN E M100E, Gilson Company, Inc., OH, USA). To ensure a similar total surface area, 10, 27, or 50 µL of a packed small, medium, or large microgel suspension was used, as shown in Figure S6. A cell suspension (3 million cells per Petri dish) was also added in 5 mL of media, resulting in a cell concentration of 600000 cells mL^−1^ (Figure S32). The microgels and cells were mixed by gentle pipetting. To form BHS under geometric constraints, GelMA microgels were packed at the similar conditions mentioned above. Then, microgels and cells were mixed, while maintaining a constant cell density of 600000 cells mL^−1^ and varying the microgel amount according to their size (2, 5.4, or 10 µL mL^−1^ for small, medium, or large, respectively, Figure S33). The cell–microgel suspensions were gently pipetted and transferred into disposable pipetting reservoirs. Then, 200 µL of the mixture was added to each well of a U‐bottom 96‐well plate. The culture was maintained for up to one week, with the media being refreshed daily. Cell spheroids were formed similarly to the BHS, but without the inclusion of microgels.

### 4.9 Analysis of BHS and Cell Spheroid Formation

A CytoSMART Lux2 cell imaging microscope (CytoSMART Technologies, Netherlands), equipped with a 10x objective, was used to image BHS formation in a geometrically unconstrained environment over time. Images were acquired at 5‐min intervals for 72 h and used to determine morphological and kinetic parameters. The area of each BHS was calculated using the ImageJ software (Fiji, version 1.54f, NIH, MD, USA) [75]. The attachment of cells to microgels and/or other cells during BHS or cell spheroid formation was assessed by tracking them using a Mathematica script (Wolfram Mathematica, version 13.3, Wolfram Research, IL, USA). BHS formation in the geometrically constrained environment was imaged using the Incucyte S3 Live‐cell Analysis system (version 2022A, Sartorius, Germany) at Sartorius cell culture facilities, the Pennsylvania State University, with brightfield, and green‐fluorescence protein (GFP) channels. Images were acquired every 30 min for 5 days using a 4x objective and analyzed using the Incucyte spheroid analysis software module (version 2022A, Sartorius, Germany).

### 4.10 Microgel Tracking

Microgels were tracked using a custom Python script via the *trackpy* library [76]. The stacked images were loaded, and particles were initially detected in the first frame. The parameters for particle detection, including diameter and minimum mass threshold, were optimized based on the microgel size to ensure accurate identification. Particle tracking was performed frame by frame. The trajectories were visualized, and short‐lived tracks persisting for fewer than 10 frames were filtered out to ensure reliability in the subsequent analyses.

### 4.11 Quantification and Analysis of Single Cells during Aggregate Formation

The attachment of cells to microgels and/or other cells during BHS or cell spheroid formation was assessed by tracking them using a Mathematica script (Wolfram Mathematica, version 13.3, Wolfram Research, IL, USA). For each microgel size, an exponential decay function was fitted to the number of cells unattached to microgels over the initial number of cells within approximately 10 h. This fitting used the exponential decay model as

(3) $$N\left(t\right)=N_{0}e^{-\frac{t}{\tau }}$$

where *N*(*t*) is the number of cells unattached to microgels at time *t*, *N*_0_ is the initial number of cells, and *τ* is the characteristic decay time.

### 4.12 Porosity Characterization

BHS were formed in a geometrically constrained environment for 3 days, followed by incubation in a FITC‐dextran solution (M_w_ ∼2 MDa, 30 µm in DPBS) for 10 min to fill voids. Porosity was determined from 3D *Z*‐stacked images (volume of interest *x* × *y* × *z* ∼150 × 150 × 68 µm^3^), acquired using a Leica STELLARIS 5 confocal microscope (Leica Microsystems, Germany). The LAS X (version 5.0.3, Leica Microsystems, Germany) software calculated the void volume fraction based on the ratio of fluorescently labelled void spaces to the total volume.

### 4.13 Cell Staining Procedures

Cells were visualized using fluorescent staining. BHS and cell spheroids were fixed with 4% paraformaldehyde for 2 h at room temperature, followed by permeabilization with 0.2% Triton X‐100 in DPBS for 2 h. Then, aggregates were washed with DPBS at least five times, each for 10 min. For actin filament staining, Phalloidin Alexa Fluor 488 was applied (1:40 volume ratio in DPBS) and incubated overnight in darkness at 4°C. Nuclear staining was conducted using DAPI (1:1000 volume ratio in DPBS) for 2 h. Samples were imaged using a Nikon AXR confocal microscope (Nikon, Japan). Live cell labeling was performed using CellTracker Green CMFDA and Red CMTPX, according to the manufacturer's protocol on 2D cultured cells. Additionally, live cell nuclei were stained with Hoechst (1:2000 volume ratio in PBS) for 10 min. Samples were imaged using a DMi8 THUNDER Imager 3D Cell Culture microscope or a Leica STELLARIS 5 confocal microscope (Leica Microsystems, Germany).

### 4.14 Scanning Electron Microscopy (SEM)

The surface morphology of aggregates was investigated using a scanning electron microscope (Quanta 250 ESEM, Thermo‐Scientific, OR, USA) at the materials characterization lab of the Pennsylvania State University. Cell spheroids or BHS were formed in 3 days, then fixed in 4% v/v of paraformaldehyde for 3 h. The fixed samples were rinsed at least five times with DPBS and subsequently immersed in a gradient of ethanol solutions with concentrations ranging from 15% to 100% (v/v in ultra‐pure water). The samples were then dried using a critical point dryer (CPD300, Leica EM, Germany) to ensure the complete removal of any fluid. Finally, the samples were sputter‐coated with iridium (thickness ∼2‐5 nm, Emitech K575 Turbo sputter coater, E.M. Technologies, UK) and imaged with a beam current of 91 pA under an accelerating voltage of 5 keV using an Everhart‐Thornley detector (ETD) in the Secondary Electron (SE) mode.

### 4.15 Cell Metabolic Activity Assessment

The cell metabolic activity solution was prepared by adding PrestoBlue cell viability solution to DMEM (serum‐free) in a 1:9 volume ratio. The BHS cultured in a geometrically unconstrained environment was removed using a cell scraper, then moved into a 15 mL centrifuge tube. The tube was centrifuged for 5 min at 300 × *g*. After discarding the supernatant, 3 mL of the metabolic activity solution was added to the centrifuged BHS. The tubes were wrapped in aluminum foil and incubated for 4 h in a cell culture incubator at 37°C, with 5% CO_2_. Then, the tubes were re‐centrifuged at 300 × *g* for 5 min, and the supernatant was collected for analysis. Fluorescence intensity was recorded using a microplate reader (Tecan Infinite M Plex, Switzerland) at 560 nm excitation and 590 nm emission wavelengths. The metabolic activity of BHS formed in a geometrically constrained environment was measured using the PrestoBlue cell viability solution mixed with an equal volume of serum‐free DMEM. Then, 50 µL of this stock solution was added to each well. The plates were incubated at 37°C with 5% CO_2_ for 4 h, followed by fluorescence intensity measurement using the aforementioned device and conditions.

### 4.16 Cell Viability Assessment

Using a cell scraper, all cells, microgels, and/or formed aggregates were detached from the Petri dish and transferred to centrifuge tubes. The tubes were centrifuged at 300 × *g* for 5 min, followed by discarding the supernatant. Cell viability in the BHS or cell spheroids was assessed using a two‐color fluorescence LIVE/DEAD cell imaging kit. The imaging solution, made from Calcein AM as a live cell indicator and BOBO‐3 Iodide for staining dead cells, was prepared according to the manufacturer's protocol, followed by adding 1 mL into each centrifuge tube and resuspending via gentle pipetting. The centrifuge tubes were then wrapped in an aluminum foil and placed under the biosafety cabinet at room temperature for 30 min and imaged using the Leica DMi8 fluorescence microscope (THUNDER imaging systems, Leica Microsystems, Germany). The live cell channel was set to 470 nm (blue) excitation and 510 nm (green) emission wavelengths. The dead cell channel was set to 550 nm excitation and 610 nm emission wavelengths. Images were analyzed using ImageJ software (Fiji, version 1.54f, NIH, MD, USA) [75], and cell viability was reported as the number of live cells over the total number of cells. For measuring the viability of cell spheroids, BHS‐M, and fused BHS‐M, formed in a geometrically constrained environment, the culture media were carefully removed, and 200 µL of the imaging solution was added to each well. The plates were incubated at room temperature for 30 min and imaged using the Leica STELLARIS 5 confocal microscope (Leica Microsystems, Germany) at the excitation and emission wavelengths mentioned earlier. Images were analyzed using ImageJ software (Fiji, version 1.54f, NIH, MD, USA) [75], and cell viability was reported as the area of live cells over the total area of live and dead cells.

### 4.17 Angiogenic Sprouting Assay

The sprouting assay was performed using HUVEC spheroids or BHS. A constant cell density (600000 cells mL^−1^) was used in both cases, and in the case of BHS, 5.4 µL mL^−1^ of medium microgels were mixed with the cell suspension. Then, 200 µL of the mixture was added to each well of a U‐bottom 96‐well plate. The culture was maintained for 5 days. Matrigel was thawed overnight at 4°C, coated onto a 48‐well plate, and cured at 37°C for 4 h. Assemblies (on day 5) were cultured on top of the Matrigel for 3 days, stained with the two‐color fluorescence LIVE/DEAD cell imaging kit, and imaged using the Leica DMi8 fluorescence microscope (THUNDER imaging systems, Leica Microsystems, Germany). The equivalent diameter of spheroid/BHS core plus sprouts was calculated using ImageJ software (Fiji, version 1.54f, NIH, MD, USA) [75]. The number of sprouts was counted independently by three blinded observers, and the results are reported as the mean ± standard deviation of their measurements.

### 4.18 RNA Isolation and Next Generation Sequencing

BHS or cell spheroids were prepared in a geometrically constrained environment for 5 days. Aggregates were then collected, and a pooled sample set was prepared, containing at least 45 aggregates from each study group. Culture media were carefully removed, and samples were washed three times with DPBS. After the final wash, samples were centrifuged at 500 × *g* for 1 min, and the remaining supernatant was removed. RNA was isolated using the RNeasy Plus Mini Kit following the manufacturer's instructions. Briefly, 350 µL of Buffer RLT Plus was added to the samples, and the suspension was vortexed for 30 s. The entire suspension was then transferred to a QIAshredder spin column and centrifuged at 21300 × *g* for 2 min. The homogenized lysate was transferred to a gDNA eliminator spin column and centrifuged at 8000 × *g* for 30 s. The flow‐through was mixed with 350 µL of 70% ethanol and pipette‐mixed. The mixture was transferred to a RNeasy spin column and centrifuged at 8000 × *g* for 15 s. After discarding the flow‐through, 700 µL of Buffer RW1 was added and centrifuged at 8000 × *g* for 15 s. The flow‐through was discarded, and 500 µL of Buffer RPE was added and centrifuged at 8000 × *g* for 15 s. This step was repeated one more time, followed by a centrifugation at 8000 × *g* for 2 min. To further dry the membrane, the column was centrifuged again at 21300 × *g* for 1 min. Finally, 50 µL of RNase‐free water was added to the column, and RNA was eluted by centrifugation at 8000 × *g* for 1 min. Samples were stored at ‐80°C.

For quality control, total RNA samples were evaluated by the Pennsylvania State University Huck Institutes’ Genomics Core Facility using Agilent TapeStation 4150 analysis (Agilent). All samples had an RNA Integrity Number (RINe) above 9.4. For RNA‐seq, total RNA samples were submitted to the Genomics Core Facility, and libraries were prepared using the Illumina Stranded mRNA library prep kit. Following library preparation, library quality and size were evaluated with the DNA 5000 Screen Tape on the Agilent TapeStation 4150 (Agilent), and the concentration was determined via qPCR using the KAPA Library Quantification kit. The libraries were pooled at equimolar amounts and sequenced on the Illumina NextSeq 2000 (Illumina) in single‐end mode using a P2 100‐cycle kit at 1 × 100 nt, generating ∼35 million reads per sample on average. Three biological replicates (*n* = 3) were used for each study group.

### 4.19 RNA‐Seq Data Processing and Analysis

Libraries were sequenced on an Illumina NextSeq 2000 (Illumina) to generate single‐end 100 bp reads. FastQC [77] was used to examine base‐quality profiles and adapter content. Reads were aligned to the Mus musculus mm10 reference genome using Hisat2 [78]. The average mapping rate of samples was 99%. Aligned data were visualized and inspected in Integrative Genome Viewer (IGV) [79]. Transcript‐level abundances were quantified with Salmon [80] and summarized into gene‐level counts. Normalization and differential expression analysis were performed with edgeR using the generalized linear model framework [81]. Normalized expression values were converted to *z‐*scores to visualize the expression pattern in heatmaps. Functional enrichment analysis of significant genes was carried out using g:Profiler [57].

### 4.20 Fusion Test

BHS or cell spheroids were prepared in a geometrically constrained environment for 3 days. Four aggregates of the same type (either BHS‐S, BHS‐M, BHS‐L, or cell spheroids) were transferred and placed in proximity within a U‐bottom well. Media were refreshed daily. Brightfield images were captured every day using an EVOS XL Core microscope (Thermo Fisher Scientific, MA, USA). The area of fused aggregates was measured using ImageJ software (Fiji, version 1.54f, NIH, MD, USA) [75] and reported.

### 4.21 Formation of mm‐Scale Tissue‐Like Structures

BHS‐M were cultured in a geometrically unconstrained environment for 3 days. Subsequently, these aggregates were detached using a cell scraper and passed through a cell strainer (40 µm pores) to remove unbound cells and debris, ensuring the retention of BHS‐M on the strainer due to their size. The collected aggregates were then placed into a custom‐designed PDMS cylindrical mold (12 mm diameter, 3 mm height), ensuring close contact among them. Following an additional 3 days of culturing with daily media refreshment, a large mm‐scale BHS‐M was formed, which could be readily removed using a spatula.

### 4.22 GelMA Bulk Hydrogel Scaffold Fabrication

GelMA bulk hydrogel scaffolds were fabricated using a two‐step crosslinking process, mirroring the method for microgel fabrication, to ensure that they share similar physicochemical properties. Scaffolds were prepared by dissolving GelMA biopolymer in a 0.1% w/v LAP solution in DPBS to reach a final GelMA concentration of 5% w/v at 40°C. This homogeneous GelMA solution was poured into cylindrical acrylic molds (diameter = 10 mm, height = 1 mm). To prevent dehydration, molds were placed in a dark, custom‐built humidity chamber. After cooling at 4°C overnight for physical crosslinking, hydrogels underwent photocrosslinking under light (wavelength = 395‐400 nm, intensity = 15 mW cm^−2^) for 5 min, resulting in stable bulk hydrogel scaffolds.

### 4.23 Compression Test

The mechanical properties of GelMA bulk hydrogel scaffolds were assessed using an Instron mechanical tester (Model 5542, Instron Corporation, MA, USA). Samples were pre‐incubated at 37°C for 2 h, punched (diameter ∼8 mm and height ∼1 mm), and subjected to uniaxial compression at a controlled displacement rate of 1 mm min^−1^. Testing continued until ∼70% strain or sample failure. Stress‐strain curves were recorded, and the compressive modulus was calculated from the initial linear region, between 0 and ∼10% strain.

### 4.24 Rheological Assessments

Viscoelastic properties of GelMA hydrogel scaffolds and tissue‐like structures were examined using a rotational rheometer (AR‐G2, TA Instruments, DE, USA) with parallel plates (8 mm top plate and 20 mm bottom plate, both sandblasted stainless steel). Samples were punched to match the upper plate diameter for full coverage, followed by incubation in DBPS at 37°C for 2 h. Rheological tests (performed at 37°C) included oscillatory amplitude sweep at 0.1% to 100% strain and a constant frequency of 1 rad s^−1^ to identify the LVR and subsequent frequency sweep at 0.1% strain, from 0.1 to 100 rad s^−1^. The storage modulus (*G*′) and loss modulus (*G″*) were registered to assess the viscoelasticity of samples.

### 4.25 Agent‐Based Model (ABM)

We treat the experimental cell‐microgel system as a collection of soft self‐propelled active particles (fibroblast cells) and passive particles (microgels). Considering the far‐from‐equilibrium nature of the cell‐microgel system because of cell activeness, we build an ABM based on an existing model reported by Fily et al. [82] and others [83], which has successfully described rich non‐equilibrium phenomena, such as phase separation [84] and pattern formation [85]. As shown in Figure S34, both microgels and cells are modeled as elastic spheres with different radii, the motion of which resides on a rigid substrate. The ABM details are discussed below.

Fibroblast cells are modeled as self‐propelled spheroids with a diameter of ∼15 µm, and the positional and orientational degrees of freedom are described by the position vector ***r***_***i***_ = [*r*_*i*,*x*_, *r*_*i*,*y*_, *r*_*i*,*z*_]  and the polarity vector ${\hat{n}}_{i}=\left[{\hat{n}}_{i,x},{\hat{n}}_{i,y},{\hat{n}}_{i,z}\right]$, respectively. Similarly, microgels are passive elastic spheres with a diameter of ∼30, 80, and 150 µm for BHS‐S, BHS‐M, and BHS‐L, respectively. The cell culture substrate is modeled as an infinite elastic half plane. The boundary condition for the simulation box is periodic on the *x*‐*y* plane, and the simulation box height *H* is set as 1000 µm, a value high enough for all possible aggregate sizes.

#### 4.25.1 Elastic Repulsions

Since fibroblast cells and microgels are both modeled as elastic spheres, the elastic contact interaction should be considered. Here, we apply the JKR contact theory [45] to treat the passive interactions between the particles. The JKR theory is composed of two parts, which are elastic repulsion and adhesion. The elastic repulsion for the JKR theory is similar to the linear elastic Hertzian contact mechanics, where the repulsion is quantified by the overlapping distance between two objects. The elastic repulsive force on particle *i* exerted by particle *j* is written as

(4) $$F_{\mathrm{ij}}^{\mathrm{elastic}}=-\frac{5}{2}E_{\mathrm{ij}}R_{\mathrm{ij}}^{1/2}d_{\mathrm{ij}}^{3/2}{\hat{e}}_{\mathrm{ij}},$$

where *E_ij_* and *R_ij_* are equivalent contact modulus and contact radius of the particle pair *ij*, respectively, *d_ij_* is the overlapping distance between particles, and ${\hat{e}}_{\mathrm{ij}}$ is the unit vector, denoting the direction from the center of mass of particle *i* to particle *j*. The equivalent contact modulus *E_ij_* is given by

(5) $$\frac{1}{E_{\mathrm{ij}}}=\frac{1\;-\;\nu_{i}^{2}}{E_{i}}+\frac{1\;-\;\nu_{j}^{2}}{E_{j}},$$

with ν being the Poisson's ratio, and *E* the Young's modulus for agent types of *i* and *j*, respectively. The equivalent contact radius *R_ij_* is given by

(6) $$\frac{1}{R_{\mathrm{ij}}}=\frac{1}{R_{i}}+\frac{1}{R_{j}}.$$

The elastic repulsions between particles (cell‐cell, cell‐microgel, and microgel‐microgel), described in Equations (4)‐(6), capture the scaling law of contact elastic energy and its dependence on the Young's modulus and radius of different agent types [83].

#### 4.25.2 Inter‐Particle Adhesion

Due to the cadherin‐ and integrin‐mediated adhesion, live fibroblast cells can form adhesive junctions between cells or microgels. Here, we apply the Derjaguin approximation [86] to describe the adhesion interaction between particles, which yields

(7) $$F_{\mathrm{ij}}^{\mathrm{adh}}=\pi a_{\mathrm{ij}}^{2}\gamma_{\mathrm{ij}}{\hat{e}}_{\mathrm{ij}}$$

where *a_ij_* is the radius of the contact area, provided by the geometric relationship $a_{\mathrm{ij}}=\sqrt{d_{\mathrm{ij}}R_{\mathrm{ij}}}$ [87], and γ_*ij*_ is the particle‐particle adhesion energy density. We neglected the adhesion‐detachment asymmetry in the original JKR model, because the detachment event is rarely seen in the experiments. γ_*ij*_ takes the value of $\gamma_{\mathrm{ij}}^{\mathrm{cell}-\mathrm{cell}}$ and $\gamma_{\mathrm{ij}}^{\mathrm{cell}-\mathrm{gel}}$ for cell‐cell contact and cell‐gel adhesion, respectively.

#### 4.25.3 Particle‐Substrate Interactions

We assume the substrate as an elastic infinite plane, located on the plane *z* = 0. The elastic contact and adhesion between particles and substrate are also described by the simplified JKR model, where the elastic repulsion of particle *i* is given by

(8) $$F_{i}^{\mathrm{elastic}}=2E_{i}R_{i}^{1/2}d_{i}^{3/2}\hat{z}$$

where *E_i_* and *R_i_* are the elastic modulus and radius of particle *i*, respectively, *d_i_* is the overlapping distance between particle *i* and the substrate, and $\hat{z}$ is the unit vector on the *z*‐direction. Similar to Equation (7), the particle‐substrate adhesive force is described by

(9) $$F_{i}^{\mathrm{adh}}=-\pi a_{i}^{2}\gamma_{i}\hat{z}$$

with $a_{i}=\sqrt{d_{i}R_{i}}$ the radius of the contact area, and γ_*i*_ the particle‐substrate adhesion energy density. γ_*i*_ takes the value of $\gamma_{i}^{\mathrm{cell}-\mathrm{substrate}}$ for cell‐substrate adhesion, and zero for gel‐substrate adhesion.

#### 4.25.4 Active Self‐Propulsion

When fibroblast cells encounter a surface, their first step toward adhesion involves the formation of focal adhesions with the substrate through cell‐surface receptors, known as integrins, which bind to ECM proteins, such as fibronectin, collagen, and laminin [88]. Here for the microgel surface, RGD peptide motifs and fibronectin are two main components driving the cell‐microgel adhesion. This binding is not static; integrins cluster and couple to the actin cytoskeleton inside the cell through a variety of intracellular proteins, forming structures known as focal adhesions [89]. These focal adhesions serve as both structural anchors and as sites for signal transduction, relaying information about the external environment to the inside of cell, which affects cell behavior and function.

Following the adhesion mediated by integrins, fibroblast cells begin the process of crawling, which is primarily driven by the polymerization and depolymerization of actin filaments within the cell [90]. This actin dynamics generate protrusive and contractile forces. At the front of cells, actin polymerization pushes the membrane forward to form structures such as lamellipodia and filopodia, which explore the environment and help the cells anchor forward. Concurrently, myosin motor proteins power and interact with actin filaments to generate contractile forces at the rear of cells. These forces are transmitted through the focal adhesions to the substrate, pulling the cell body forward [91].

Considering the above complex bio‐chemo‐mechanical signaling process of fibroblast cell migration, we neglect the details of biochemical signaling pathways during the cell migration and simplify the cellular motion as a directed active self‐propulsion, mediated by phenomenological random noises. Note that cell migration depends on the formation of focal adhesions, thus a binding surface is essential to any possible contractile forces driving the cellular motions. This is inherently different from the swimming of bacteria in fluids, which is governed by the motion of flagella. Therefore, although the theories of active Brownian particles have been successfully applied to the bacteria suspension systems [92] and 2D cellular migrations [46], special treatment needs to be taken when extending the theory to the 3D cellular migrations.

Here, we define the cellular active force as

(10) $$F_{i,a}=F_{a}{\hat{n}}_{i}^{∥},$$

where *F*_a_ is the magnitude of the cellular self‐propulsion force, and ${\hat{n}}_{i}^{∥}$ is the projected cell polarity vector [46] on a binding surface, given by

(11) $${\hat{n}}_{i}^{∥}={\hat{n}}_{i}-\left({\hat{n}}_{i}\;\times \;{\hat{n}}_{i}^{\mathrm{surface}}\right){\hat{n}}_{i}^{\mathrm{surface}}$$

where ${\hat{n}}_{i}^{\mathrm{surface}}$ is the unit outer norm vector of binding surface. Note that the active propulsion force ***F***_*i*,*a*_ obeys Newton's third law, which means the particle providing the binding surface also sustains a counter‐acting force with the same magnitude in the opposite direction.

To address the biochemical randomness and fluctuations during the cellular motion, we introduce two randomized forces acting on the center of mass and the polarity vector of cell particles. The cell positional and orientational fluctuations [46] are described by the Gaussian noise terms ***f***_*r*,*i*_(*t*) and ***f***_θ,*j*_(*t*), respectively, with a zero mean value and the spatiotemporal correlations

(12) $$\frac{1}{k}\sum_{k}\sqrt{f_{r,i}^{k}\left(t\right)f_{r,j}^{k}\left(t^{\prime }\right)}=f_{r}\delta_{\mathrm{ij}}\delta \left(t\;-\;t^{\prime }\right),$$

(13) $$\frac{1}{k}\sum_{k}\sqrt{f_{\theta ,i}^{k}\left(t\right)f_{\theta ,j}^{k}\left(t^{\prime }\right)}=f_{\theta }\delta_{\mathrm{ij}}\delta \left(t\;-\;t^{\prime }\right),$$

where $\frac{1}{k}\sum_{k}\sqrt{\cdot }$ is the ensemble average, and *f_r_* and *f*_θ_ are the magnitudes of translational and rotational fluctuations, respectively. *δ*(*t*) is the delta function of time, and ***δ**_ij_* = [1,  1,  1]  if *i* = *j*, otherwise ***δ**_ij_* = [0,  0,  0] .

#### 4.25.5 Dissipation

We consider three sources of dissipation. First, when fibroblast cells or microgels move along the elastic substrate, static or dynamic friction forces dissipate kinetic energy. Second, when fibroblast cells crawl on the surface of microgels, a dynamic friction exists. Third, the surrounding cell culture fluid exerts resistance opposite to the motion of cells or microgels. Here, we discuss the dissipation.

The friction between the particles and substrate may be given by a linear simplification of dynamic friction forces. The particle‐substrate friction force is given by

(14) $$F_{i,s}=-\eta_{i}^{\mathrm{substrate}}v_{i}^{∥}$$

where η_*i*_ is the friction coefficient between the particle *i* and the substrate. The velocity parallel to the surface is $v_{i}^{∥}=v_{i}-\left(v_{i}\cdot \hat{z}\right)\hat{z}$, where ***v**_i_* is the velocity of particle *i*, and $\hat{z}$ is the unit vector pointing to the *z*‐direction.

Similarly, the dynamic friction between cells and microgels is given by

(15) $$F_{\mathrm{ij}}^{\mathrm{surface}}=-\eta_{\mathrm{ij}}^{\mathrm{surface}}v_{\mathrm{ij}}^{∥}$$

where $\eta_{\mathrm{ij}}^{\mathrm{surface}}$ is the friction coefficient between the particles *i* and *j*, and $v_{\mathrm{ij}}^{∥}$ is the projection of the relative velocity between the particles *i* and *j*.

#### 4.25.6 Choice of Parameters

We use the mean squared displacement (MSD) measurement of cell migrations to calibrate the magnitude of cellular self‐propulsion force *F*_a_  =  *v*_a_ η_cell_, where η_cell_  =  2  ×  10^−3^ Pa m s is the friction coefficient of cells [53], originating from culture medium viscosity, and *v*_a_ is cell migration speed. To experimentally estimate *v*_a_, we use the *trackpy* package [76] to track the cell trajectory during the 72 h culture time, and calculate the time‐dependent MSD(Δ*t*), where Δ*t* is the time window. The results are then fitted by the theoretical model of active Brownian particles [47]. Neglecting the translational fluctuation *f_r_*, $\mathrm{MSD}\;\left(\Delta t\right)=2v_{a}^{2}\tau_{\theta }\Delta t+2v_{a}^{2}\tau_{\theta }^{2}\left[e^{-\Delta t/\tau_{\theta }}-1\right]$, and $\tau_{\theta }=\eta_{\mathrm{cell}}R_{i}^{2}/f_{\theta }$ is the characteristic time for rotational diffusion. Nonlinear regression yields $v_{a}=1.6\times 10^{-2}\;\mu m\;s^{-1}$ and *τ*_θ_  =  833 s. We find that the fitting results of *τ*_θ_ is consistent with the reported measurements on fibroblast cells [48, 49]$\;\tau_{\theta }∼1000\;s$, but *v*_a_ is about one order of magnitude smaller than the reported value $v_{a}∼0.2\;\mu m\;s^{-1}$ [48, 49]. In simulations, we take $v_{a}=1.9\times 10^{-2}\;\mu m\;s^{-1}$ to maximally reproduce the experimentally observed kinetics. The Young's modulus of living fibroblast cells is taken as *E*_c_ = 5 kPa [50], and the Young's modulus of microgels *E*_gel_  =  70 kPa (Figure S35) for all three sizes. Poisson's ratio is taken as ν=0.45 for simplicity [54]. The equivalent contact modulus *E*_ij_ (inter‐particles) and *E*_i_ (particle‐substrate) are calculated using Equation (5), where the Young's modulus of the rigid substrate is taken as infinite. We choose the cell‐cell adhesion energy density as $\gamma_{\mathrm{ij}}^{\mathrm{cell}-\mathrm{cell}}=1\;\times \;10^{-5}\;N\;m^{-1}$ [85] to maximally reproduce the experimentally measured aggregation kinetics, following the reported value of 0.01‐0.5 mJ m^−2^ [51, 52]. Cell–gel adhesion energy density is assumed to be $\gamma_{\mathrm{ij}}^{\mathrm{cell}-\mathrm{gel}}=3\;\times \;10^{-5}\;N\;\;m^{-1}$, and cell‐substrate adhesion energy density is assumed as $\gamma_{i}^{\mathrm{cell}-\mathrm{substrate}}=2\;\times \;10^{-6}\;N\;m^{-1}$. Inter‐particle friction coefficient is taken as $\eta_{\mathrm{ij}}^{\mathrm{surface}}=1\;\times \;10^{3}\;\mathrm{Pa}\;m\;s$, and particle‐substrate friction is taken as $\eta_{i}^{\mathrm{substrate}}=2\;\times \;10^{4}\;\mathrm{Pa}\;m\;s$.

#### 4.25.7 Implementation

We implemented the above framework of ABM into the open‐source LAMMPS molecular dynamics simulator, as a customized module. The code has been uploaded to Zenodo (https://doi.org/10.5281/zenodo.18318891). Other data are available upon reasonable request.

### 4.26 Estimation of Effective Diffusion Coefficient

To compute the effective diffusion coefficient of a BHS, which is composed of passive microgels and active cells, we consider the entire aggregate as an active Brownian particle (neglecting the shape change), which exhibits ballistic motion within relatively short time scales and diffusive motion at longer time scales. As the size of BHS grows, cells gradually attach to the aggregate, and the net active driving force evolves with time. Here, we fit the effective diffusion coefficient in the diffusive time scale, where the sampling time window Δ*t* > τ_θ_. In this regime, the in‐plane MSD follows the linear relation $\left\langle \Delta r_{∥}^{2}\right\rangle ∼4D_{\mathrm{eff}}\Delta t$, where *D*_eff_ is the effective diffusion coefficient (fitting parameter), and Δ*t* is the time window. We set the sampling window as Δ*t* = 3 h, and the fitting data include 5000 different trajectories from 50 independent simulations.

### 4.27 Statistical Analyses

Experiments were conducted with at least three replicates. Data points in all figures represent independent replicates, except for Figures 1B, S5B, S9, S30B, and S30C, where all technical replicates within ≥3 independent repeats are shown to represent the data distribution. Normally distributed data were analyzed using *t*‐test, or either ordinary or repeated measures (RM) one‐way/two‐way analysis of variance (ANOVA), with significance determined by Tukey's post‐hoc multiple comparison test. Non‐normally distributed data were assessed using the Kruskal‐Wallis test, followed by Dunn's post‐hoc multiple comparison test. All statistical analyses were performed using GraphPad Prism (version 9.5.0). Groups with *p*‐value below 0.05 were considered significantly different, indicated by *p < 0.05, **p < 0.01, ***p < 0.001, and ****p < 0.0001.

## Author Contributions

A.S. conceptualized and conceived the project. Z.A. and A.S. experimentally developed the project. Z.A. and A.S. designed the experimental framework, and Z.A., A.R., and S.K. conducted the experiments. S.Z. and C.L. conducted the simulation and theoretical data interpretation and analysis. Z.A., C.L., A.R., S.K., and A.S. conducted experimental data interpretation and analysis. As.Se. and I.A. conducted RNA‐seq data interpretation and analysis. Z.A., S.K., C.L., As.Se, I.A., S.Z., and A.S. wrote the manuscript. All co‐authors edited the manuscript.

## Funding

Research reported in this publication was partially supported by the National Heart, Lung, and Blood Institute of the National Institutes of Health under Award Number R01HL167939 (A.S.). The content is solely the responsibility of the authors and does not necessarily represent the official views of the National Institutes of Health. We also acknowledge the support from the Vice Provost and Dean of the Graduate School Student Persistence Scholarship (S.K.), Max M. Snyder Graduate Scholarship in Engineering (S.K.), College of Engineering Diefenderfer Graduate Fellowship in Entrepreneurship (S.K.), the Meghan Rose Bradley Foundation (A.S.), Dorothy Foehr Huck and J. Lloyd Huck Early Career Chair (A.S.), the Materials Research Institute (MRI) 2022 seed grant for the Convergent Research at the Intersection of Materials‐Life‐Health‐Environment (A.S.), and the College of Engineering Materials Matter at the Human Level seed grants (A.S.).

## Conflicts of Interest

The authors declare no conflicts of interest.

## Supporting information




**Supporting File**: advs74576‐sup‐0001‐SuppMat.pdf.

**Supplemental Video 1**: advs74576‐sup‐0002‐VideoS1.mp4.

**Supplemental Video 2**: advs74576‐sup‐0003‐VideoS2.mp4.

**Supplemental Video 3**: advs74576‐sup‐0004‐VideoS3.mp4.

**Supplemental Video 4**: advs74576‐sup‐0005‐VideoS4.mp4.

**Supplemental Video 5**: advs74576‐sup‐0006‐VideoS5.mp4.

**Supplemental Video 6**: advs74576‐sup‐0007‐VideoS6.mp4.

**Supplemental Video 7**: advs74576‐sup‐0008‐VideoS7.mp4.

**Supplemental Video 8**: advs74576‐sup‐0009‐VideoS8.mp4.

**Supplemental Video 9**: advs74576‐sup‐0010‐VideoS9.mp4.

**Supplemental Video 10**: advs74576‐sup‐0011‐VideoS10.mp4.

**Supplemental Video 11**: advs74576‐sup‐0012‐VideoS11.mp4.

**Supplemental Video 12**: advs74576‐sup‐0013‐VideoS12.mp4.

## Data Availability

The data that support the findings of this study are available from the corresponding author upon reasonable request.
