# Supplementary material for: Cellular Snowballing: Cell Adhesion and Migration Drive the Self‐Assembly of Cell‐Microgel Biohybrid Spheroids
Source: Adv Sci (Weinh). 2026 Mar 17;13(30):e11302. doi: 10.1002/advs.202511302 (PMC13248798; doi:10.1002/advs.202511302)
Supplement: Supplementary file 1 — Supporting File: advs74576‐sup‐0001‐SuppMat.pdf. [file ADVS-13-e11302-s011.pdf]

Supporting Information for

Cellular Snowballing: Cell Adhesion and Migration Drive the Self-Assembly of Cell-Microgel Biohybrid Spheroids

Zaman Ataie,[1] Sina Kheirabadi,[1] Changhao Li,[2] Aneesh Risbud,[3] Aswathy Sebastian,[4] Istvan Albert,[5] Sulin Zhang,[2,3,6] Amir Sheikhi[1,3,4,7,8*]

[1]Department of Chemical Engineering, The Pennsylvania State University, University Park, PA 16802, USA

[2]Department of Engineering Science and Mechanics, The Pennsylvania State University, University Park, PA 16802, USA

[3]Department of Biomedical Engineering, The Pennsylvania State University, University Park, PA 16802, USA

[4]Huck Institutes of the Life Sciences, The Pennsylvania State University, University Park, PA 16802, USA

[5]Department of Biochemistry and Molecular Biology, The Pennsylvania State University, University Park, PA 16802, USA

[6]Department of Materials Science and Engineering, The Pennsylvania State University, University Park, PA 16802, USA

[7]Department of Chemistry, The Pennsylvania State University, University Park, PA 16802, USA

[8]Department of Neurosurgery, College of Medicine, The Pennsylvania State University, Hershey, PA 17033, USA

*Corresponding Author: Amir Sheikhi (sheikhi@psu.edu)

The Supporting Information includes:

Figures S1 to S35

Table S1

References 1 to 19

Other Supporting Information for this manuscript includes the following:

Videos S1 to S12

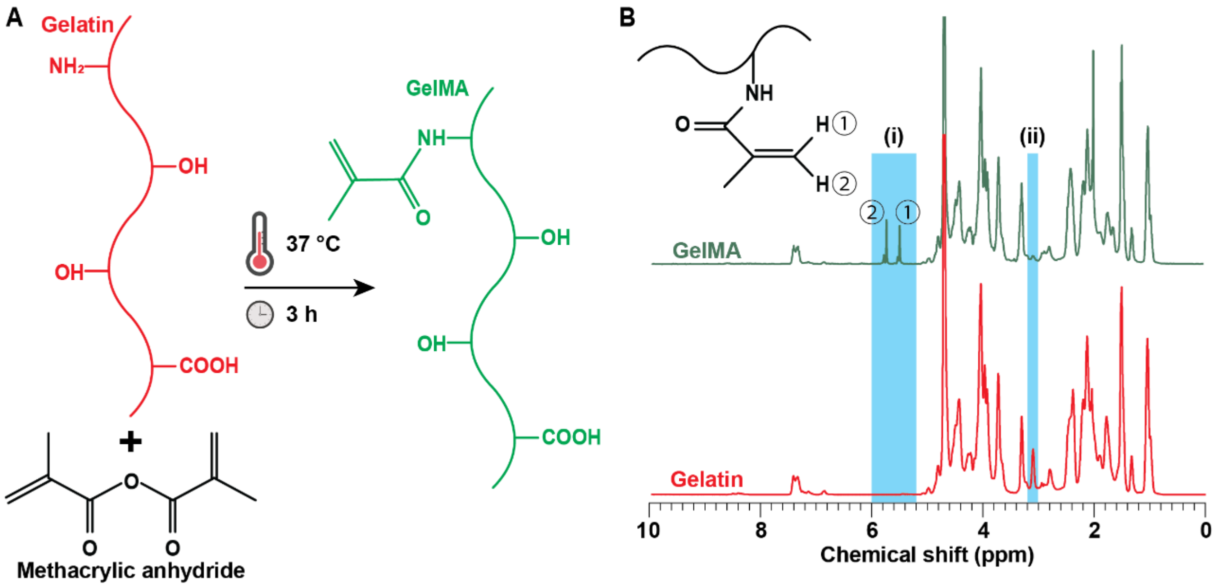

Figure S1. GelMA synthesis. (A) GelMA synthesis through the reaction of gelatin with MAA. **(B)** ^{1}H NMR spectra of GelMA and gelatin: (i) vinyl group peaks in GelMA, confirming the successful MA-modification of gelatin biopolymer, and (ii) a reduction in the lysine proton peak area, reflecting the chemical substitution.

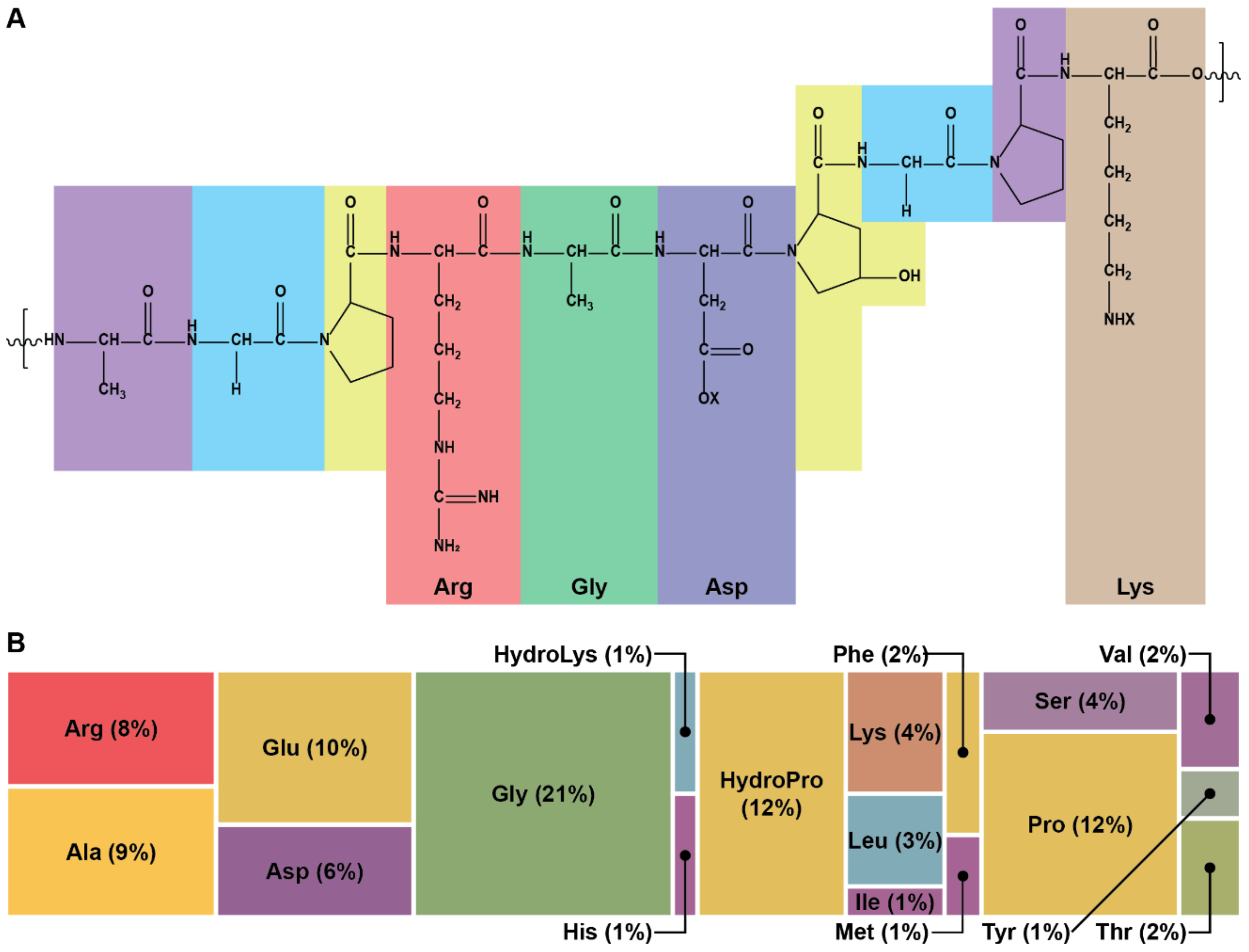

Figure S2. GelMA chemical structure. (A) Chemical structure of GelMA, containing the RGD peptide motifs, where X represents either a hydrogen or an MA group as a result of MA modification. **(B)** Typical amino acid composition of gelatin[1].

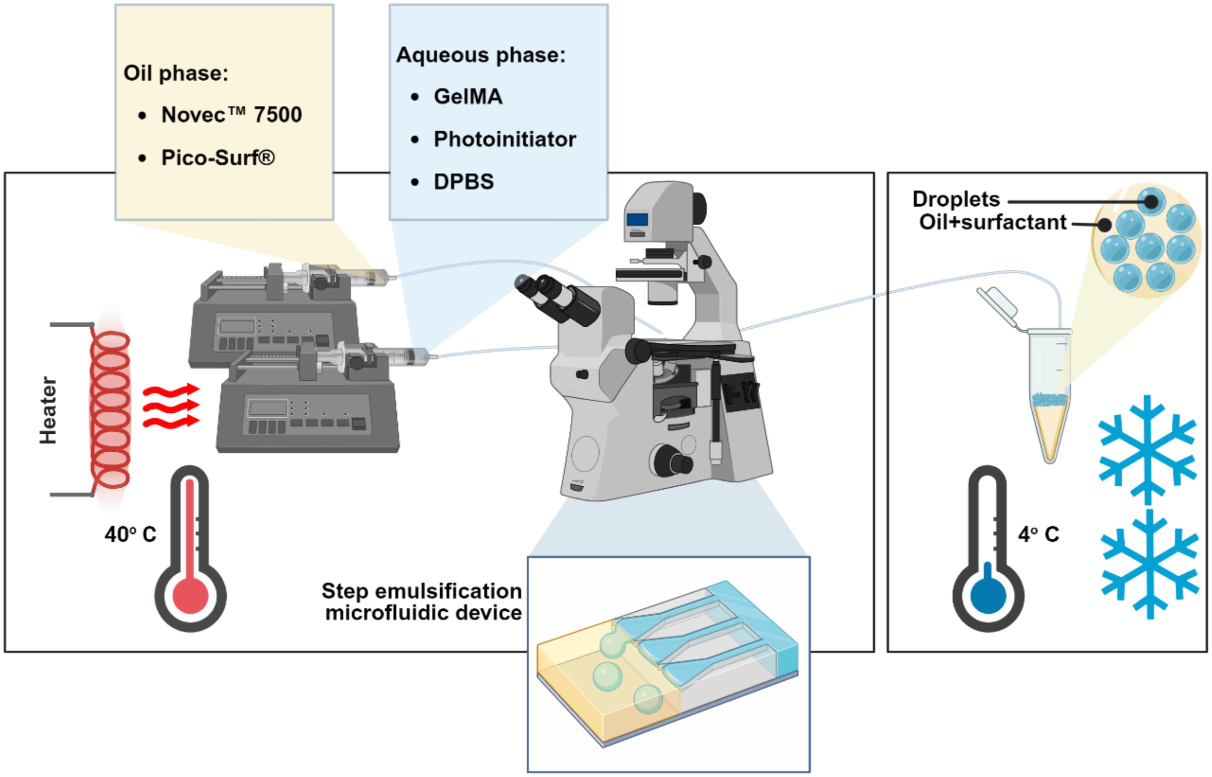

Figure S3. Experimental setup for GelMA droplet fabrication using a step emulsification microfluidic device. A GelMA solution (5% w/v in DPBS, containing 0.1% w/v of LAP as the photoinitiator) serves as the aqueous phase, while an engineering fluid mixed with a biocompatible surfactant is used as the oil phase in the microfluidic device inlets. The droplet formation is observed under a microscope while maintaining the temperature at ~ 40 °C. Droplets are then incubated at ~ 4 °C overnight to form physically crosslinked GelMA microgels.

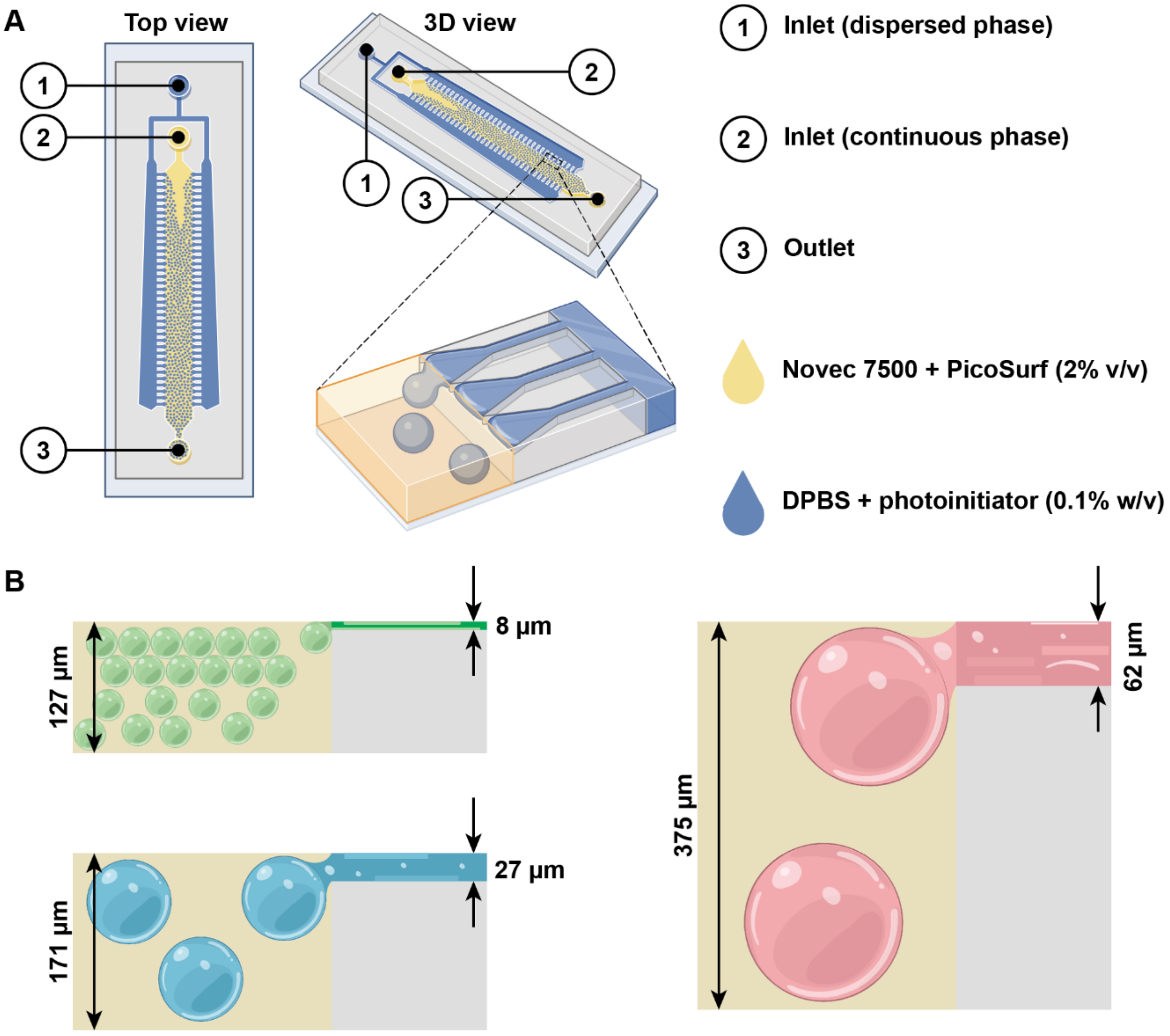

Figure S4. Step emulsification microfluidic devices. (A) Schematic illustration of the step-emulsification microfluidic device, representing the inlets and outlet, alongside the aqueous (dispersed) and oil (continuous) phases. **(B)** In step emulsification devices, the droplet size is correlated with the step size. Increasing the step size results in larger droplets, enabling the fabrication of three distinct droplet sizes.

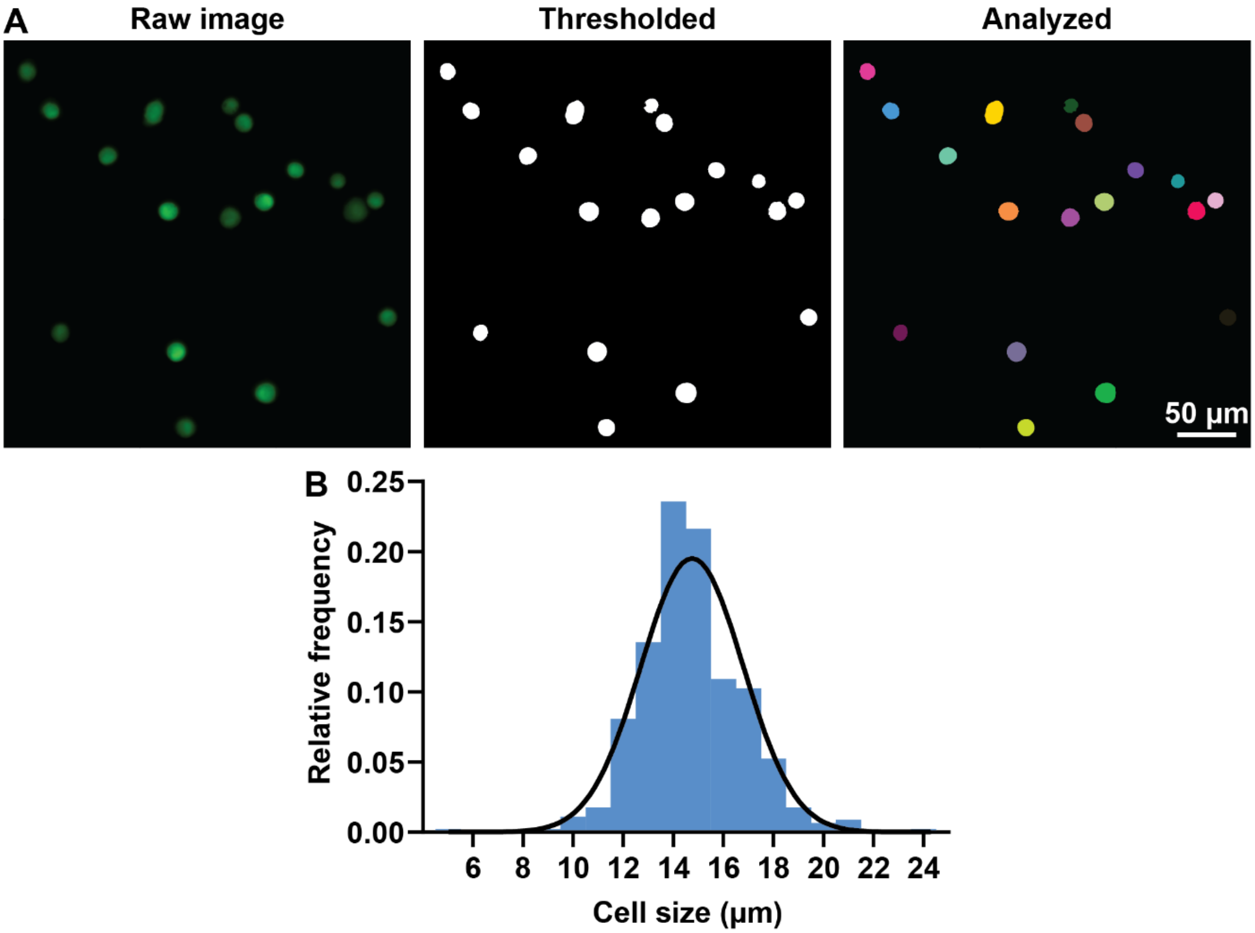

Figure S5. NIH/3T3 fibroblast cell size distribution. (A) Raw fluorescence microscopy images of suspended NIH/3T3 murine fibroblast cells, labeled with CellTracker Green CMFDA, are processed to measure cell area, followed by conversion to equivalent diameter. **(B)** Cell size distribution, showing an average of ~ 15 ± 2 μm for non-elongated cells (*n* ~ 100 cells).

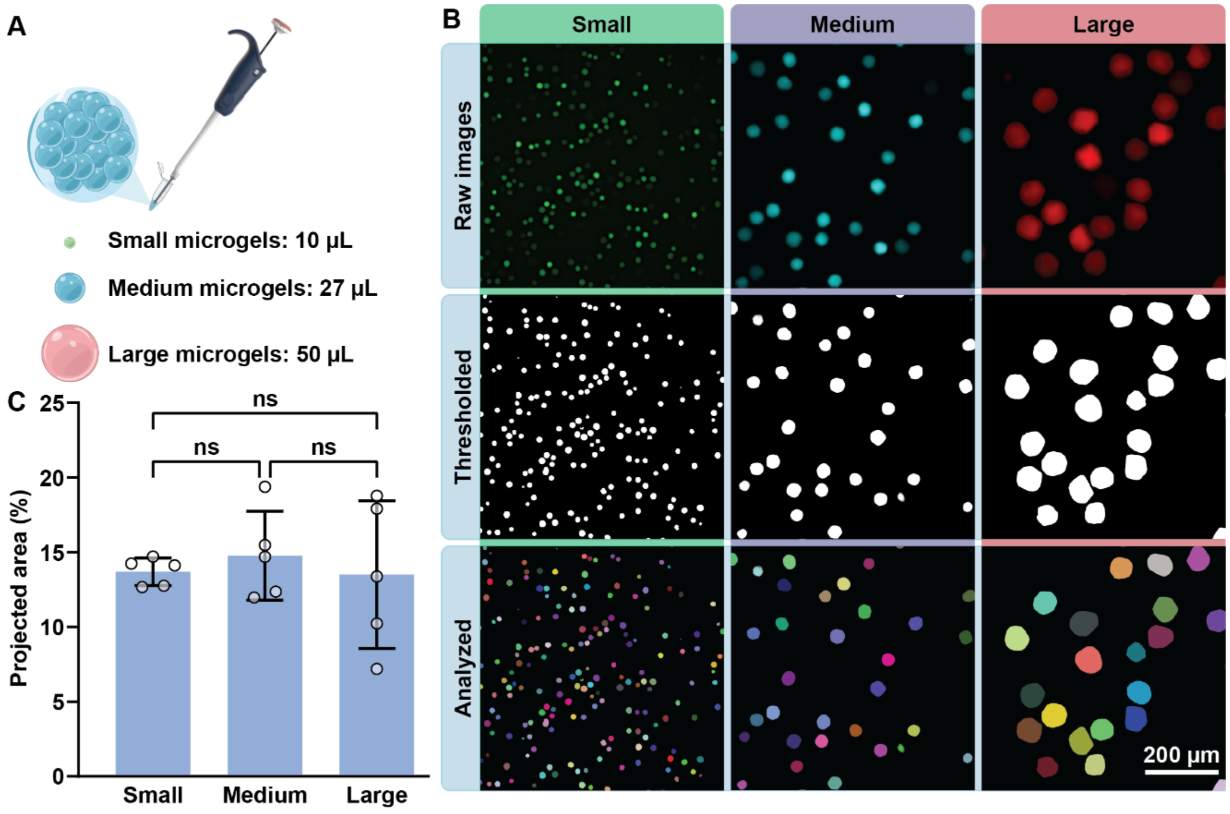

Figure S6. Total projected area of microgels. (A) Packed microgel suspensions (10, 27, and 50 μL of small, medium, and large microgels, respectively, packed at 3000 ×***g*** for 15 s) are transferred to 5 mL of DPBS using a positive displacement pipette. **(B)** Fluorescence microscopy images of the microgels, followed by thresholding and measuring the total projected area of microgels. **(C)** The projected area (%) is not significantly different among varying microgel sizes. Images are acquired from 5 different samples per microgel group, each with 3 technical replicates (three spots of the Petri dish). Ordinary one-way ANOVA, followed by Tukey's post-hoc multiple comparison test is performed (ns = not significant with $p \geq 0.05$).

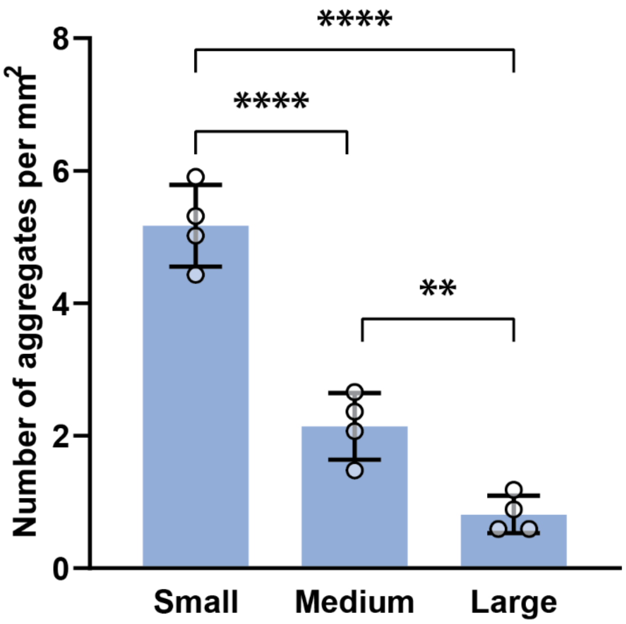

Figure S7. Number of aggregates versus microgel size. Number of aggregates formed via cell-mediated microgel assembly in a geometrically unconstrained environment (planar, non-treated substrate) per area using three different sizes of microgels (n = 4). Ordinary one-way ANOVA, followed by Tukey's post-hoc multiple comparison test are performed (**p<0.01, ****p<0.0001).

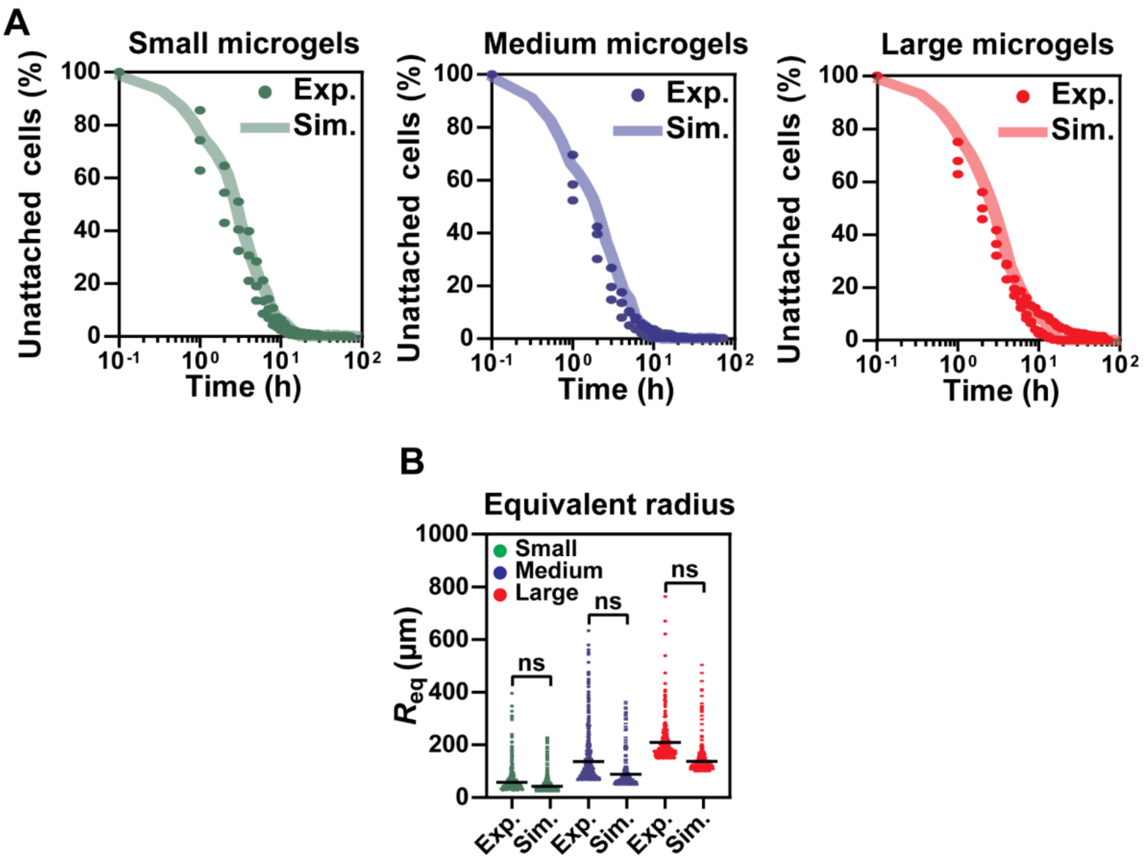

Figure S8. ABM and experimental results of BHS formation kinetics. (A) Time evolution of unattached cells, remaining non-adhered to small (left), medium (middle), or large (right) microgels. **(B)** Comparison of the distribution of stable $\boldsymbol{R_{eq}}$ (after 72 h in experiments and the model) in a 1000 µm × 1000 µm rectangular box for varying microgel sizes. The Kruskal-Wallis test, followed by Dunn's post-hoc multiple comparison test are performed (ns = not significant with $p \geq 0.05$, all other comparisons, not shown here: ****$p<0.0001$).

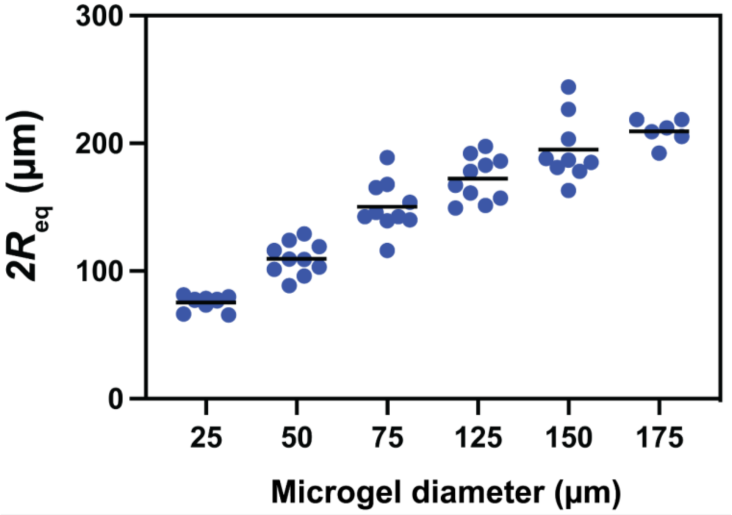

Figure S9. Simulated relationship between average stable aggregate size and microgel diameter. The simulation duration is 72 h, and each data point is an average of 10 different simulations with the same parameters.

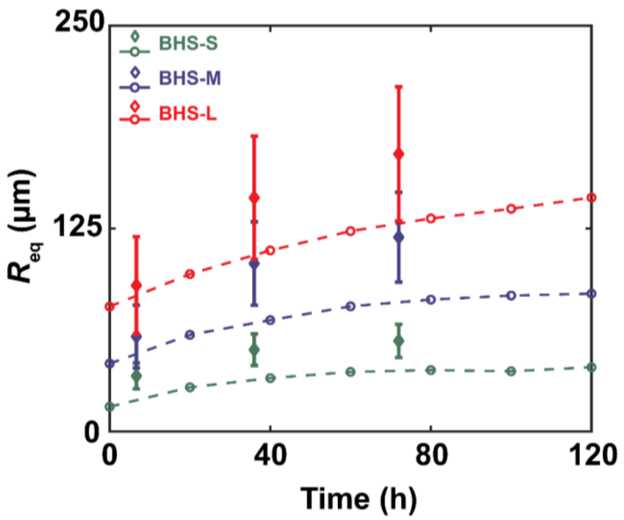

Figure S10. Size growth kinetics of BHS. The time evolution of equivalent radius for BHS-S, BHS-M, and BHS-L is compared between experiments (diamond with error bars) and ABM (circles connected by dashed lines). For each microgel size, experimental and simulation data are collected in a 1000 μm × 1000 μm rectangular box up to 120 h, with the same seeding/number density of cells and microgels.

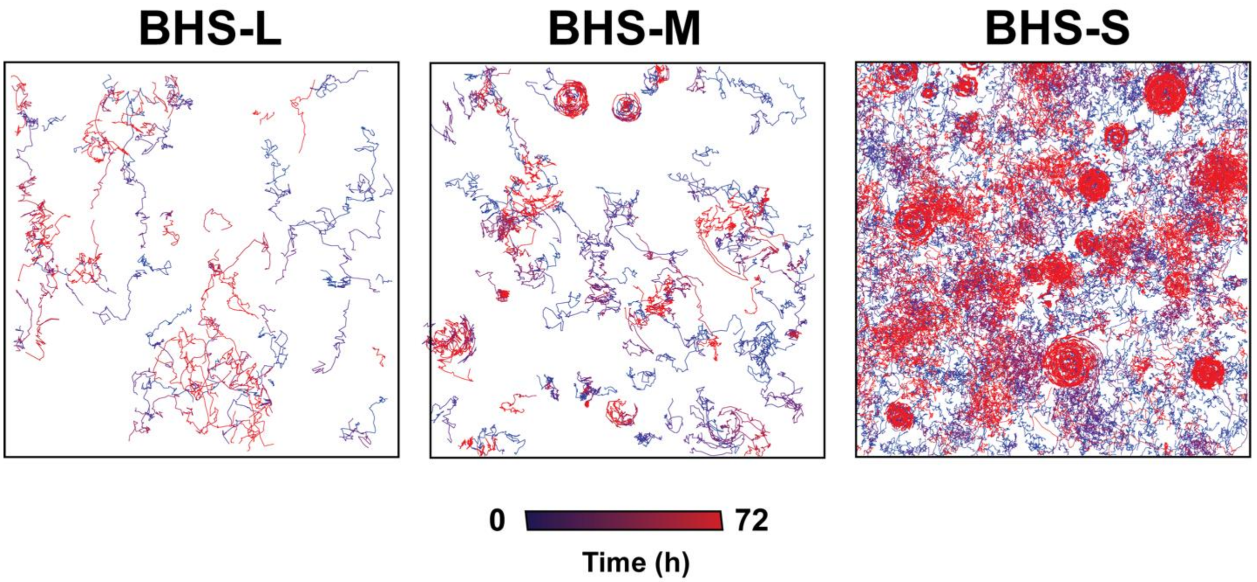

Figure S11. Experimentally measured microgel trajectories during BHS formation in a geometrically unconstrained environment for varying microgel sizes. The trajectories are color-coded by the elapsed time.

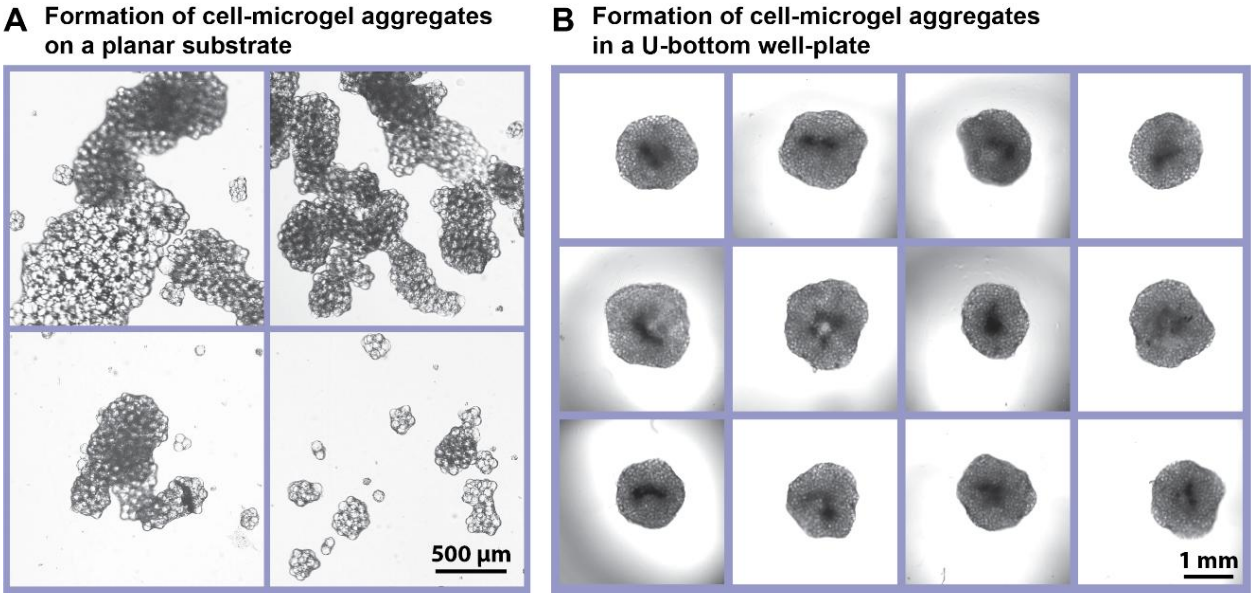

Figure S12. Effect of geometric constraint on BHS formation. BHS formation **(A)** in a geometrically unconstrained environment and **(B)** in a geometrically constrained environment (U-bottom low-attachment 96-well plate).

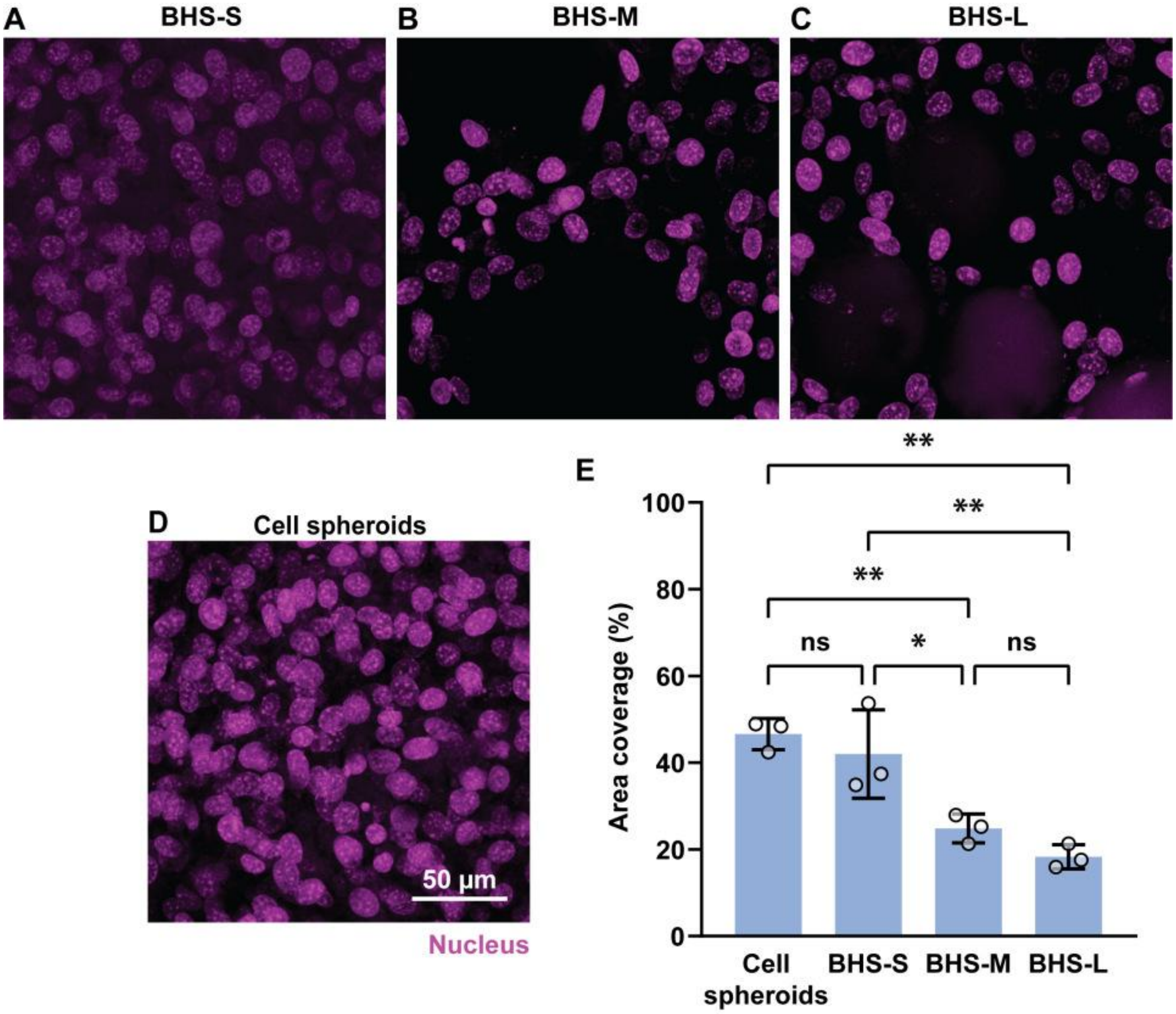

Figure S13. Nucleus density in BHS and cell spheroids. Samples are prepared in a geometrically constrained environment, as described in the "BHS and Cell Spheroid Formation" section of manuscript, for 3 days, then stained with Hoechst 33342. Confocal images of cell nuclei are acquired from **(A)** BHS-S, **(B)** BHS-M, **(C)** BHS-L, and **(D)** cell spheroids. **(E)** Nucleus area coverage of each study group (n = 3). Ordinary one-way ANOVA, followed by Tukey's post-hoc multiple comparison test are performed (ns = not significant with $p \geq 0.05$, *$p<0.05$, and **$p<0.01$).

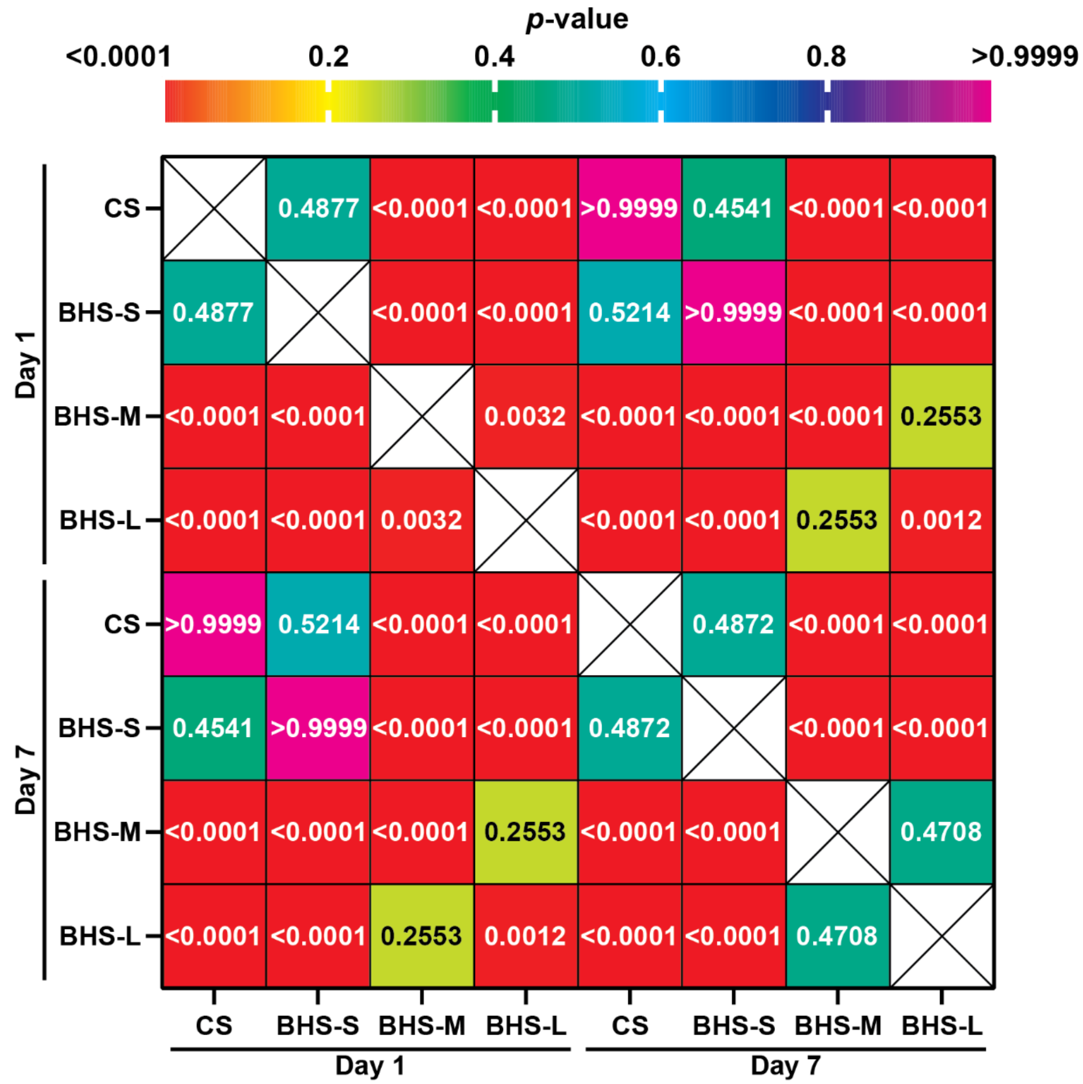

Figure S14. Heat map and *p*-value lookup table regarding the Tukey's post-hoc multiple comparison test after ordinary two-way ANOVA, corresponding to Figure 4F. CS denotes cell spheroids.

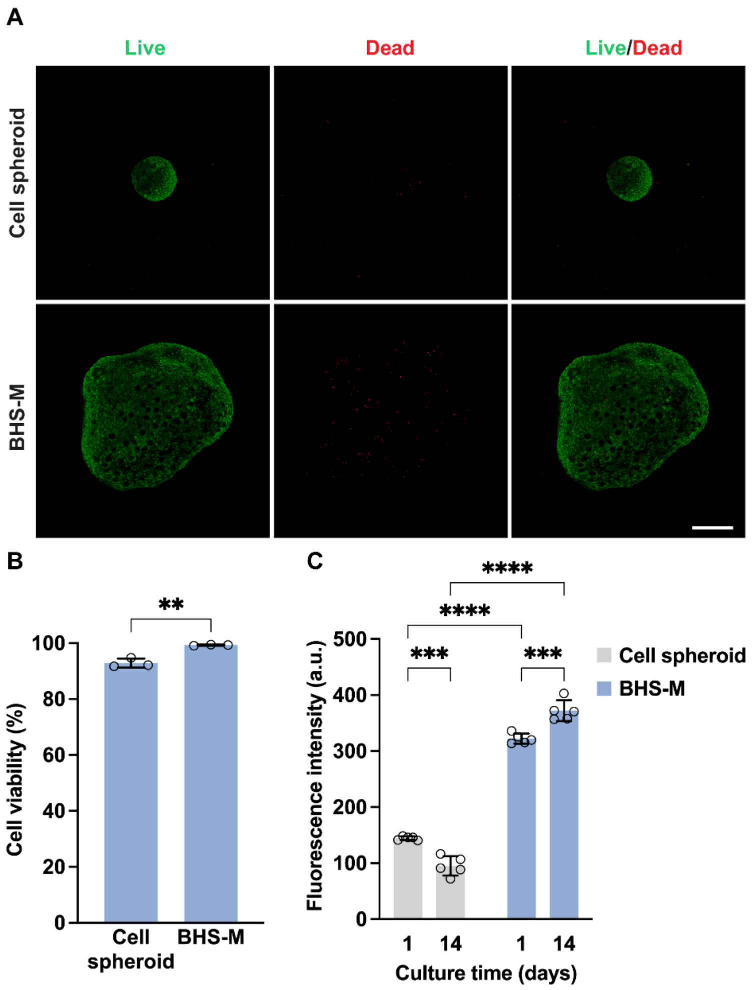

Figure S15. Cell viability and metabolic activity assessments over an extended culture period. **(A)** Fluorescence images of NIH/3T3 fibroblast cell spheroids and BHS-M, showing live (green) and dead (red) cells on day 14. **(B)** Cell viability in BHS-M is >99%, which is significantly higher than the ~93% viability observed in the cell spheroids (*n* = 3). Unpaired two-tailed *t*-test is performed (***p*<0.01). **(C)** Metabolic activity, measured using the PrestoBlue assay. The results indicate increased metabolic activity in BHS-M, whereas cell spheroids undergo a reduction (*n* = 5). All samples are formed as described in the "BHS and Spheroid Formation" section of manuscript and are cultured in a geometrically constrained environment. Two-way ANOVA, followed by Tukey's post-hoc multiple comparison test are performed (****p*<0.001 and *****p*<0.0001).

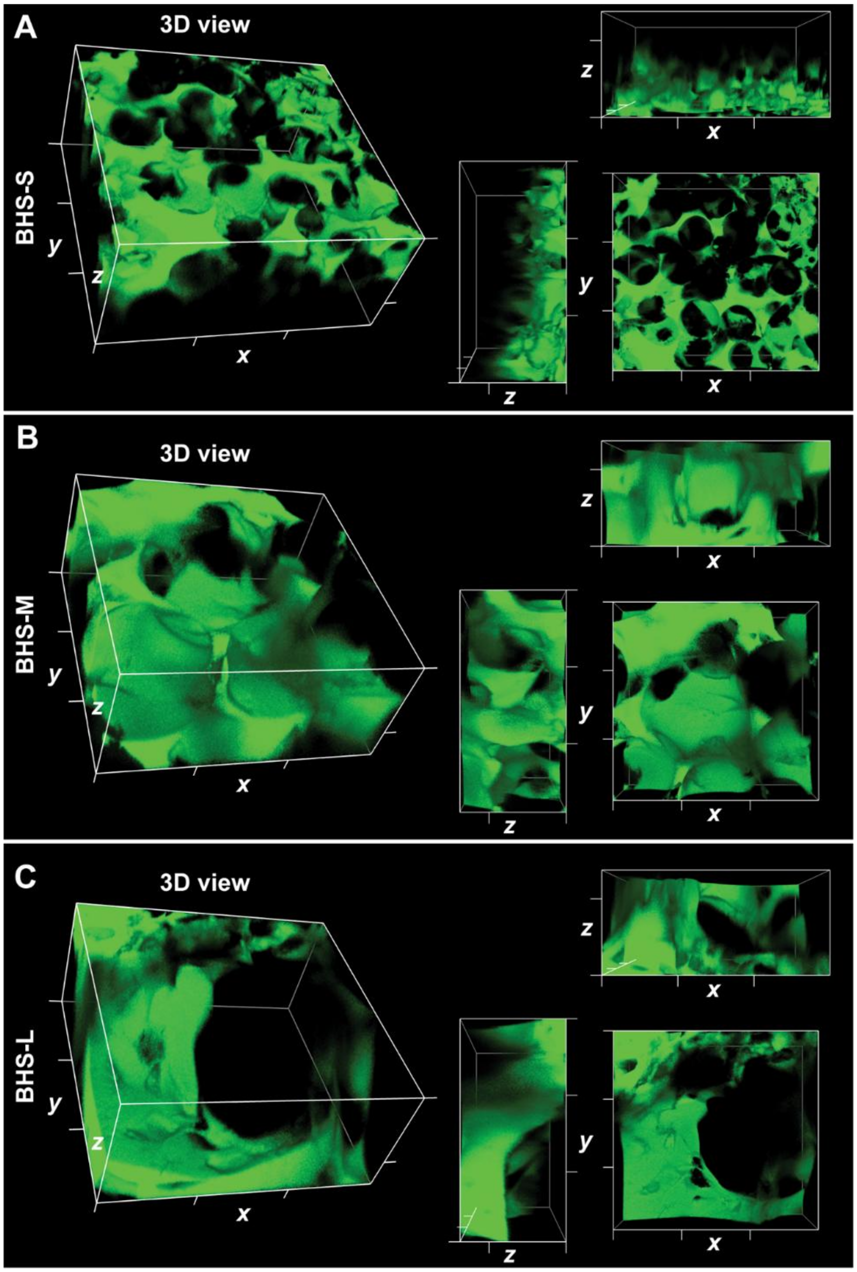

Figure S16. Analysis of void spaces in BHS. A high molecular weight FITC-dextran is used to fill the BHS interstitial void spaces, followed by confocal microscopy. Orthogonal (3D), side, and top views of void spaces in **(A)** BHS-S, **(B)** BHS-M, and **(C)** BHS-L. The volume of interest is ~ 150 × 150 × 68 μm^3 ($x \times y \times z$). Increments are 50 µm. All BHS are formed as described in the "BHS and Cell Spheroid Formation" section of manuscript, cultured in a geometrically constrained environment, and imaged on day 3.

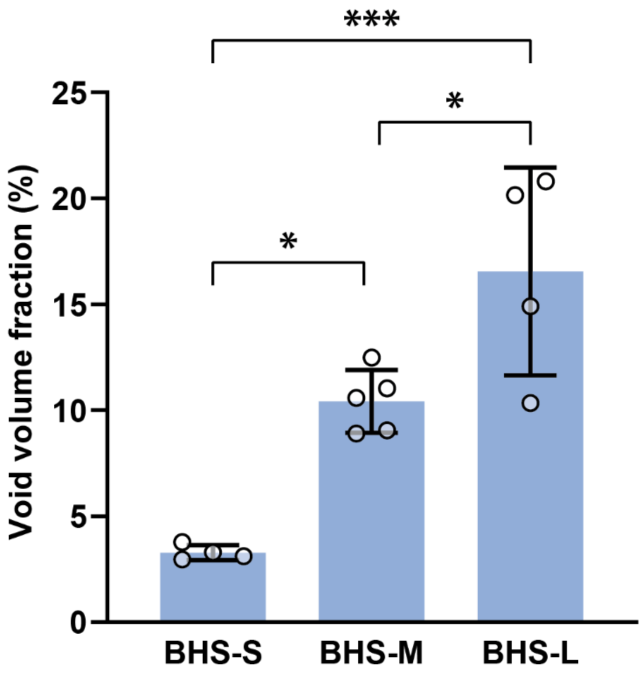

Figure S17. The void fraction of BHS-S, BHS-M, and BHS-L. For BHS-S and BHS-L, $n = 4$, and for BHS-M, $n = 5$. All BHS are formed as described in the "BHS and Cell Spheroid Formation" section of manuscript and are cultured in a geometrically constrained environment, with the void fraction measured on day 3. Ordinary one-way ANOVA, followed by Tukey's post-hoc multiple comparison test are performed (*$p<0.05$ and ***$p<0.001$).

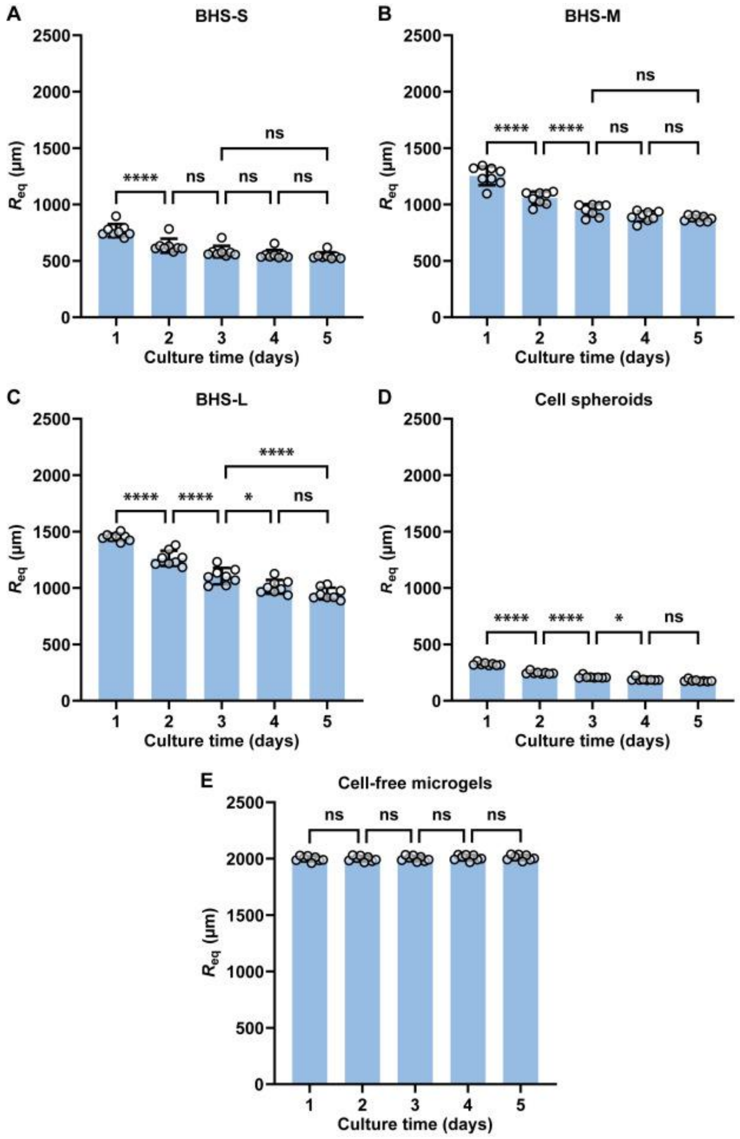

Figure S18. Size evolution of aggregates, formed in a geometrically constrained environment over time. Panels show the time evolution of aggregate equivalent radius (R_{eq}). **(A)** BHS-S reach size stability in 2 days, **(B)** BHS-M size is stabilized in 3 days, **(C)** BHS-L require 4 days for size stability, **(D)** cell spheroids stabilize after 4 days, and **(E)** the control group (cell-free microgels) does not undergo size change (n = 8). All BHS are formed as described in the "BHS and Cell Spheroid Formation" section of manuscript. One-way RM ANOVA, followed by Tukey's post-hoc multiple comparison test are performed (ns = not significant with $p \geq 0.05$, *$p < 0.05$, and ****$p < 0.0001$).

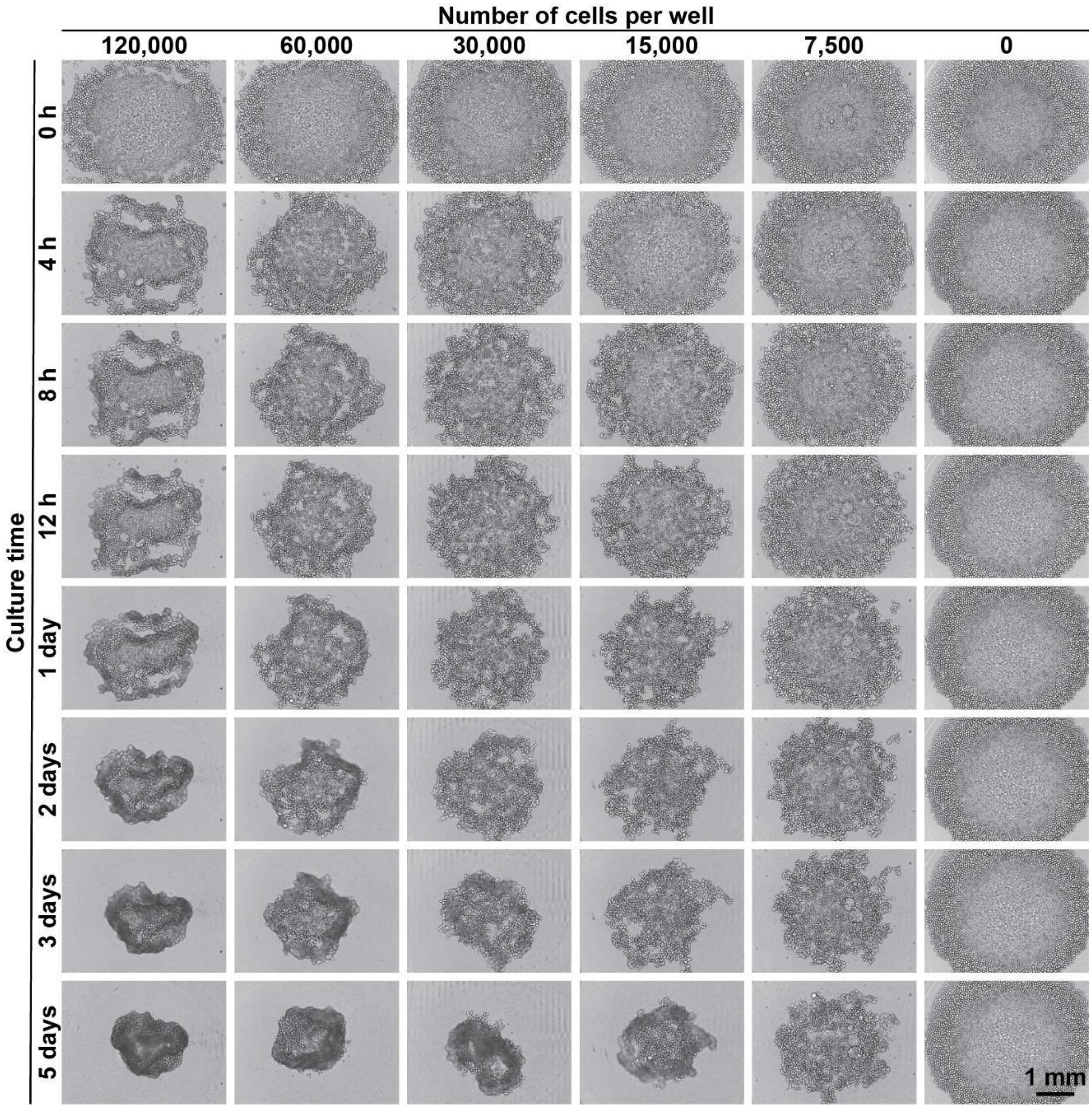

Figure S19. Brightfield images of BHS-M formation in a geometrically constrained environment at varying cell seeding densities over 5 days. Microgel amount is constant (1.08 µL of packed medium microgels, centrifuged at 3,000 ×***g*** for 15 s) in each well (96-well plate).

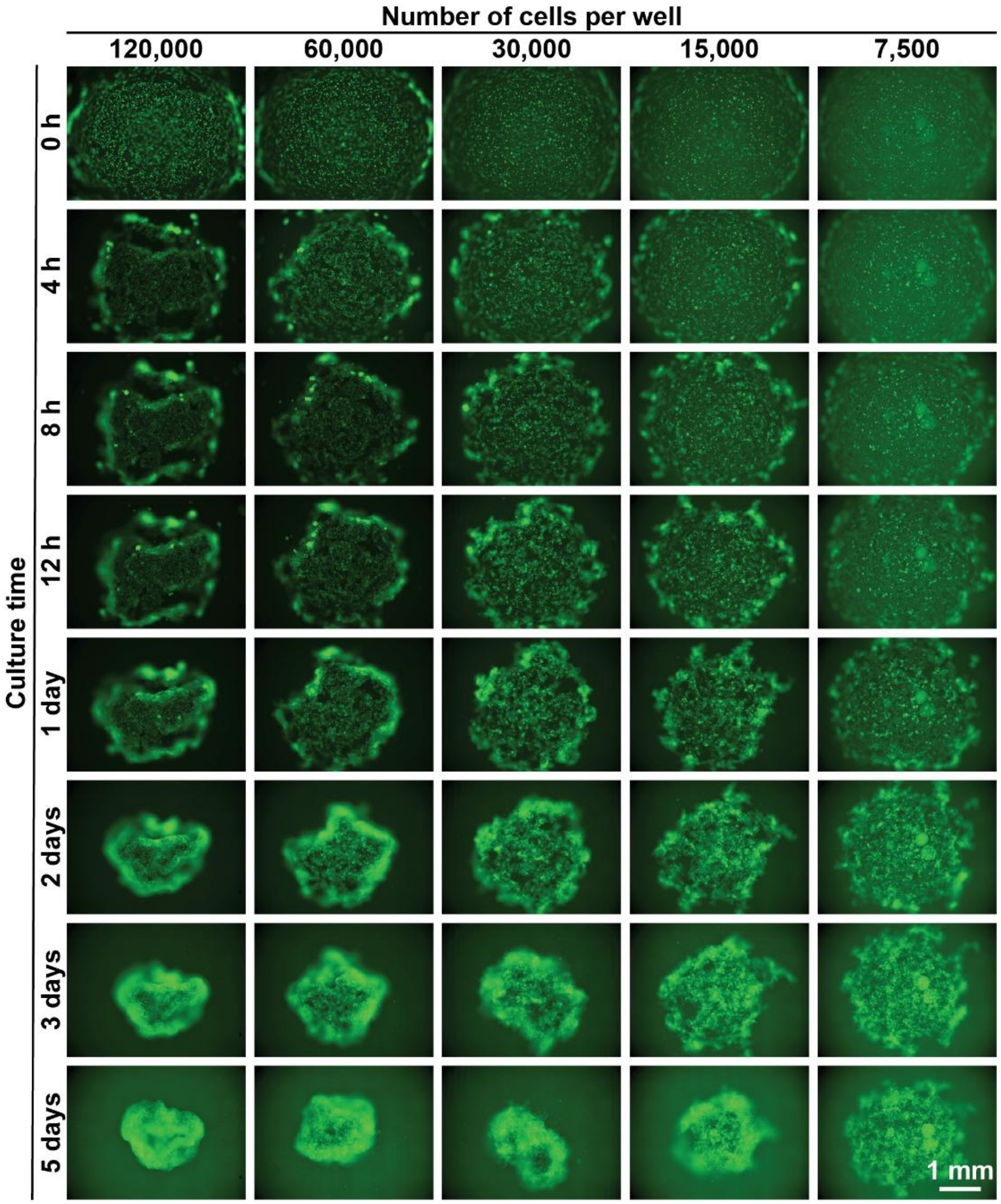

Figure S20. Fluorescence images of BHS-M formation at varying cell seeding densities over 5 days. Microgel amount is constant (1.08 μL of packed medium microgels, centrifuged at 3,000 ×***g*** for 15 s) in each well (96-well plate).

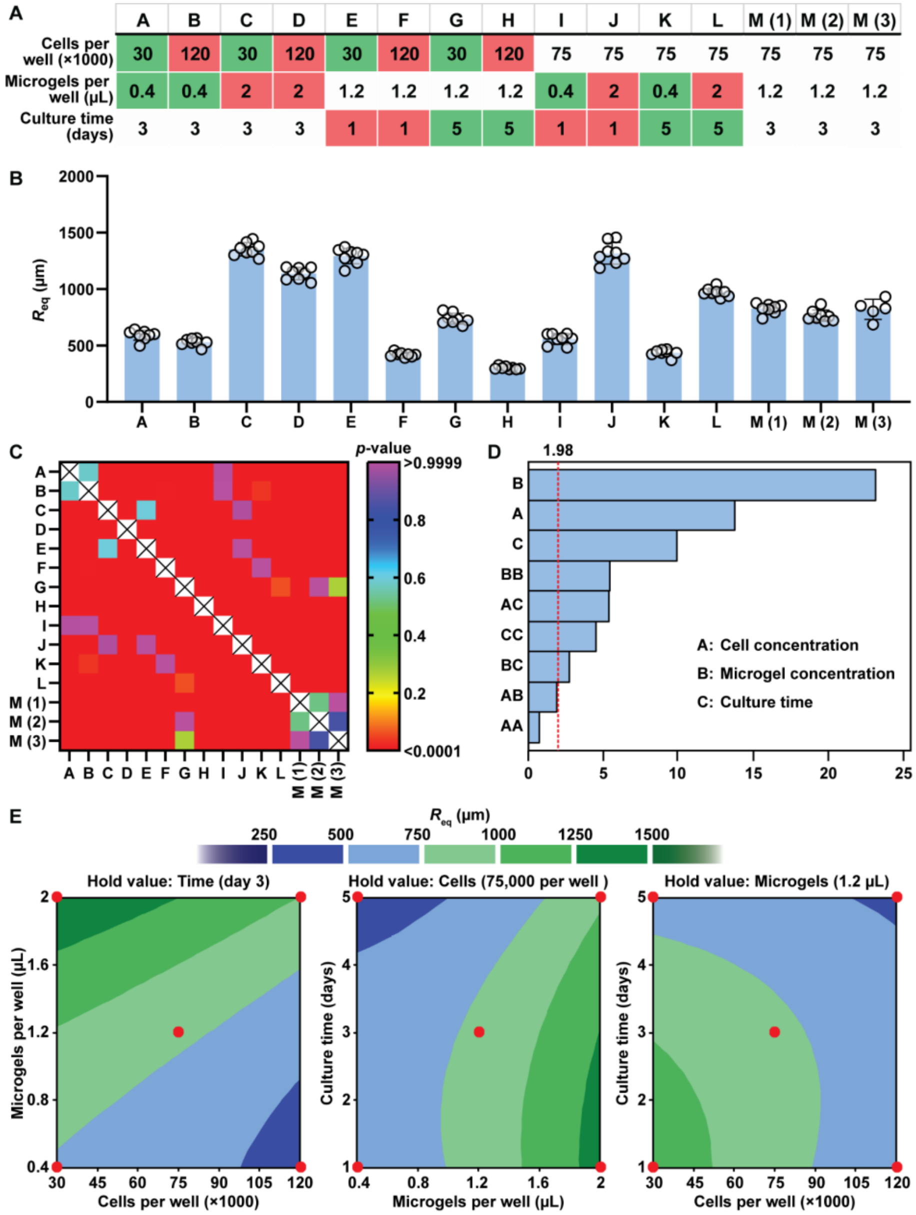

Figure S21. Box-Behnken DOE of BHS formation in a geometrically constrained environment. (A) The DOE layout with 15 experimental groups based on cell concentration (density), concentration of medium microgels, and culture duration. **(B)** The size of formed BHS corresponding to each experimental group ($n > 5$). **(C)** The p-values of Tukey's post-hoc multiple comparison test following the one-way ANOVA, corresponding to panel B. **(D)** Pareto chart of standardized effects. **(E)** Contour plots of BHS formation factors. Data points represent the experimental results.

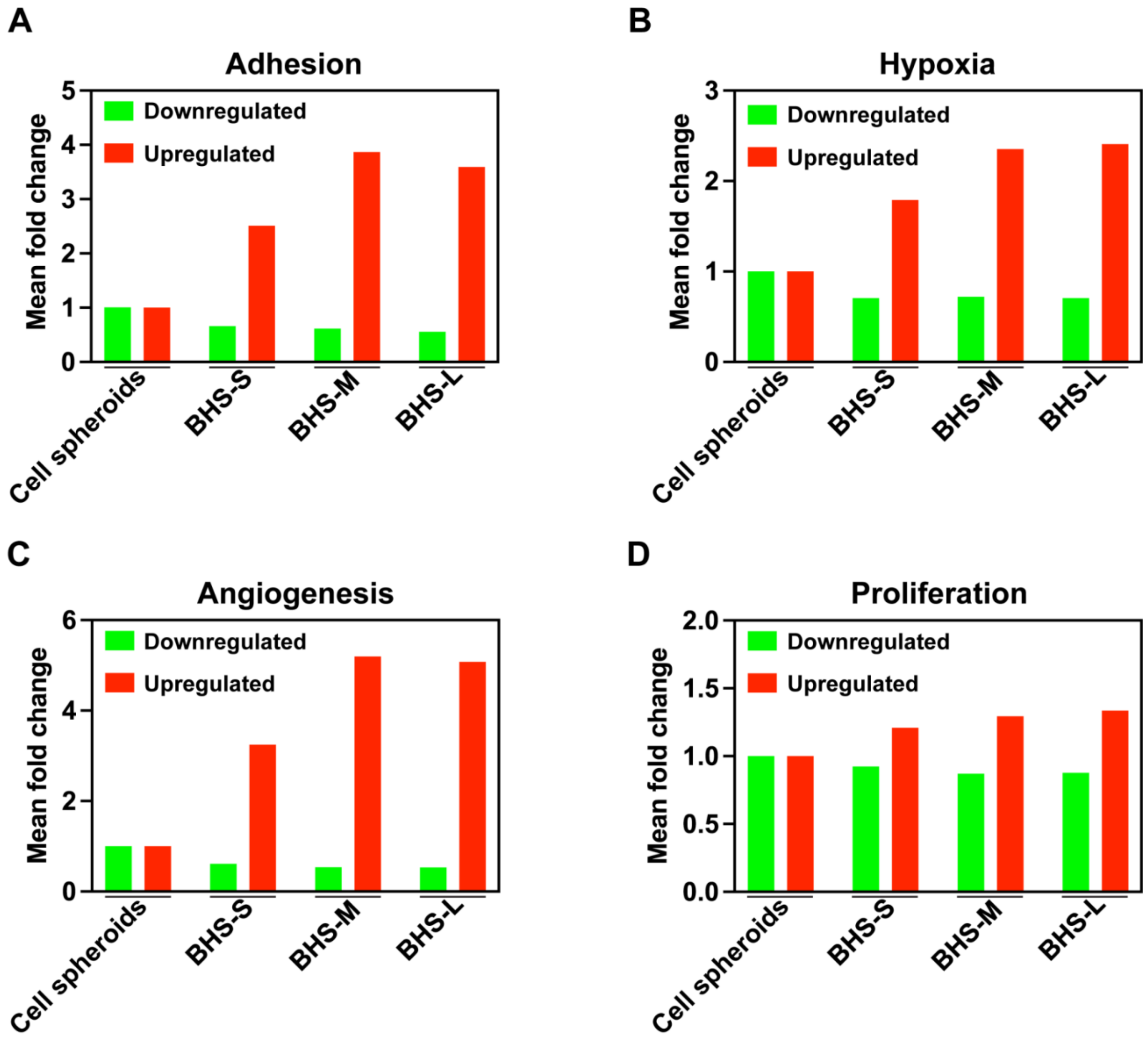

Figure S22. Mean fold change of upregulated and downregulated genes, normalized to cell spheroids, related to **(A)** adhesion, **(B)** hypoxia, **(C)** angiogenesis, and **(D)** proliferation.

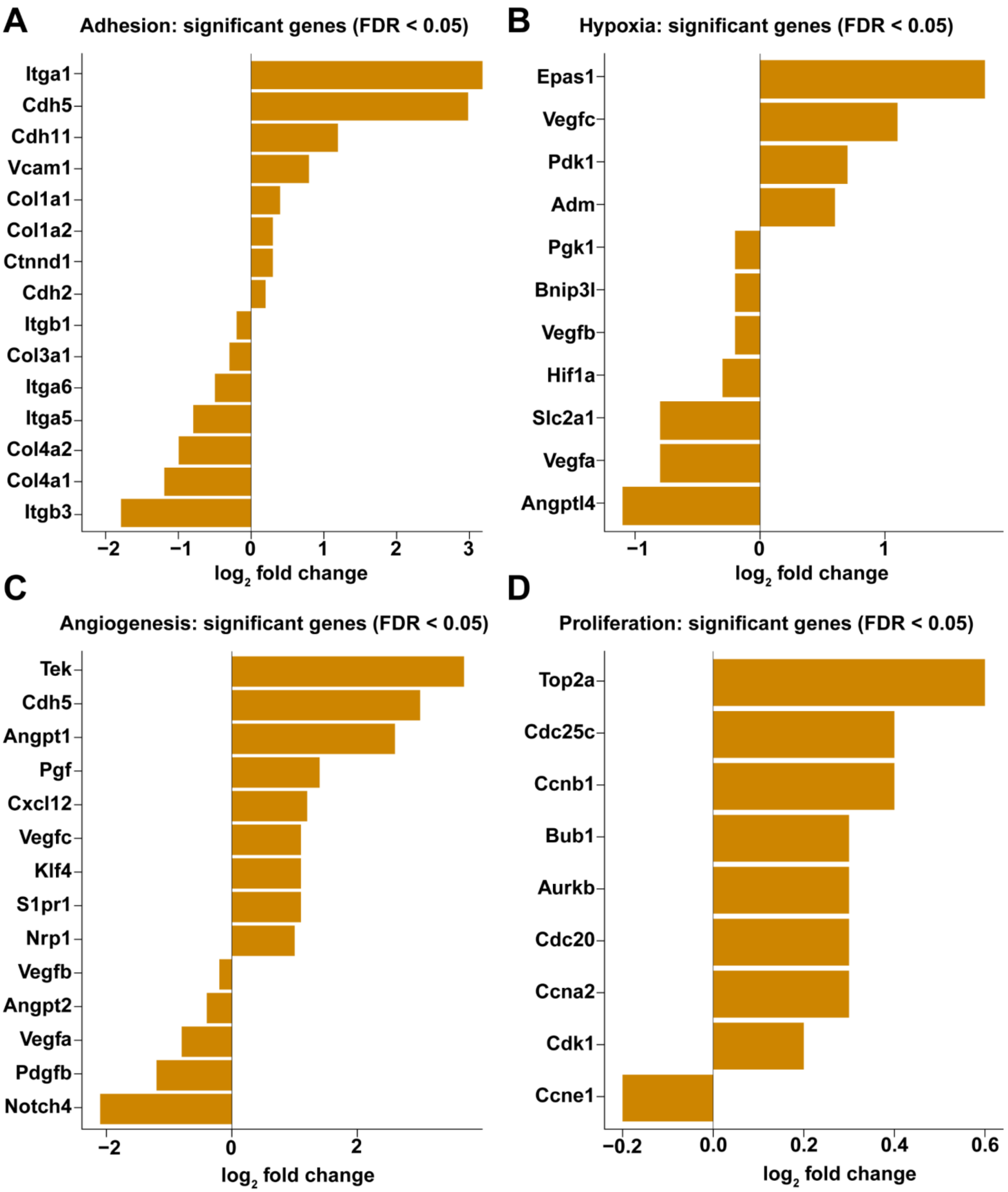

Figure S23. Significant DE genes of BHS, relative to cell spheroids. Log_2 fold changes of the genes involved in specific functions/states, including **(A)** adhesion, **(B)** hypoxia, **(C)** angiogenesis, and **(D)** proliferation.

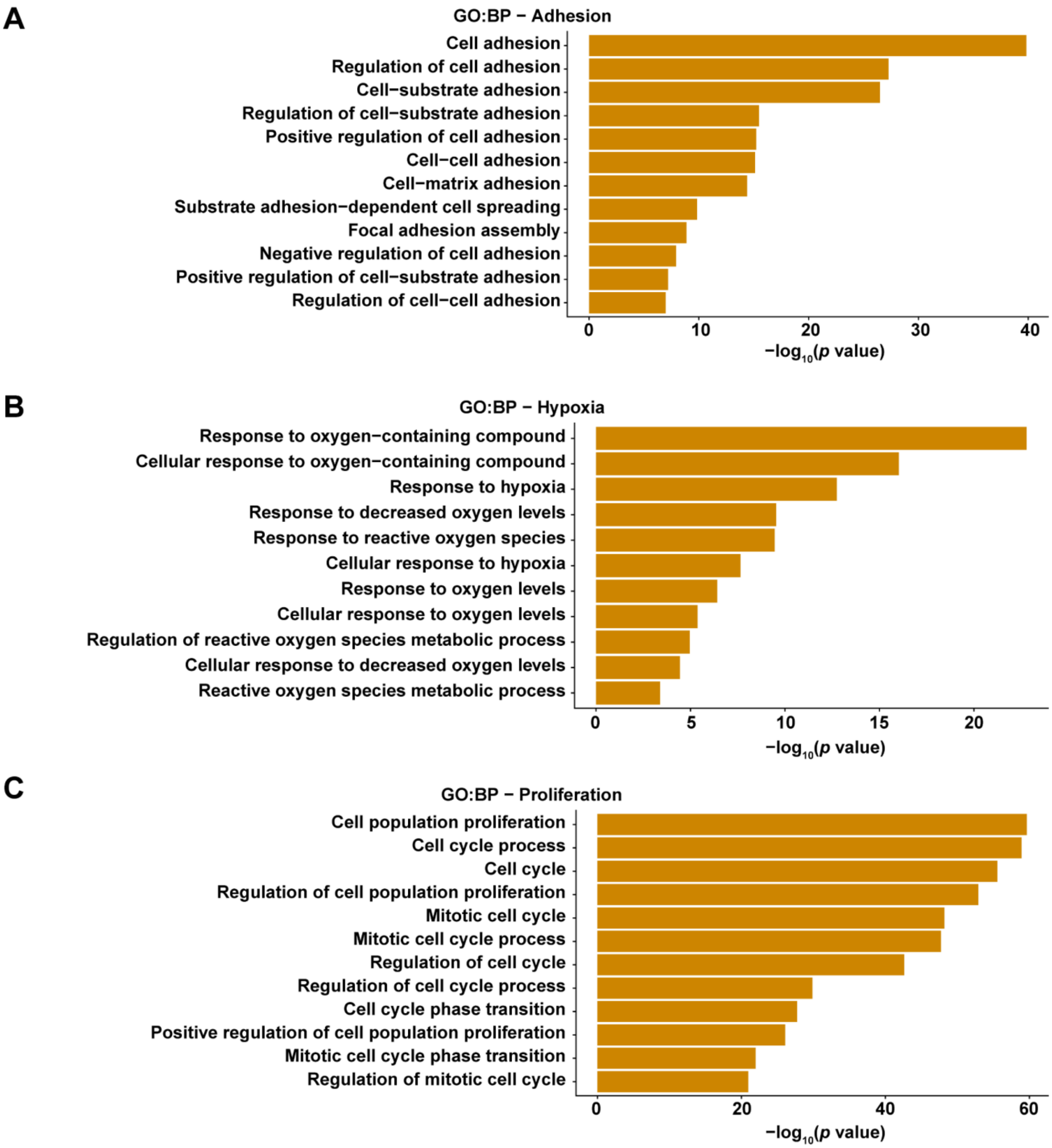

Figure S24. Enriched Gene Ontology (GO) biological process (BP) terms for BHS, relative to cell spheroids. The figure shows terms enriched for specific functions/states, including **(A)** adhesion, **(B)** hypoxia, and **(C)** proliferation.

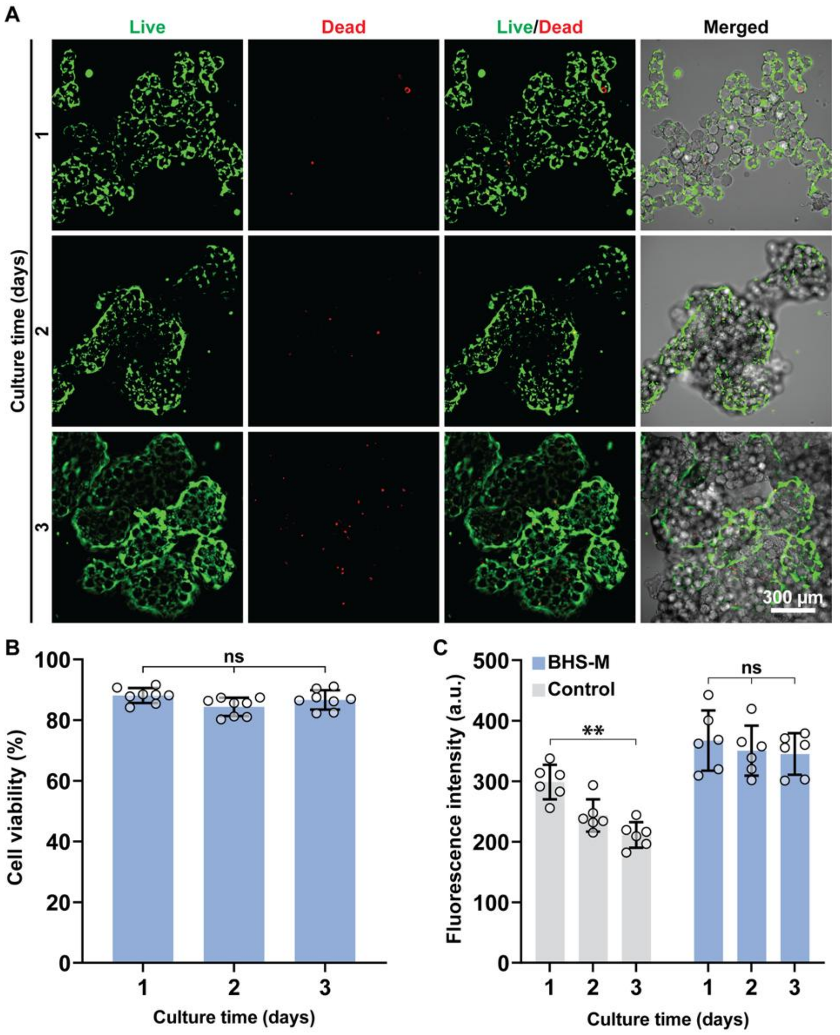

Figure S25. BHS-M *in vitro* characterizations via cell viability and metabolic activity assessments. (A) Cell viability is studied using the Live/Dead staining in three consecutive days. Fluorescence images of BHS show live (green) and dead (red) cells. **(B)** Cell viability, quantified based on the ratio of live cells to the total number of cells, showing ~ 90% viability (n = 8). Ordinary one-way ANOVA, followed by Tukey's post-hoc multiple comparison test are performed (ns = not significant with $p \geq 0.05$). **(C)** Metabolic activity, measured using the PrestoBlue assay. Results show no significant changes in the metabolic activity of cells within BHS-M in 3 days (n = 6). Two-way ANOVA, followed by Tukey's post-hoc multiple comparison test are performed (ns = not significant with $p \geq 0.05$ and $^{**}p < 0.01$).

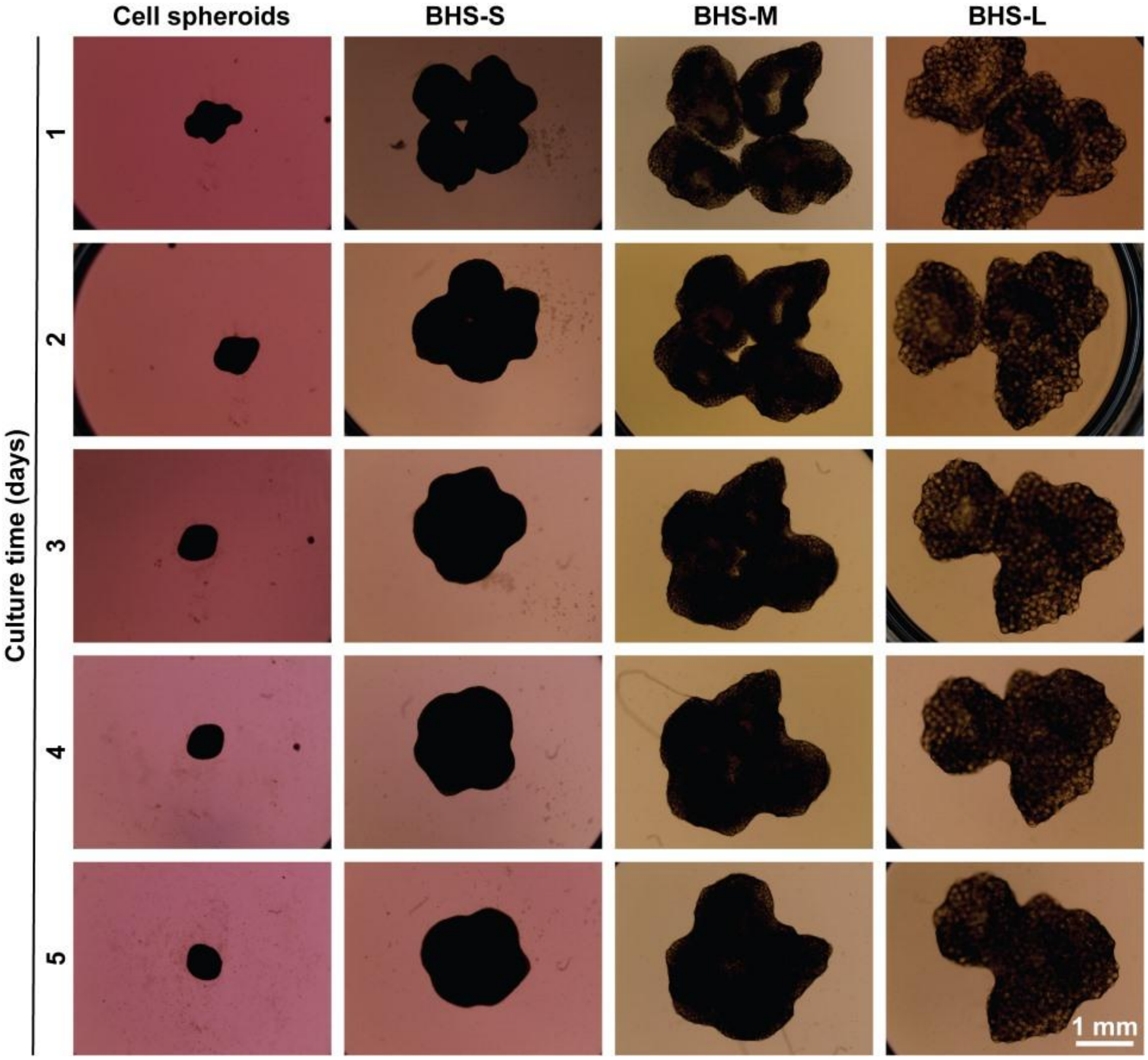

Figure S26. Microscopy images of cell spheroid or BHS fusion. Sequential brightfield images, showing the merging process of BHS or cell spheroids over time. The fusion takes place in a geometrically constrained environment.

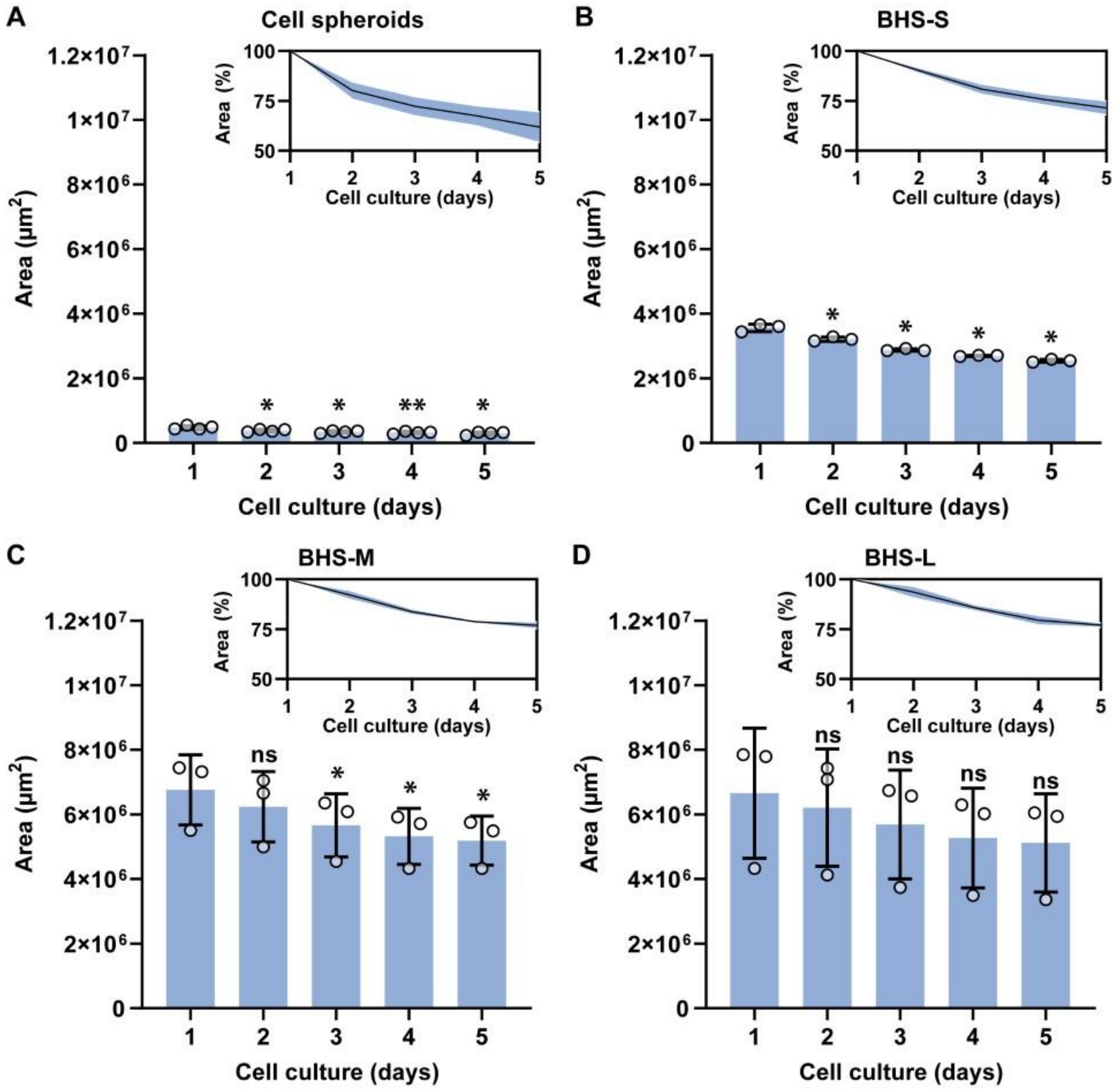

Figure S27. Quantification of BHS and cell spheroid fusion. Detailed statistical analysis of BHS fusion and size variation in **(A)** cell spheroids (n = 4), **(B)** BHS-S (n = 3), **(C)** BHS-M (n = 3), and **(D)** BHS-L (n = 3). Insets show the average area of each study group over time, presented as a percentage of area on day 1. One-way RM ANOVA, followed by Tukey's post-hoc multiple comparison test are performed, and each day is compared with day 1 (ns = not significant with $p \geq 0.05$, $^*p < 0.05$, and $^{**}p < 0.01$).

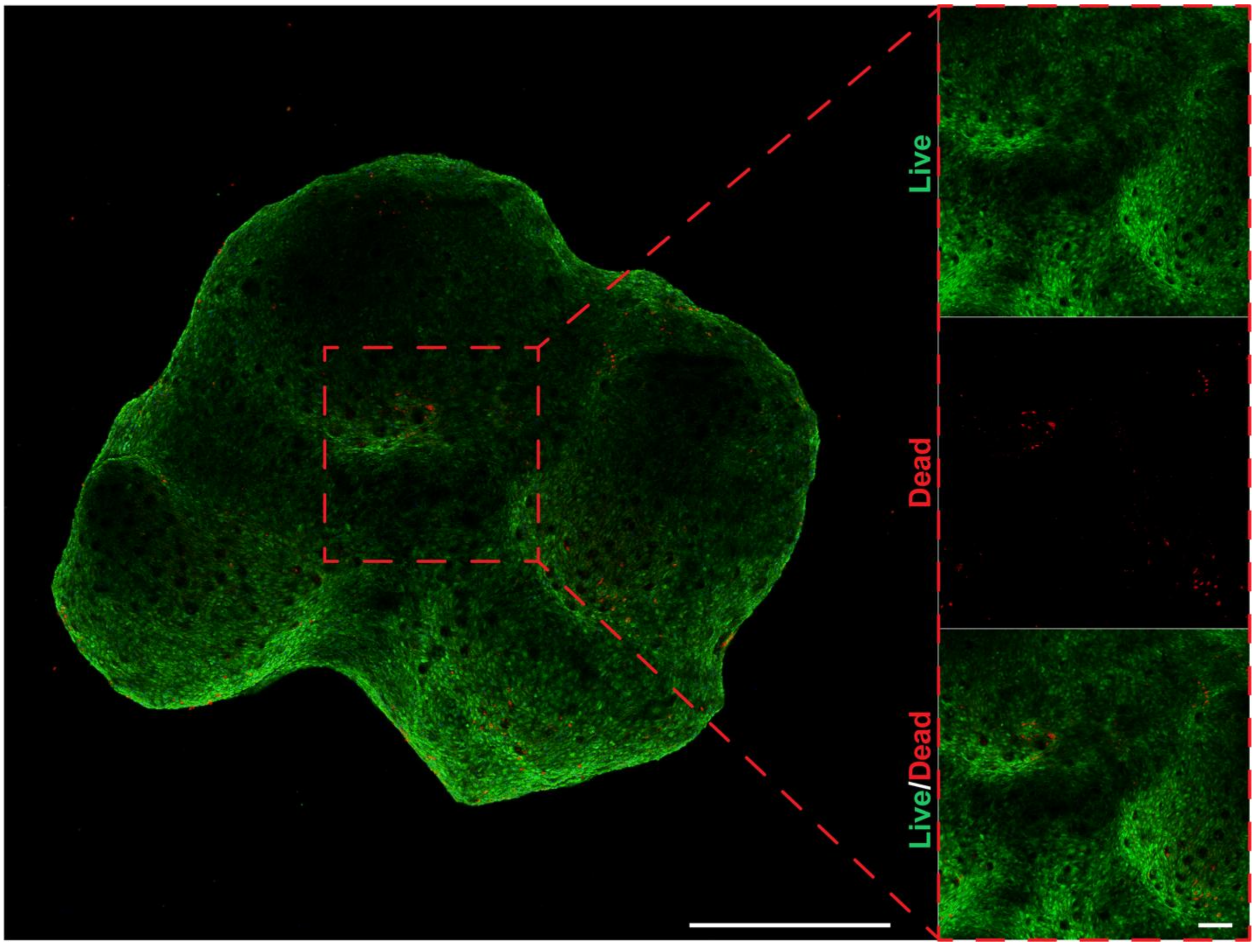

Figure S28. Cell viability assessment in fused BHS after 2 weeks of culture. Scale bar for the main image is 1 mm and for the magnified images on the right is 200 μm.

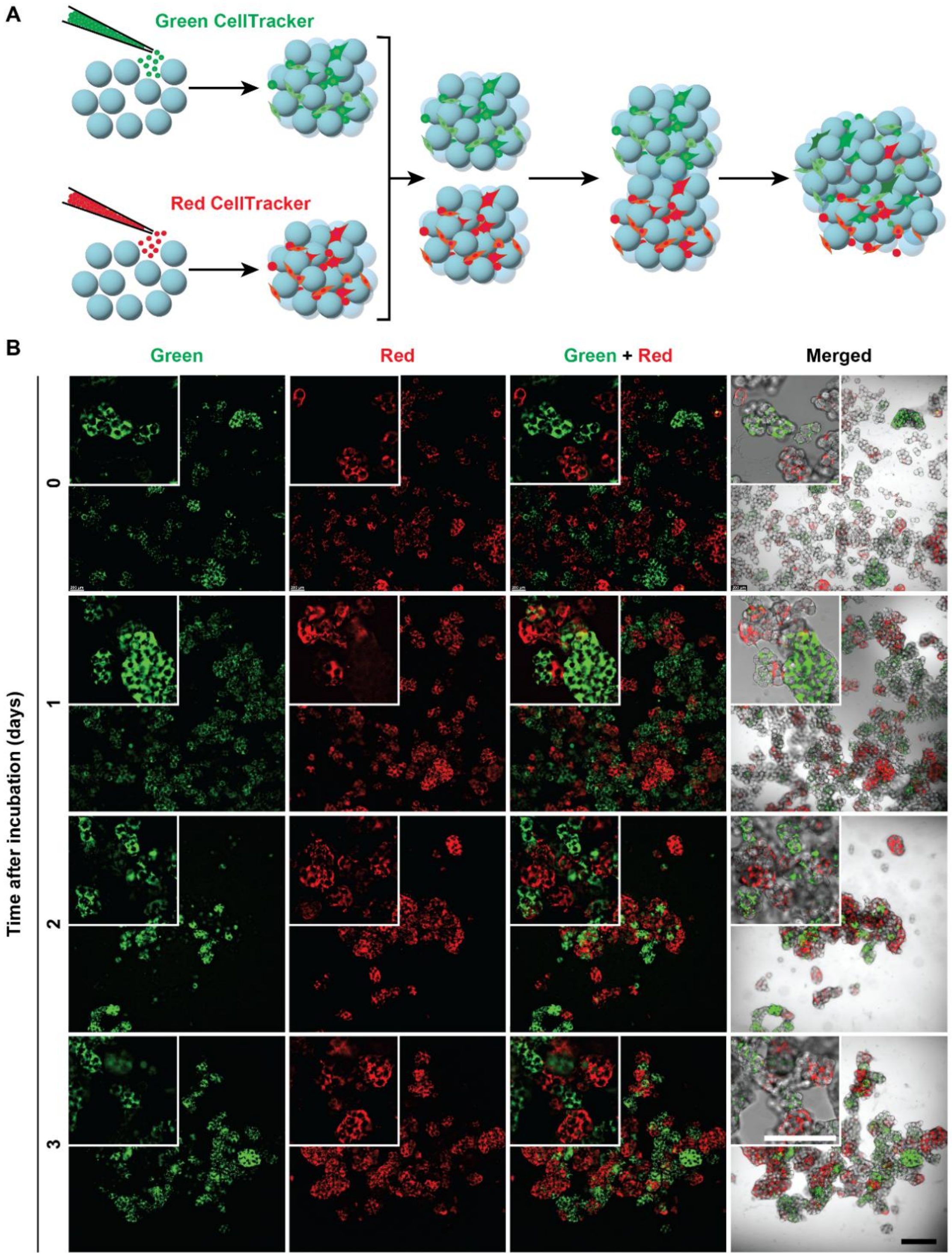

Figure S29. BHS fusion and modularity. (A) Schematic of NIH/3T3 fibroblast cells, stained with green and red fluorophores, followed by BHS formation and fusion. **(B)** Aggregates are mixed (day 0) with limited contact points, which completely merge on day 3. Scale bar is 500 μm.

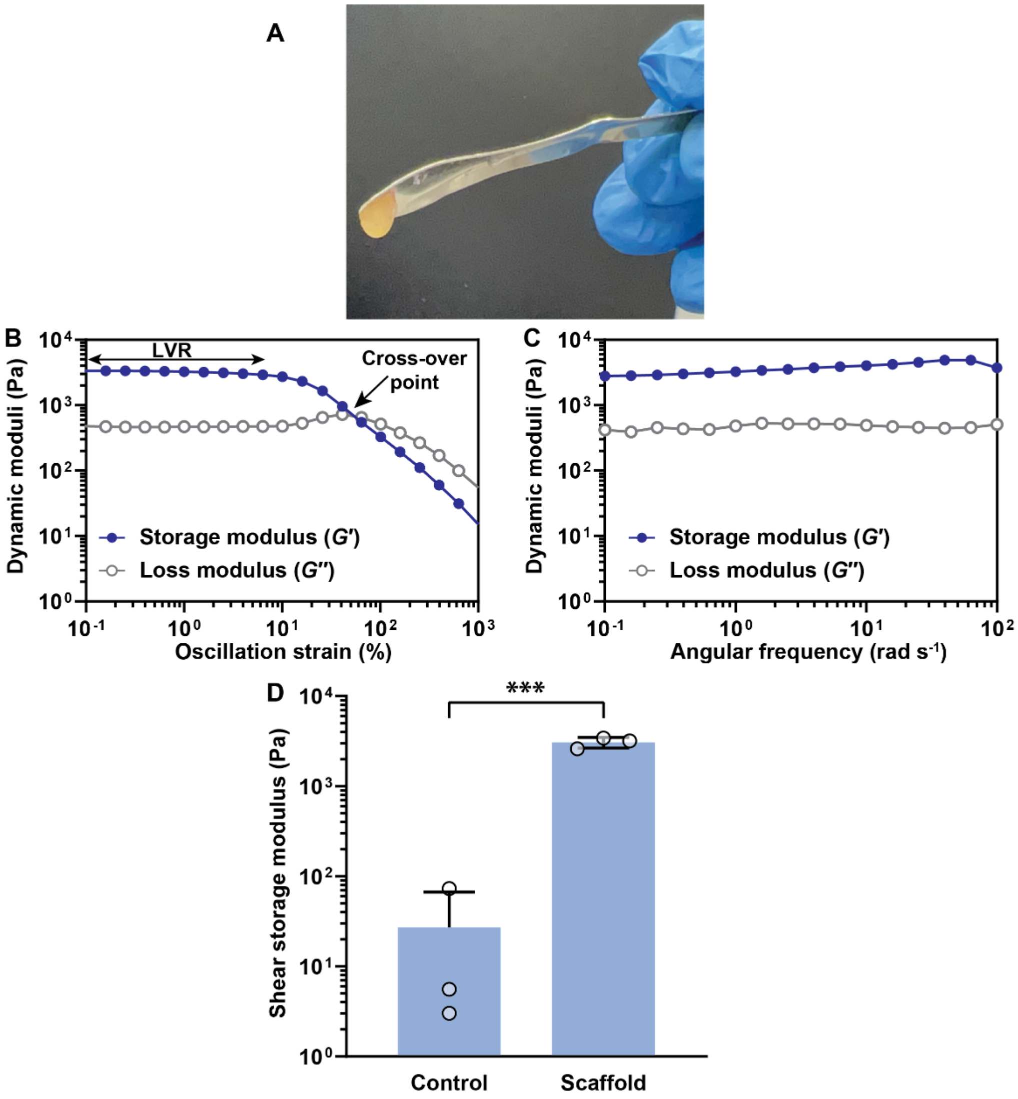

Figure S30. BHS fusion yields viscoelastic tissue-like constructs in 72 h. (A) Formation of a mechanically robust tissue-like construct via BHS fusion. **(B)** Oscillatory strain (amplitude) sweep, representing storage (*G′*) and loss (*G″*) moduli of fused BHS versus strain at a constant frequency of 1 rad s^{-1}. The linear viscoelastic region (LVR) and cross-over point are marked. **(C)** *G′* and *G″* of the fused BHS versus angular frequency at constant strain of 0.1%. **(D)** *G′* and *G″* for each sample, obtained at an angular frequency of 1 rad s^{-1} and oscillatory strain of 0.1%. The control sample consists of a cell-free microgel suspension ($n = 3$). Unpaired two-tailed *t*-test is performed (***p<0.001).

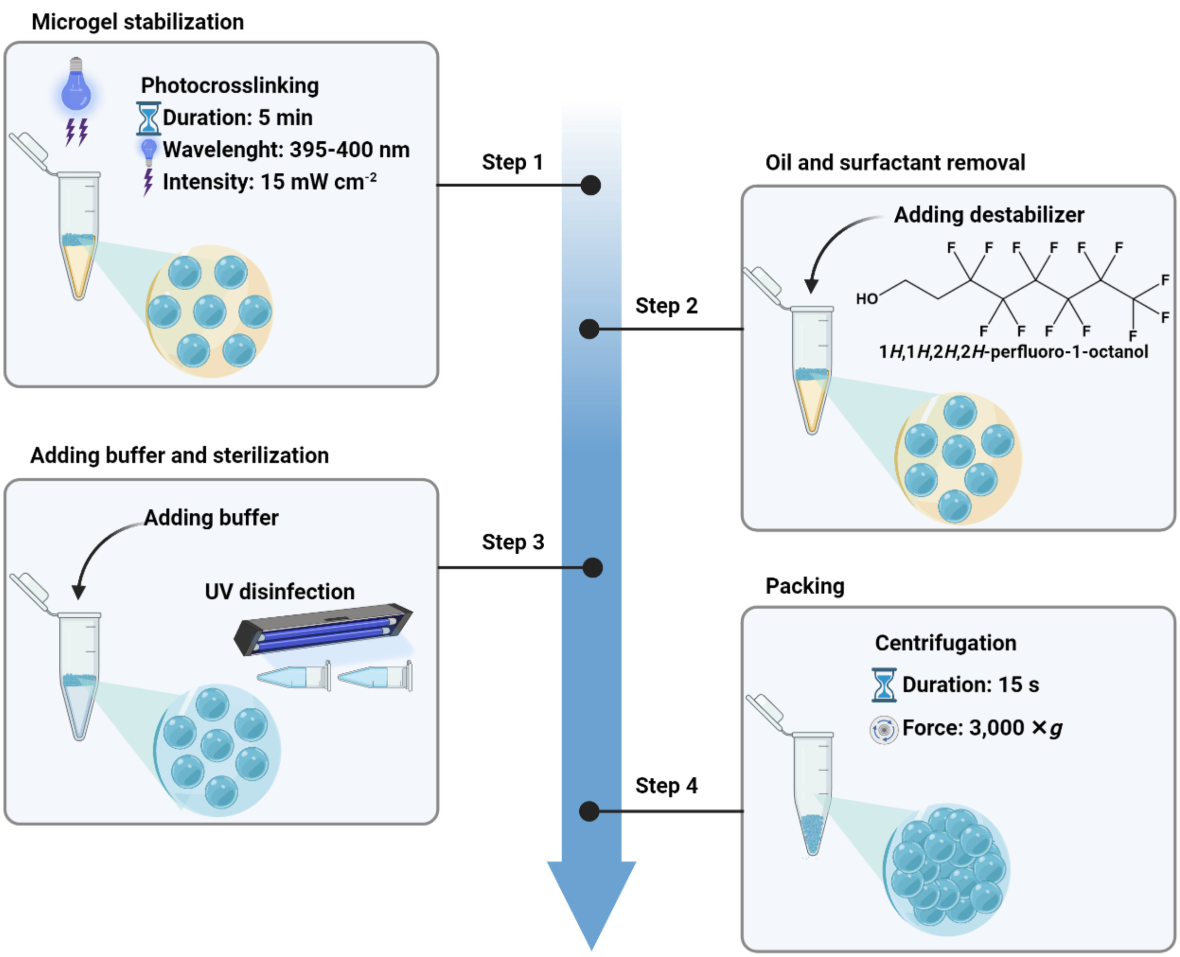

Figure S31. GelMA microgel preparation protocol. Step 1: GelMA droplets are photocrosslinked under light with a wavelength of 395-400 nm and an intensity of 15 mW cm^{-2} for 5 min. **Step 2:** Microgels are purified from the oil and surfactant using a PFO solution (20% v/v in Novec™ 7500 Engineered Fluid). **Step 3:** Microgels are diluted and hydrated using DPBS, supplemented with 2% v/v of penicillin-streptomycin, followed by UV disinfection at a wavelength of 254 nm for 2 h. **Step 4:** The microgel suspension is packed by centrifugation at 3,000 ×***g*** for 15 s, yielding a jammed suspension.

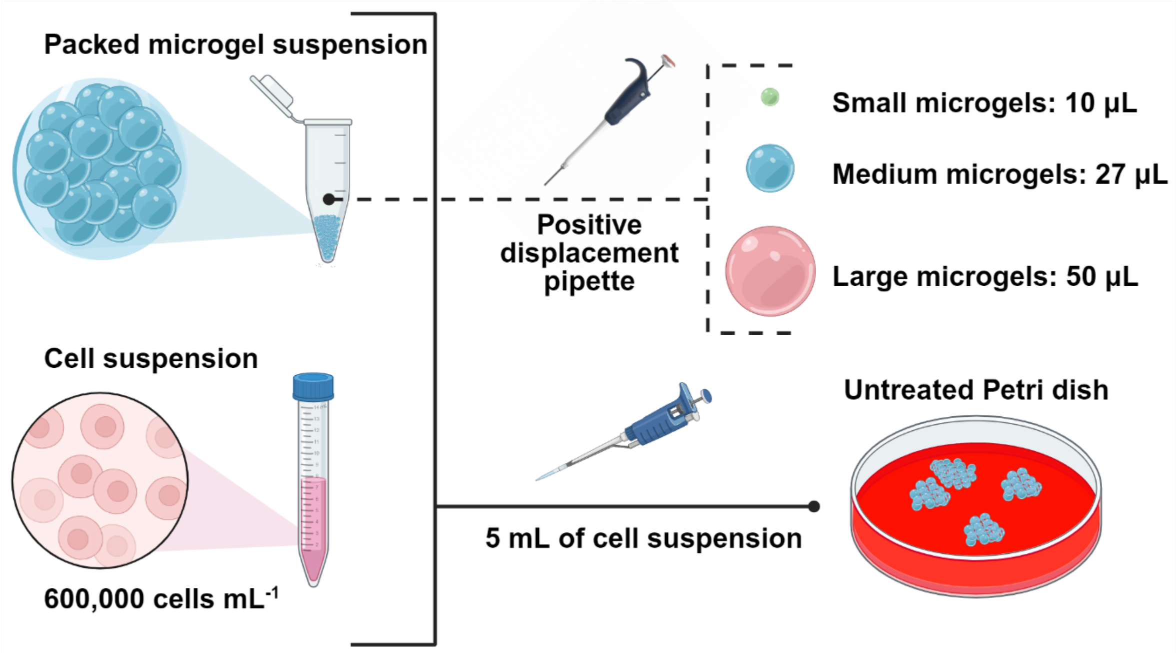

Figure S32. Method of BHS formation via cell-mediated microgel assembly in a geometrically unconstrained environment. The microgel suspension is introduced into a non-treated Petri dish, containing 5 mL of media and 600,000 cells mL^{-1}, totaling 3,000,000 cells. Subsequently, the microgels and cells are cultured together for 3 days.

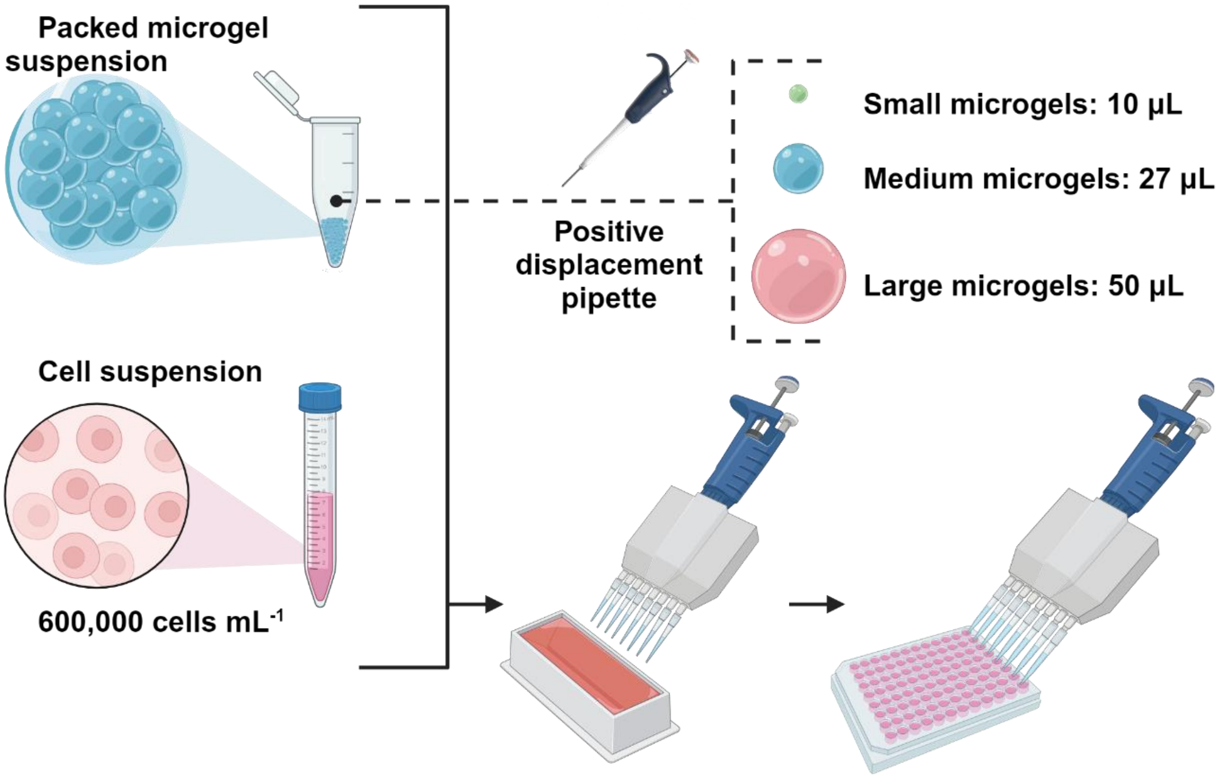

Figure S33. Method of BHS formation under geometric constraints via cell-mediated microgel assembly in a geometrically constrained environment. The microgel suspension is added to the media containing 600,000 cells mL^{-1}, followed by dispensing 200 µL of the mixed cell-microgel suspension into each well.

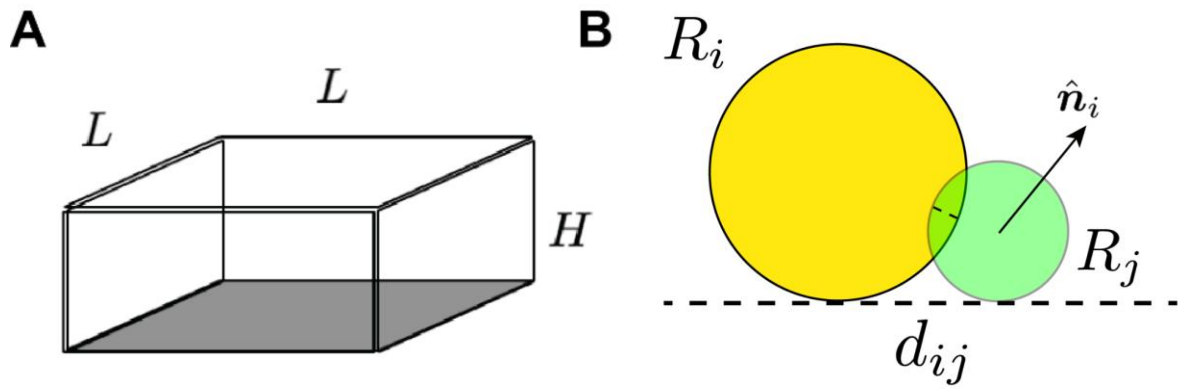

Figure S34. A schematic illustration of ABM features. **(A)** A cuboid simulation box with size $L \times L \times H$ is used to approximate the representative area of culture medium, where the periodic boundary condition is set on the *x*-*y* direction, and a rigid substrate exists on the plane *z* = 0. **(B)** A schematic illustration of the ABM. The green sphere denotes cells with the radius $\boldsymbol{R_i}$ and director $\hat{\boldsymbol{n}}_{\boldsymbol{i}}$. The yellow sphere denotes microgels with the radius $\boldsymbol{R_j}$. The elastic contact and adhesion are governed by the overlapping distance $d_{\boldsymbol{ij}}$.

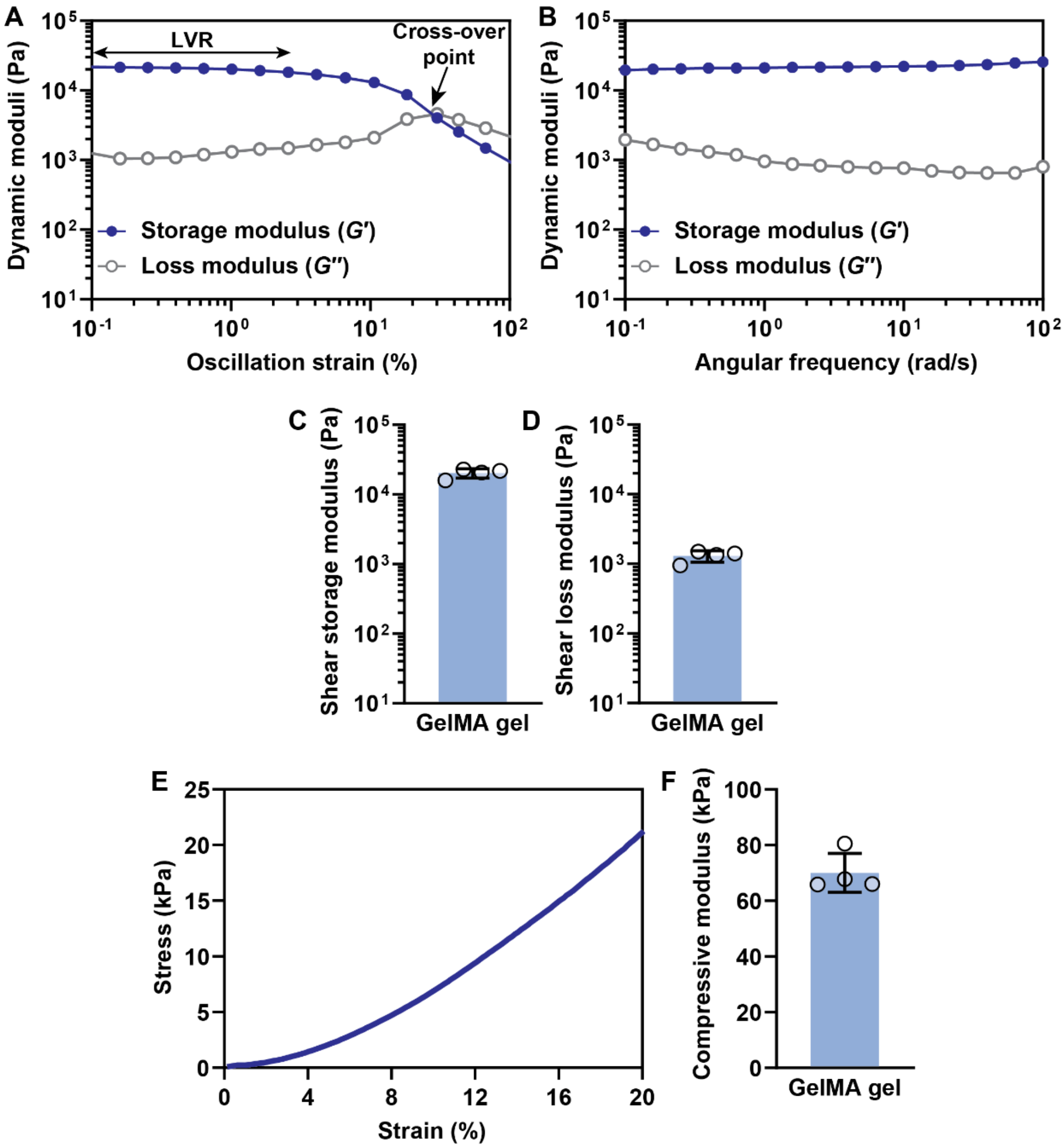

Figure S35. Viscoelastic and mechanical characterization of bulk GelMA hydrogel scaffold. **(A)** Dynamic moduli as a function of oscillatory strain at constant frequency of 1 rad s^{-1}, showing the LVR and cross-over point. **(B)** Frequency sweep, showing dynamic moduli versus angular frequency at constant strain of 0.1%, with *G*' remaining predominantly higher than *G*'', indicative of a solid-like behavior. **(C)** *G*' and **(D)** *G*'' of bulk GelMA hydrogel at 0.1% strain and 1 rad s^{-1} frequency ($n = 4$). **(E)** Compressive stress-compressive strain curve. **(F)** Compressive modulus of bulk GelMA hydrogel ($n = 4$).

Table S1. Microparticle-cell mixtures for living construct formation.

Reference	Microparticle material(s)	Cells	Main objective(s)
**This work**	GelMA	NIH/3T3<br>MSC<br>HUVEC	Develop and mechanistically understand biohybrid spheroids (BHS), self-assembled living-synthetic 3D aggregates formed by adherent cells and ECM-mimetic hydrogel microgels, to overcome diffusion limitations and poor cell viability in conventional cell spheroids. By tuning microgel size and cell-to-microgel ratios, the work seeks to control BHS assembly kinetics, size, and porosity, enabling the formation of large, diffusion-permissive constructs with improved cell survival. The study further aims to elucidate how BHS alter cellular transcriptional programs and to establish an agent-based model that captures the underlying assembly mechanisms, providing design principles for scalable, physiologically relevant *in vitro* tissue models for NAMs and biofabrication applications.

**Chung *et al.* (2024)[2]**	Collagen<br>HA	MSC	Develop collagen microgels as a cell-assembling platform to enhance cell survival, angiogenic potential, and therapeutic efficacy in treating critical limb ischemia (CLI). Microgels, fabricated through the micro-fragmentation of Collagen-HA gels, and human adipose-derived MSC form 3D constructs to improve regeneration in CLI models.
**Rovers *et al.* (2024)[3]**	UPy-based	NHDF	Develop modular UPy-based supramolecular microgels held by non-covalent interactions, creating tunable dynamic systems for enhanced cellular interactions in tissue engineering.
**Bulut *et al.* (2023)[4]**	Dex-MA	NHDF	Investigate how NHDF self-organize Dex-MA microgels into scaffolds without the need for chemical crosslinkers, enabling the formation of dynamic, physiologically relevant 3D structures. Additionally, show how microgel stiffness, cell/microgel ratio, and scaffold geometry influence cell behavior, scaffold morphology, and the

			mechanical properties of assemblies.
**Nelson *et al.* (2023)[5]**	PEG-dithiolane	C2C12	Explore dynamic disulfide crosslinking in PEG-dithiolane microgels to reprogram microgel shapes into disk geometries with high-curvature and flattened regions. These anisotropic features influence C2C12 myoblast localization and behavior, driving curvature-dependent assembly of supraparticle scaffolds.
**Kamperman *et al.* (2023)[6]**	Dex-TAB functionalized with RGD	MSC	Develop cell-adhesive micromaterials using RGD-functionalized Dex-TAB microgels that autonomously assemble with MSC into 3D microtissues, offering tunable biochemical and biomechanical cues to steer MSC differentiation toward adipogenesis or osteogenesis.
**Cunha *et al.* (2022)[7]**	Collagen-coated PS	MSC	Examine how collagen-coated PS microparticle size in free-packed granular beds influences cell attachment, spreading, and detachment, revealing that smaller particles (14-20 µm) promote apoptosis and detachment, while larger

			particles (38-105 µm) support stable adhesion, proliferation, and aggregate stability.
**Fois *et al.* (2022)[8]**	Ti<br>Cu<br>PS<br>PMMA<br>TCP	MSC<br>MG63	Assess cell-material interactions by co-aggregating micron-sized biomaterials into tissue spheroids to evaluate adhesion, viability, and differentiation. Using a library of biomaterials, the study reveals how material properties and cell-material ratios influence spheroid formation, metabolic activity, and osteogenic differentiation.
**Nagarajan *et al.* (2021)[9]**	PS<br>Silica	S180	Investigate hybrid aggregates of cells and microparticles to understand how size, density, and volume fraction influence aggregate dynamics and mechanical properties. Lighter microparticles are pushed outward, forming rims that decelerate spreading, while heavier microparticles enable cell spreading and create heterogeneous particle distributions.
**Neto *et al.* (2021)[10]**	PCL	MSC<br>MC3T3-E1	Fabricate 2D microparticles with precise shapes and wettability to evaluate their effects on cell behavior and growth factors secretion. When

			integrated with cells, these microparticles enhance metabolic activity, compactness, and pro-angiogenic factor release without disrupting spheroid formation or morphology.
**Zhang *et al.* (2019)[11]**	PVCL<br>OEGA<br>GMA	HeLa	Investigate how microgel size, crosslinking density, and surface structure influence penetration into tumor spheroids and cellular uptake, with potential cancer therapy applications.
**Ferreira *et al.* (2018)[12]**	HA-coated PCL	A549<br>HF<br>MSC	Develop bio-instructive microparticles coated with hyaluronan to mimic tumor microenvironments, studying cellular interactions, drug resistance, and 3D tumor spheroid formation.
**Yajima *et al.* (2017)[13]**	Collagen	NIH/3T3<br>HepG2	Develop thick, porous tissues using collagen microparticles as scaffolds, addressing hypoxia and enhancing cell viability in densely packed 3D constructs.
**Leferink *et al.* (2014)[14]**	SU-8	MSC<br>C2C12<br>ATDC5	Investigate how SU-8 micro-objects with defined shapes and tunable properties enhance compaction, mechanical properties, and uniform cell distribution in tissue

			constructs. These micro-objects promote cell adhesion, spreading, and ECM deposition, enabling controlled 3D aggregation and modulation of tissue geometry and complexity.
**Hayashi *et al.* (2011)[15]**	Gelatin<br>PLGA<br>Agarose	MSC	Improve the viability and osteogenic differentiation of MSCs by incorporating gelatin hydrogel microspheres, enhancing oxygen and nutrient diffusion within 3D aggregates, and influencing biological functions such as proliferation and differentiation.
**Matsunaga *et al.* (2011)[16]**	Collagen	NIH 3T3<br>HepG2<br>HUVEC<br>Primary neurons<br>Primary rat hepatocytes,<br>MIN6-m9	Develop collagen gel-based microtissue units for the rapid construction of macroscopic 3D tissue architectures with functional cell-cell interactions and ECM integration. The approach uses cell beads stacked into molds to form dense, necrosis-free tissues with uniform cell distribution, offering scalability, adaptability to diverse cell types, and geometrical control for bottom-up tissue engineering.

**Bratt-Leal *et al.* (2011)[17]**	Agarose<br>PLGA<br>Gelatin	PSC	Investigate how embedding microparticles in PSC aggregates spatially controls environmental cues, influencing differentiation pathways and enabling targeted tissue formation.
**Shim *et al.* (2024)[18]**	HA	Chondrocytes	Exploring the incorporation of HA microparticles within chondrocyte spheroids, enhancing cartilage regeneration by maintaining chondrocyte function, improving cell survival, and supporting *in vivo* tissue formation.
**Kim *et al.* (2022)[19]**	PCL	Human bone marrow-derived MSC	Investigate the incorporation of bioactive molecule bone morphogenetic protein-2, (BMP-2)-immobilized PCL particles within cell spheroids. The inclusion of these particles prevents cellular heterogeneity by enhancing oxygen and nutrient diffusion, while their cell-adhesive surfaces improve structural stability. Furthermore, the sustained release of BMP-2 from the particles induces the osteogenic differentiation of stem cells and promotes new bone formation.

Cell abbreviations

A549: Human lung cancer cells

ATDC5: Mouse pre-chondrogenic cells

C2C12: Mouse myoblast cell line

HeLa: Human cervical cancer cells

HF: Human fibroblasts

HepG2: Human hepatocarcinoma cells

HUVEC: Human umbilical vein endothelial cells

MC3T3-E1: Mouse pre-osteoblast cell line

MG63: Human osteoblast-like cells

MIN6-m9: Mouse pancreatic beta cells

MSC: Mesenchymal stem cells

NHDF: Normal human dermal fibroblasts

NIH/3T3: Mouse embryonic fibroblast cells

PSC: Pluripotent stem cells

S180: Mouse sarcoma cells

Material abbreviations

Cu: Copper

Dex: Dextran

GelMA: Gelatin methacryloyl

GMA: Glycidyl methacrylate

HA: Hyaluronic acid

OEGA: Oligo(ethylene glycol) acrylate

PCL: Polycaprolactone

PEG: Polyethylene glycol

PLGA: Poly(lactic-co-glycolic acid)

PMMA: Poly(methyl methacrylate)

PS: Polystyrene

PVCL: Poly(N-vinylcaprolactam)

RGD: Arginine-glycine-aspartic acid peptides

TAB: Triarylborane

TCP: Tricalcium phosphate

Ti: Titanium

UPy: Ureido-pyrimidinone

List of supporting videos

Video S1 (separate file). BHS formation in a geometrically unconstrained environment via cell-mediated microgel assembly using NIH/3T3 fibroblast cells and small microgels.

Video S2 (separate file). BHS formation in a geometrically unconstrained environment via cell-mediated microgel assembly using NIH/3T3 fibroblast cells and medium microgels.

Video S3 (separate file). BHS formation in a geometrically unconstrained environment via cell-mediated microgel assembly using NIH/3T3 fibroblast cells and large microgels.

Video S4 (separate file). Control experiment in a geometrically unconstrained environment using cell-free medium microgels, showing no movement.

Video S5 (separate file). Control experiment in a geometrically unconstrained environment using only NIH/3T3 cells, showing cell spheroid formation.

Video S6 (separate file). BHS formation in a geometrically constrained environment via cell-mediated microgel assembly using NIH/3T3 fibroblast cells and small microgels.

Video S7 (separate file). BHS formation in a geometrically constrained environment via cell-mediated microgel assembly using NIH/3T3 fibroblast cells and medium microgels.

Video S8 (separate file). BHS formation in a geometrically constrained environment via cell-mediated microgel assembly using NIH/3T3 fibroblast cells and large microgels.

Video S9 (separate file). Control experiment in a geometrically constrained environment using only NIH/3T3 cells showing cell spheroid formation.

Video S10 (separate file). Control experiment in a geometrically constrained environment using cell-free medium microgels, showing no movement.

Video S11 (separate file). Pipetting tissue-like constructs, formed from BHS-M fusion after five days of culture.

Video S12 (separate file). Pipetting the tissue-like constructs that formed via BHS-M fusion after two weeks of culture.

References

[1] K. Yue, X. Li, K. Schrobback, A. Sheikhi, N. Annabi, J. Leijten, W. Zhang, Y. S. Zhang, D. W. Hutmacher, T. J. Klein, A. Khademhosseini, *Biomaterials* 2017, *139*, 163.

[2] H. Chung, J. K. Choi, C. Hong, Y. Lee, K. H. Hong, S. J. Oh, J. Kim, S. C. Song, J. W. Kim, S. H. Kim, *Bioact Mater* 2024, *34*, 80.

[3] M. M. Rovers, T. Rogkoti, B. K. Bakker, K. J. Bakal, M. H. P. van Genderen, M. Salmeron-Sanchez, P. Y. W. Dankers, *Advanced Materials* 2024, *36*, 2405868.

[4] S. Bulut, D. Günther, M. Bund, C. Haats, T. Bissing, C. Bastard, M. Wessling, L. De Laporte, A. Pich, *Adv Healthc Mater* 2024, *13*, 2302957.

[5] B. R. Nelson, B. E. Kirkpatrick, N. P. Skillin, N. Di Caprio, J. S. Lee, L. P. Hibbard, G. K. Hach, A. Khang, T. J. White, J. A. Burdick, C. N. Bowman, K. S. Anseth, *Adv Healthc Mater* 2024, *13*, 2302925.

[6] T. Kamperman, N. G. A. Willemen, C. Kelder, M. Koerselman, M. Becker, L. Lins, C. Johnbosco, M. Karperien, J. Leijten, *Advanced Science* 2023, *10*, 2205487.

[7] A. F. Cunha, A. F. V. Matias, C. S. Dias, M. B. Oliveira, N. A. M. Araújo, J. F. Mano, *ACS Appl Mater Interfaces* 2022, *14*, 40469.

[8] M. G. Fois, Z. N. Tahmasebi Birgani, A. P. M. Guttenplan, C. A. van Blitterswijk, S. Giselbrecht, P. Habibović, R. K. Truckenmüller, *Small* 2022, *18*, 2202112.

[9] U. Nagarajan, G. Beaune, A. Y. W. Lam, D. Gonzalez-Rodriguez, F. M. Winnik, F. Brochard-Wyart, *Commun Phys* 2021, *4*, 2.

[10] M. D. Neto, A. Stoppa, M. A. Neto, F. J. Oliveira, M. C. Gomes, A. R. Boccaccini, P. A. Levkin, M. B. Oliveira, J. F. Mano, *Advanced Materials* 2021, *33*, 2007695.

[11] C. Zhang, E. Gau, W. Sun, J. Zhu, B. M. Schmidt, A. Pich, X. Shi, *Biomater Sci* 2019, *7*, 4738.

[12] L. P. Ferreira, V. M. Gaspar, J. F. Mano, *Biomaterials* 2018, *185*, 155.

[13] Y. Yajima, M. Yamada, R. Utoh, M. Seki, *ACS Biomater Sci Eng* 2017, *3*, 2144.

[14] A. Leferink, D. Schipper, E. Arts, E. Vrij, N. Rivron, M. Karperien, K. Mittmann, C. van Blitterswijk, L. Moroni, R. Truckenmüller, *Advanced Materials* 2014, *26*, 2592.

[15] K. Hayashi, Y. Tabata, *Acta Biomater* 2011, *7*, 2797.

[16] Y. T. Matsunaga, Y. Morimoto, S. Takeuchi, *Advanced Materials* 2011, *23*, 90.

[17] A. M. Bratt-Leal, R. L. Carpenedo, M. D. Ungrin, P. W. Zandstra, T. C. McDevitt, *Biomaterials* 2011, *32*, 48.

[18] H.-E. Shim, Y.-J. Kim, K. H. Park, H. Park, K. M. Huh, S.-W. Kang, *Carbohydr Polym* 2024, *328*, 121734.

[19] M. J. Kim, Y. J. Jeoung, H. Y. Kim, S. Y. Kim, J. Y. Kim, J. W. Park, J.-H. Byun, J. H. Lee, S. H. Oh, *Chemical Engineering Journal* 2022, *429*, 132590.
